# Specific Fluorescent Probes for Imaging DNA in Cell-Free Solution and in Mitochondria in Living Cells

**DOI:** 10.3390/bios13070734

**Published:** 2023-07-15

**Authors:** Anna S. Efimova, Mariya A. Ustimova, Nelly S. Chmelyuk, Maxim A. Abakumov, Yury V. Fedorov, Olga A. Fedorova

**Affiliations:** 1 Laboratory of Photoactive Supramolecular Systems, A. N. Nesmeyanov Institute of Organoelement Compounds of Russian Academy of Sciences, Vavilova Str. 28, 119334 Moscow, Russia; annkramskaya@gmail.com (A.S.E.); ustimova.maria@yandex.ru (M.A.U.);; 2 Department of Technology of Fine Organic Synthesis and Chemistry of Dyes, Dmitry Mendeleev University of Chemical Technology of Russia, Miusskaya Sqr. 9, 125047 Moscow, Russia; 3 Department of Medical Nanobiotechnoilogy, Pirogov Russian National Research Medical University, Ostrovityanova Str. 1, 117997 Moscow, Russia

**Keywords:** styryl dyes, fluorescence probe, DNA binding, mitochondrial selectivity

## Abstract

New styryl dyes consisting of N-methylpyridine or N-methylquinoline scaffolds were synthesized, and their binding affinities for DNA in cell-free solution were studied. The replacement of heterocyclic residue from the pyridine to quinoline group as well as variation in the phenyl part strongly influenced their binding modes, binding affinities, and spectroscopic responses. Biological experiments showed the low toxicity of the obtained dyes and their applicability as selective dyes for mitochondria in living cells.

## 1. Introduction

Fluorescent dyes play a crucial role in various bioimaging applications, offering valuable insights into cellular processes and molecular interactions [1,2,3]. Among the diverse range of fluorescent dyes, styryl and cyanine dyes have garnered significant attention due to their structural tunability, favorable optical properties, and biocompatibility. These dyes have been widely explored as probes for visualizing cellular organelles and biomolecules, including nucleic acids [4,5,6].

Nucleic acids, such as DNA, are essential biomolecules that carry the genetic information of life. Their selective detection and imaging hold immense significance in molecular biology, medicine, and diagnostics. Small molecules capable of targeting nucleic acids have, therefore, attracted considerable scientific interest, offering potential implications in medicinal, biochemical, and biological studies. The noncovalent recognition of DNA by small molecules primarily relies on dominant binding modes, such as intercalation, groove binding, or electrostatic interactions with the sugar–phosphate backbone.

In recent years, there has been a growing focus on the development of fluorescent dyes with enhanced Stokes shift, which refers to the difference between the absorption and emission wavelengths. Dyes with larger Stokes shifts exhibit reduced background interferences and improved signal-to-noise ratios, making them highly desirable for fluorescence imaging. Excited-state intramolecular proton transfer (ESIPT) and intramolecular charge transfer (ICT) are two prominent photophysical pathways that have been extensively utilized to design fluorescent dyes with significant Stokes shifts.

In the literature, there are more and more works on the study of styryl dyes as fluorescent probes for biomolecules and organelles (e.g., mitochondria and lysosomes) [7,8,9,10,11]. Their structural tunability and excellent photophysical properties have made them highly suitable for fluorescence-based studies. Styryl dyes exhibit a unique “light-switch” behavior, remaining nonfluorescent until they interact with biomolecules (such us DNA), resulting in a strong fluorescence signal [12,13,14]. This property, combined with their high affinity for nucleic acids, provides an advantage in detecting and visualizing specific DNA sequences without background interference.

**Scheme 1:**
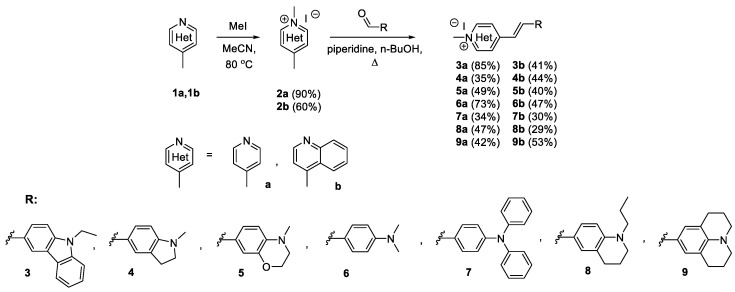
Synthesis of compounds **3a,b-9a,b**.

In this work, we designed a novel generation method of styryl dyes (Scheme 1), based on the variation of two distinct structural features: (a) in the heterocyclic part, we varied N-methylpyridine to N-methylquinoline (among the studied dyes, **6a** is a well-known commercially available DASPI); (b) we extended the aryl-amine moiety for shifting the absorption and emission bands to the longer-wavelength region. Thus, the prepared series of styryl dyes provides an analysis of how the composition of dye effects the interaction with calf thymus DNA (ct-DNA) and specific localization in cells.

## 2. Materials and Methods

### 2.1. Materials

Experimental details concerning the syntheses of compounds **2a-b**, **3a-9b** are presented in the Supplementary Materials.

### 2.2. Optical Spectroscopy

The absorption spectra were taken on a Cary 300 spectrophotometer (Agilent Technologies, Santa Clara, CA, USA). The fluorescence spectra were taken on a Cary Eclipse spectrofluorimeter (Agilent Technologies). The fluorescence quantum yields were determined using Rhodamine 6G in ethanol (the fluorescence quantum yield is 0.95) [15] and Coumarin 6 in ethanol (the fluorescence quantum yield is 0.78) [16] as the reference.

### 2.3. Preparation of DNA Solutions

Calf thymus DNA (Type I; highly polymerized sodium salt, Sigma-Aldrich, St. Louis, MO, USA) was dissolved in a 10 mM phosphate buffer (BPE) solution at a concentration of 1–2 mg/mL and stored at 4 °C for at least 16 h. Subsequently, the solution was passed through a PVDF membrane filter with a pore size of 0.45 μm to eliminate any insoluble materials. The concentrations of the DNA samples were determined by measuring the absorbance of the diluted stock solution (1:20) using the molar absorption coefficient ε_260_ = 12,824 cm^−1^ M^−1^ (in base pairs, bp).

The complex formation of **3a**-**9b** with ct-DNA was studied in aqueous solution using spectrophotometric and fluorimetric titration [17,18].

### 2.4. Circular Dichroism

The circular dichroism spectra were recorded using a SKD2MUF automatic recording dichrograph at a scanning speed of 20 nm/min and standard sensitivity across various wavelength ranges. All measurements of the solutions under study were performed in standard quartz cells with a path length of 10 mm at a temperature of 20 °C.

### 2.5. Thermal DNA Denaturation Studies

The melting point of DNA was determined in phosphate buffer at pH = 7 and a ratio of DNA–dye of 1:1 (*C*_Dye_ = 2.5·10^−5^; *C*_DNA_ = 2.5·10^−5^) and 2:1 (*C*_Dye_ = 2.5·10^−5^; *C*_DNA_= 5·10^−5^). DNA melting curves were recorded on an Avantes AvaSpec-ULS2048CLEVO-RS equipped with a Peltier cell. The samples were subjected to heating from 20.0 to 97.0 °C at a rate of 0.5 °C/min. The absorbance was continuously monitored at 260 nm as a function of temperature.

The normalized melting curves were then plotted in terms of absorbance change (*Â*), which was plotted against temperature, according to Equation (1):

(1) $$\hat{A}=\frac{A_{T}-A_{40\;{}^{\circ}C}}{A_{max}-A_{40\;{}^{\circ}C}}$$

where *A_T_* represents the absorbance at 260 nm at a specific temperature, *A_40 °C_* refers to the absorbance at 40 °C, and *A_max_* denotes the maximum absorbance within the temperature range of interest (40–90 °C). Ligand-induced changes in the DNA melting temperature (∆*T*_m_) were calculated according to Equation (2) and plotted against the ratio of ligand to DNA, LDR = *C*_Dye_/*C*_DNA_ [19].

(2) $$\Delta T_{m}=T_{m}\left(DNA-Ligand\right)-T_{m}\left(DNA\right)$$

### 2.6. Calculation of the Physicochemical Parameters Log P and W/L

Log P was determined using the ChemBioDraw Ultra 11.0 software package, which used specific algorithms for calculating log P and molar refractivity from fragment-based methods developed by the Medicinal Chemistry Project and BioByte. The three-dimensional structure of **3a,b**-**9a,b** was built with the MOPAC 2016 program package using the PM7 semiempirical method [20] to determine the W/L ratio.

### 2.7. Cytotoxicity Assay

The human cervical cancer cell line HeLa (ATCC, Manassas, VA, USA) was cultured in Dulbecco’s modified Eagle’s medium (DMEM) (Gibco, Billings, MT, USA) supplemented with 10% fetal bovine serum (Gibco, Billings, MT, USA) and 2 mM L-Glutamine (Gibco, USA). The cells were maintained in a humidified incubator (MCO-18AC, Sanyo, Osaka, Japan) at 37 °C with 5% CO_2_. When the cells reached approximately 80% confluence, they were harvested using TrypLE (Gibco, USA) and subcultured at a 1:8 ratio. Cell cultures were tested for the absence of mycoplasma.

For cytotoxicity analysis, HeLa cells were seeded into 96-well plates in 100 µL of growth medium (1 × 10^4^ cells/well). After 24 h of culturing, the growth medium was removed and replaced with substances in dimethyl sulfoxide (DMSO) solutions (with a final concentration of DMSO of 0.5% or less) at a concentration range of 0.125–4 µM and incubated for 48 h. Then, the growth medium was changed: 20 µL of MTS solution (Promega, Madison, WI, USA) was added to 100 µL of cell culture medium into each well. Then, the cells were incubated with MTS reagent for 4 h and the optical density was measured using a Multiscan GO plate reader (Thermo Scientific, Waltham, MA, USA), λ = 490 nm. All tests were performed in triplicate. All data are displayed as mean ± SD of three replicates.

### 2.8. Confocal Microscopy

For confocal microscopy, 2.5 × 10^5^ HeLa cells were seeded into a confocal Petri dish (SPL Life Science). The test dyes and Rhodamine 123 (Lumiprobe, Russia) were dissolved in DMSO to final concentrations of 50 µM and 50 mg/mL, respectively. After 24 h of culturing, the growth medium was removed and replaced with test dyes and Rhodamine 123 in dimethyl sulfoxide (DMSO) solutions (with a final concentration of DMSO of less 1%) at a concentration of 1 µM each and incubated for 30 min. Then, the cells were washed Dulbecco’s modified phosphate-buffered saline. Cell imaging was performed using a Nikon Eclipse Ti2 microscope (Nikon, Tokyo, Japan), Apo 25X/1,1 water immersion objective lens (Nikon, Tokyo, Japan). ImageJ2 Fiji (https://imagej.nih.gov/ij/, accessed on 19 May 2023) was used to process the images.

## 3. Results and Discussion

### 3.1. Synthesis

Styryl derivatives of **3a,b**-**9a,b** were obtained using a two-step synthesis. To begin, γ-picoline **1a** and 4-methylquinoline **1b** reacted with MeI via the Menshutkin reaction according to the described method [21]. The obtained heterocyclic salts **2a,b** interacted with the corresponding aldehydes via Knoevenagel condensation using n-butanol as a solvent and piperidine as a base (Scheme 1). The resulting styryl dyes **3a,b**-**9a,b** were identified and characterized with NMR spectroscopy, mass spectrometry, and elemental analysis. The description of synthetic procedures as well as structural characteristics is presented in the Electronic Supplementary Information (p. S2-S39 in ESI).

### 3.2. The Optical Properties of Free Dyes 3a-9b

**Figure 1:**
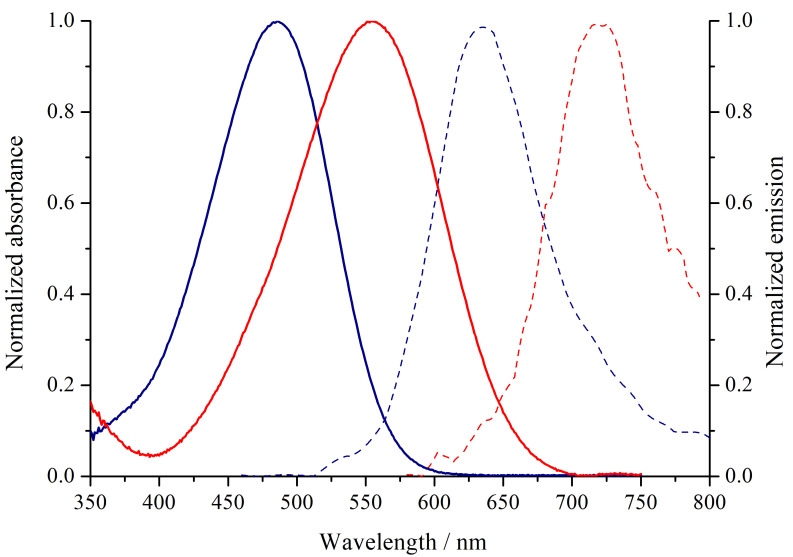
Normalized absorption (solid lines) and emission (dotted lines) spectra of **8a**—blue; **8b**—red. *C***_8a,8b_**= 10^−5^ mol·L^−1^, BPE buffer at pH = 7; λ_ex_ = 450 nm for dye **8a**, λ_ex_ = 570 nm for dye **8b**.

The optical characteristics of dyes **3a,b**-**9a,b** were studied in a phosphate buffer at pH = 7 (Table 1, Figure 1 and Figure S2 in ESI).

The absorption spectra of dyes **3a,b**-**9a,b** in the buffer solution show broad bands in the region from 400 to 700 nm, which correspond to intramolecular charge transfer from the donor nitrogen atom of the aryl fragment to the electron acceptor heterocyclic part. In addition, the absorption maxima of quinolinium derivatives **3b**-**9b** were shifted up to 40–70 nm to the redder region relative to the pyridinium analogues **3a**-**9a**, which is expected when the conjugated chain is elongated (Table 1, Figure 1 and Figure S2 in ESI). The absorption maxima of the dyes shifted bathochromically in the pyridinium series from **3a** to **9a**, and in the quinolinium series from **3b** to **9b** (Table 1, Figure 1 and Figure S2 in ESI). Such changes are associated with an increase in donor strength due to the planarity of the molecules and greater electron pair participation in conjugation.

**Figure 2:**
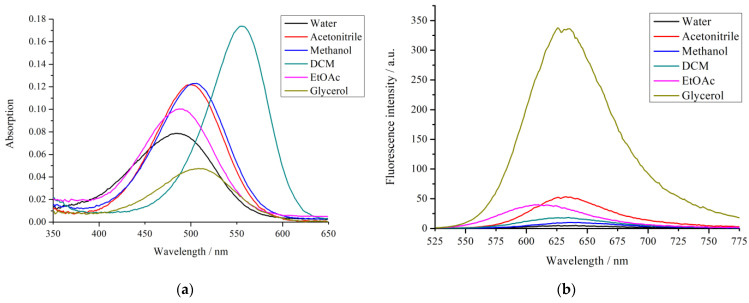
Absorption (**a**) and fluorescence (**b**) spectra of **8a** in different solvents; *C***_8a_** = 5∙10^−6^ mol·L^−1^; λ_ex_ = 510 nm.

When developing reagents for biolabeling, it is important to identify the dependence of optical properties on the nature of the solvent. The position of the bands in the absorption spectra seems to be determined by two factors: the polarity of the solvent and its ability to form hydrogen bonds [22]. The behavior of structurally similar styryl dyes in different solvents is known from the literature [9,23,24]. Such zwitterionic structures are characterized by the phenomenon of negative solvatochromism, when the ground state is more stabilized than the excited one. Thus, when studying the optical characteristics of **3a,b**-**9a,b** in various solvents, we observed a blue shift in the absorption maxima in more polar media (Figure 2, Tables S1 and S2 in ESI). The exception was dichloromethane (Table 2). This is probably due to the strong polarizability of the solvent.

The emission maxima of the obtained dyes were in the region of 500–650 nm for **3a**-**9a** and 650–750 nm for **3b**-**9b**, which is also consistent with an increase in the conjugation chain (Table 1, Figure 1). All derivatives had a rather low quantum yield of fluorescence, which can be explained by the parallel competing process of the formation of twisted TICT states [25,26]. This was confirmed by a strong fluorescence enhancement in 80% glycerol solution. When the viscosity of the medium increased (1 → 60.1 cP), rotation around the sigma bonds was hindered and the radiative relaxation increased (Table 2 and Table S3 in ESI).

In addition, for dyes **3a,b**-**9a,b**, the fluorescence response in aprotic solvents was higher than in aqueous medium (Table 2, Table S1 and Table S2 in ESI). Thus, they can be promising markers for cellular staining without washing steps [10].

All **3a,b**-**9a,b** derivatives had a large Stokes shift (Table 1) between 147 and 242 nm. Dyes with a large Stokes shift in bioimaging increase the signal-to-noise ratio and minimize the self-noise effect [27].

### 3.3. Interaction of Dyes 3a,b-9a,b with ct-DNA in Cell-Free Solution

Changes in the optical characteristics of dyes **3a,b**-**9a,b** upon interaction with double-stranded calf thymus DNA were studied (Table 3, Figures S3–S16 in ESI). Measurements were performed in aqueous phosphate buffer. The ratio between the dye and DNA was adjusted by titrating the dyes (*C***_3a,b-9a,b_** = 10^−5^ mol·L^−1^) with DNA solution.

**Figure 3:**
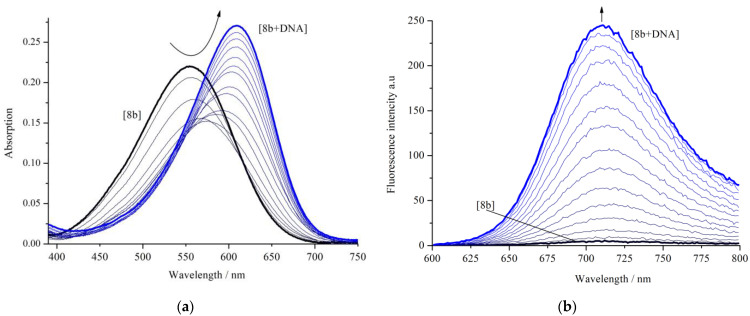
Spectrophotometric (**a**) and fluorimetric (**b**) titration of dye **8b** with ct-DNA solution; pH = 7, *C***_8b_** = 1∙10^−5^M, *C*_DNA_ = 0–0.7∙10^−3^ M b.p.

Increasing the DNA concentration led to changes in the dyes’ absorption spectra. After the first additions of DNA, a gradual decrease in optical density was observed; a further increase in DNA concentration in the solutions led to a red shift and increase in the absorption bands’ intensity (Table 3, Figure 3 and Figures S3a–S16a in ESI).

The bathochromic shift in the dye absorption band upon binding to DNA can be explained by a change in the local polarity around the dyes in the complex with DNA. The polarity of the dye environment decreases upon complexation with DNA, while the energy gap between the highest occupied molecular orbital (HOMO) and the lowest unoccupied molecular orbital (LUMO) of the dyes decreases, which is reflected as a bathochromic shift [28].

When titrating the dyes with ds-DNA solution, the appearance of an isosbestic point was not observed, which indicates the formation of complexes of several different types [14]. In addition, the formation of broad bands of complex shape (Figures S5a and S10a–S12a in ESI) was observed in the absorption spectra of dyes **3b**, **5a,b**, and **6b** when ds-DNA was added, which also confirms the hypothesis of the possible formation of several types of complexes or aggregates with DNA. Thus, it was not possible to determine stability constants from the spectrophotometric titration data.

As the DNA concentration increased, the dye emission maxima either shifted hypsochromically or did not change at all, which was also related to the influence of the dye environment on the relaxation mode (Table 3, Figures S3b–S16b in ESI).

Increasing the DNA concentration significantly enhanced the fluorescence response of the dye (Table 3, Figures S3b–S16b in ESI). Thus, the quantum yield of free **8b** was 0.07% and reached 4.45% at a concentration of 0.7∙10^−3^ M b.p. DNA in solution (Figure 3). Note that for the majority of the derivatives obtained, the fold of the fluorescent enhancement upon binding with ct-DNA was higher than that for commercially available DAPI and ethidium bromide [29].

To study changes in the properties of polynucleotides caused by the binding of small molecules, we chose CD spectroscopy as a highly sensitive method to conformational changes in the secondary structure of polynucleotides [30]. Additionally, to confirm the binding mode of small molecules with DNA, the difference in melting temperatures between free DNA and its complex with small molecules was used (ΔT_m_) [19,31].

The CD spectrum of free DNA has a positive band at about 280 nm and a negative band at about 245 nm, which are responsible for stacking-base-pair interactions and helix twisting, respectively [32]. These bands are quite sensitive to the interaction of DNA with small molecules. Furthermore, achiral small molecules have the potential to develop induced circular dichroism (ICD) spectra when they bind to polynucleotides. These ICD spectra can provide valuable insights into the modes of interaction between the small molecules and DNA.

**Figure 4:**
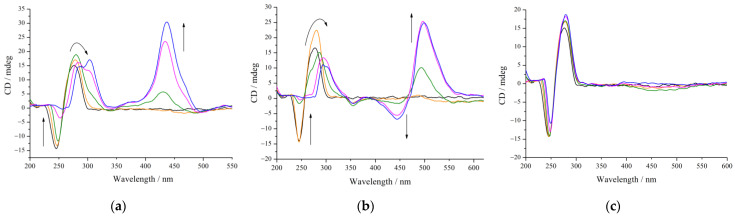
Circular dichroism spectra of ct-DNA (*C*_DNA_ = 0.1 mM b.p.) in the absence and presence of styryl dyes **3a** (**a**), **3b** (**b**), and **4a** (**c**) at different LDRs *C*_Dye_/*C*_DNA_: 0 (black); 0.1 (orange); 0.3 (green); 0.6 (magenta); 1 (blue).

The dyes **4a**-**9a** had little effect on the CD bands of DNA (230–290 nm), pointing to a minor structural deformation of the double stranded helix (Figure 4c, Figures S17a–S19a, S21a and S22a in ESI). The presence of dyes **3a,b, 7a** caused a hypochromic effect of the positive band as well as a shift toward a longer wavelength (Figure 4a and Figure S20a in ESI), which indicates the effect of dye binding on the folding of nitrogenous bases in the DNA double helix. The negative band of CD DNA changed more strongly with the addition of dyes **3b**-**9b** than with the addition of pyridinium analogues (Figure 4b and Figures S17–S22 in ESI).

It should be noted that the studied compounds **4a-6a** and **8a-9a** did not possess intrinsic ICD spectra (Figure 4c, Figures S17a–S19a, S20b, S21a and S22a in ESI). We also checked how the formation of complexes with these molecules affected the melting point of DNA (Table 4) and found that the addition of compounds **4a**-**9a** and **7b** did not change the melting point. However, the substantial changes in the optical spectra of these dyes point to the interaction of the dyes with DNA. Therefore, for dyes **4a-6a** and **8a-9a**, one can assume such a binding mode as external interaction along the polynucleotide backbone.

For DNA complexes with compound **3a**, we found the appearance of a positive induced CD (ICD) signal in the absorption region of the ligand (Figure 4a). The emergence of strong ICD bands is attributed to groove binding [33]. A weak increase in the melting temperature of the complex of **3a** with DNA (ΔT_m_ = 0–5) was found (Table 4), which also supports the groove binding mode.

For the complexes with **7a**,**3b**-**9b**, the ICD bands had a more complex, excitonic shape (Figure 4b, Figures S19a and S17b–S22b in ESI). The formation of excitonic bands usually correlates with the formation of chiral aggregates on the surface of a DNA chain [34,35], but external aggregation along the polynucleotide backbone cannot be excluded.

Dyes **4b-6b** and **8b** had little effect on the DNA melting point (ΔTm = 3–5 °C). However, the addition of dyes **3b** and **9b** led to a significant stabilization of the duplex (*C*_DNA_:*C*_Dye_ = 1:1, ΔT_m_ = 9 °C and ΔT_m_ = 10 °C, respectively) (Table 4). Quinolinium derivatives are expected to have a greater effect on the polynucleotide compared to their pyridinium analogues (Table 4). An increase in ΔT_m_ by more than 8 °C may indicate the stabilization of the DNA double helix and an interaction with the biomolecule by means of intercalation or groove binding [36].

The ambiguity of the obtained experimental results may indicate a mixed type of interaction of the dye with DNA, or interactions of different types at high and low concentrations of the dye [37]. The geometry of the planar aromatic systems of dyes is expected to influence the mechanism of DNA binding. It was anticipated that dyes whose chromophores approximated rectangles would tend to intercalate, while more rod-like chromophores would tend toward groove binding [38]. The method was suggested to be based on a quantitative assessment of the aspect (width to length) ratio of the dyes. The procedures were validated using a set of 38 cationic dyes of varied chemical structures binding to well-oriented DNA and demonstrated good agreement with experimental data. To identify correlations between the structure of the dye and the type of interaction with DNA, the ratios of the width to the length of the molecule (W/L) were calculated and are presented in Table 5. The W/L ratio for dyes **3b**–**9b** was greater than for dyes **3a**–**9a**, which is consistent with a decrease in the intensity of the negative DNA signal and the appearance of ICD signals in the CD spectra. Such effects may be a consequence of the influence of a wide quinoline fragment on the DNA structure. In the series of dyes **3a**–**9a**, the W/L ratio was higher for dyes **3a** and **7a**, which was also reflected in a decrease in intensity and a bathochromic shift in the positive DNA signal in the CD spectra. The reason for these changes may be the effect of the larger steric donor fragment of dyes **3a** and **7a** on the DNA structure.

### 3.4. Intracellular Localization of Dyes in Living Cells

Mitochondria are widely recognized as key targets for fluorescent bioimaging due to their involvement in various cellular processes and dysfunctions [39].

The study of the interaction of probes with living cells showed that cationic compounds with a log P value from 0 to 5 tend to accumulate in mitochondria [40]. We calculated log P for each of the compounds **3a,b-9a,b**. Most of the dyes fell within the required range, thus demonstrating their lipophilic character (Table 5).

For structures **4-6a** and **9a,** they had a log P < 0, which indicates a greater hydrophilicity of the molecule (Table 5). The value −4 < log P < 0 is usually compared with nuclear localization, but in this case, it is worth evaluating the parameter of binding to the nucleic acid LCF (largest conjugated fragment; proportional to the area of the coplanar aromatic region) [40]. For nuclear derivatives, the value of this parameter is LCF ώ 17, while for structures **4-6a** and **9a,** its value was only 16. Thus, for **4-6a** and **9a**, we can also expect accumulation in mitochondria due to the dyes’ structures, as well as the high value of the mitochondrial membrane potential (gradient of −140 to −180 mV).

**Figure 5:**
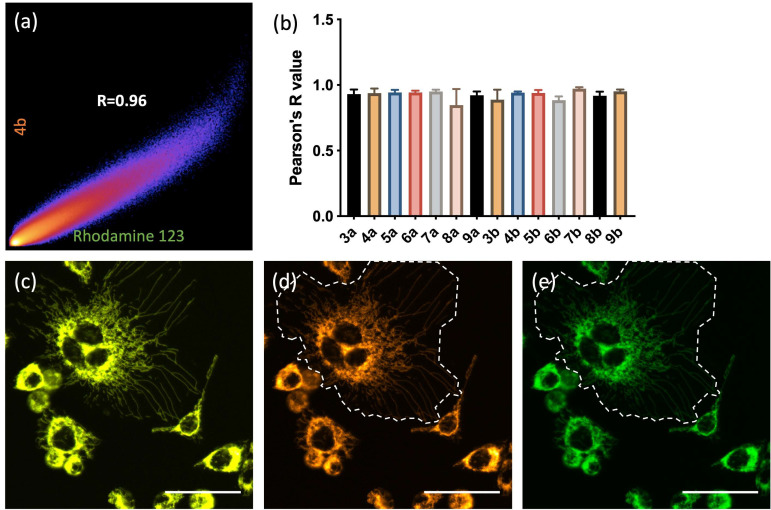
(**a**) Fluorescence intensity correlation plot of d and e; (**b**) diagrams of Pearson’s R values of dyes **3a**-**9b** (n = 5–7) for live HeLa cells; (**c**) merged image of d and e channels; (**d**) fluorescence confocal image of HeLa live cells stained with dye **4b** (1 μM) (λ_ex_ = 488 nm, range detection 575–625 nm); (**e**) fluorescence confocal image of HeLa live cells stained with Rhodamine 123 (250 μg/mL) (λ_ex_ = 488 nm, range detection 500–545 nm). The white dashed line is indicate ROI for Pearson’s R value calculation; laser scanning confocal microscopy; scale bar 50 µm.

HeLa cells were used to study the localization of the dye in living systems. The toxicity of the obtained dyes was preliminarily assessed for an incubation time of 48 h. According to the obtained data, all dyes at a concentration of 1 μM or less were nontoxic for these cells (Figure S23 in ESI) and can be used for cell staining. To show the dyes’ colocalization with mitochondria, additional staining was performed with Rhodamine 123, which is often used to label mitochondria in cells. Pearson’s colocalization coefficients R were determined for all types of dyes using manual ROI selection. In particular, for dye **4b**, the Pearson coefficient was 0.96 (Figure 5a–e) The imaging results demonstrate that all probes were capable of imaging the mitochondria in HeLa cells with high selectivity (Figures S24 and S25 in ESI).

## 4. Conclusions

A series of 14 monocationic styryl dyes were prepared in moderate yields via Knoevenagel condensation based on N-methyl-substituted pyridinium and quinolinium. The structural elucidation of the newly synthesized fluorophores was achieved with ^1^H NMR, ^13^C NMR, mass spectrometry in the ESI mode, and elemental analysis. Novel dyes were characterized by very pronounced Stokes shifts (147–242 nm), while the fluorescence quantum yields (0.05–1.68%) were low. The fluorescence intensity increased in aprotic and viscous environments.

Thus, dyes are sensitive to the environment, so how they interacted with DNA and changed their fluorescence in a cell was studied. The combined results of several methods indicate a possible mixed type of interaction with DNA, which is consistent with the literature data for similar structures [37].

The heterocyclic part of the dyes affected the position of the absorption and fluorescence bands and the mode of binding to DNA, while the effect of the styryl part on complex formation with DNA was negligible.

All dyes investigated in this work showed negligible cytotoxic activity. All of the studied dyes exhibited localization within mitochondria. The excitation and emission wavelengths of prepared dyes were close to the near-infrared window, which minimizes the effect of cellular autofluorescence.

The observed bioactivity of the dyes, combined with easily monitored localization using fluorescence, makes these dyes potential agents for studying the biophysical properties of membranes [41,42], protein properties [24,43], and DNA [13,44].

## Figures and Tables

**Table 1:** Spectral characteristics of dyes **3a,b-9a,b** in BPE buffer at pH = 7, C**_3a,b-9a,b_** = 10^−5^ mol·L^−1^.

Compound	$\lambda_{max}^{abs}/\mathrm{nm}\;$	ε_λ_/l·mol^−1^·cm^−1^	$\lambda_{max}^{fl}/\mathrm{nm}$	$\tilde{\nu }/\mathrm{nm}$	$\tilde{\nu }/$cm^−1^	*ϕ^fl^/*%
**3a**	419	29,647	595	176	7060	1.68
**4a**	430	28,160	637	207	7557	0.16
**5a**	446	25,601	638	192	6748	0.16
**6a** (DASPI)	449	28,818	616	167	6038	0.22
**7a**	451	21,202	650	199	6788	0.06
**8a**	485	33,179	632	147	4796	0.23
**9a**	491	21,724	644	153	4839	0.14
**3b**	459	23,061	658	199	6589	0.32
**4b**	484	16,155	726	242	6887	0.03
**5b**	505	10,055	718	213	5874	0.05
**6b**	510	21,515	694	184	5199	0.05
**7b**	494	6371	728	234	6507	0.04
**8b**	554	22,022	713	159	4025	0.07
**9b**	568	18,707	730	162	3907	0.05

**Table 2:** Fluorescence quantum yields (%) of dyes **3a-9b** in various solvents.

**Solvents**	**3a**	**4a**	**5a**	**6a (DASPI)**	**7a**	**8a**	**9a**
Water	1.68	0.16	0.16	0.22	0.06	0.23	0.14
Methanol	22.6	0.18	0.25	0.63	0.29	0.48	0.3
Acetonitrile	25	0.09	0.15	0.36	0.16	0.28	0.22
EtOAc	6.95	0.18	0.72	1.02	1.13	1.41	0.97
DCM	22.3	1.89	0.98	8.78	2.21	1.8	0.63
Glycerol	42.1	15.2	5.82	13.4	4.61	18.4	5.54
**Solvents**	**3b**	**4b**	**5b**	**6b**	**7b**	**8b**	**9b**
Water	0.12	0.03	0.05	0.05	0.04	0.07	0.05
Methanol	6.44	0.09	0.13	0.18	*	0.19	0.22
Acetonitrile	6.39	*	*	0.09	*	0.11	0.18
EtOAc	3.74	0.25	0.21	0.18	*	0.49	0.74
DCM	39.5	0.28	0.44	1.89	*	0.55	1.52
Glycerol	15.2	0.89	1.12	15.2	1.7	1.9	1.28

* fluorescence quantum yield < 0.001%

**Table 3:** Spectral characteristics of **3a,b**-**9a,b** dye complexes with ct-DNA in BPE buffer at pH = 7, *C***_3a,b-9a,b_** = 10^−5^ mol·L^−1^.

Compound	λ^abs^ nm (Δλ nm) *	λ^fl^ nm (Δ λ nm) *	*ϕ^fl^* (%)	φwith DNAflφfree dyefl
**3a**	455 (36)	583 (12)	26.70	16
**4a**	506 (76)	638 (1)	10.00	64
**5a**	485 (39)	630 (8)	9.50	61
**6a** (DASPI)	490 (41)	616 (0)	19.80	89
**7a**	475 (24)	633 (17)	3.74	60
**8a**	514 (29)	628 (4)	11.40	48
**9a**	532 (41)	642 (2)	10.40	74
**3b**	500 (41)	622 (36)	15.32	48
**4b**	562 (78)	720 (6)	0.97	31
**5b**	565 (60)	714 (4)	1.30	27
**6b**	573 (63)	689 (5)	4.91	90
**7b**	537 (43)	693 (35)	0.40	10
**8b**	607 (53)	709 (4)	4.45	66
**9b**	625 (57)	728 (2)	1.59	30

* Δλ = │ λ_free ligand_ − λ_bound ligand_│, nm.

**Table 4:** ΔT_m_ (°C) values of ct-DNA for different ratios *C*_DNA_/*C*_Dye_; pH = 7.

Compound	Ratio		Compound	Ratio	
	*C*_DNA_:*C*_Dye_ = 1:1	*C*_DNA_:*C*_Dye_ = 2:1		*C*_DNA_:*C*_Dye_ = 1:1	*C*_DNA_:*C*_Dye_ = 2:1
**3a**	4	4	**3b**	9	7
**4a**	1	1	**4b**	5	4
**5a**	0	0	**5b**	2	2
**6a** (DASPI)	0	0	**6b**	4	3
**7a**	0	1	**7b**	0	0
**8a**	1	0	**8b**	3	3
**9a**	0	0	**9b**	10	9

**Table 5:** Physicochemical parameters of dyes **3a,b**-**9a,b**.

Dye	Log P *	Size (Bond Number)			Size (Å)			Dye	Log P*	Size (Bond Number)			Size (Å)		
		W	L	W/L	W	L	W/L			W	L	W/L	W	L	W/L
**3a**	0.86	3	10	0.3	2.85	10.7	0.27	**3b**	2.25	4	10	0.4	4.80	10.6	0.45
**4a**	−1.09	2	10	0.2	2.45	10.6	0.23	**4b**	0.30	4	10	0.4	4.80	10.6	0.45
**5a**	−1.28	2	10	0.2	2.42	10.7	0.23	**5b**	0.10	4	10	0.4	4.80	10.7	0.45
**6a**	−1.34	2	10	0.2	2.41	10.7	0.22	**6b**	0.04	4	10	0.4	4.81	10.7	0.45
**7a**	2.09	6	10	0.6	7.15	10.7	0.67	**7b**	3.47	6	10	0.6	7.16	10.7	0.67
**8a**	0.38	2	10	0.2	2.44	10.7	0.23	**8b**	1.76	4	10	0.4	4.80	10.7	0.45
**9a**	−0.25	2	10	0.2	2.37	10.7	0.22	**9b**	1.13	4	10	0.4	4.80	10.7	0.45

*—Log P is the logarithm of the octanol–water partition coefficient.

## Data Availability

Not applicable.

## Supplementary Materials

The references in Supplementary Materials were cited in Refs. [21,45,46,47,48,49,50,51,52,53,54]. The following supporting information can be downloaded at: https://www.mdpi.com/article/10.3390/bios13070734/s1, Figure S1: Synthetic scheme; S2-S7 Synthetic procedures and characterization data for compounds **2a,b**-**9a,b**; S8-S36 ^1^H and ^13^C, 2D NMR spectra of compounds **2a,b**-**9a,b**; S37-S39 ESI MS studies for **3-5a**, **7-9a**, **3-5b**, **8b**, **9b**; Figure S2: Absorption (a,c) and fluorescence (b,d) spectra of **3a,b-9a,b** in BPE buffer at pH = 7, C**_3a-9b_** = 1∙10^−5^; Figure S3–S16: Spectrophotometric (a) and fluorometric (b) titration of dyes **3a,b-9a,b** with ds-DNA solution, pH = 7; Figure S17–S22: Circular dichroism spectra of ct-DNA (C_DNA_ = 0.1 mM b.p.) in the absence and presence of styryl dyes **4-9a** (a) and **4-9b** (b) at different LDR C_Dye_/C_DNA_: 0 (black), 0.1 (orange), 0.3 (green), 0.6 (magenta), 1 (blue); Figure S23: Cell survival of HeLa cells treated with different concentrations of **3-9a** and **3-9b** dyes (a-d); Figure S24: Fluorescence confocal images of HeLa live cells. Orange hot channel is corresponding **3-9a** dyes (1 μM) (λ_ex_ = 488 nm, detection 575–625 nm); green channel is corresponding Rhodamine 123 (250 μg/mL) (λ_ex_ = 488 nm, detection 500–545 nm). Laser scanning confocal microscopy, scale bar 50 µm; Figure S25: Fluorescence confocal images of HeLa live cells. Orange hot channel is corresponding **3-9b** dyes (1 μM) (λ_ex_ = 488 nm, detection 575–625 nm); green channel is corresponding Rhodamine 123 (250 μg/mL) (λ_ex_ = 488 nm, detection 500–545 nm). Laser scanning confocal microscopy, scale bar 50 µm; Table S1: Optical characteristics of dyes **3-9a** in different solvents; Table S2: Optical characteristics of dyes **3-9b** in different solvents; Table S3: Optical characteristics of dyes **3-9b** in glycerol.
