# Supplementary material for: Specific Fluorescent Probes for Imaging DNA in Cell-Free Solution and in Mitochondria in Living Cells
Source: Biosensors (Basel). 2023 Jul 15;13(7):734. doi: 10.3390/bios13070734 (PMC10377282; doi:10.3390/bios13070734)
Supplement: Supplementary file 1 [file biosensors-13-00734-s001.zip › biosensors-2454932-supplementary.pdf]

Electronic Supplementary Information

Specific Fluorescent Probes for Imaging DNA in Cell-Free Solution and in Mitochondria in Living Cells

Anna S. Efimova, Mariya A. Ustimova, Nelly S. Chmelyuk, Maxim A. Abakumov, Yury V. Fedorov, Olga A. Fedorova

CONTENTS

1.Synthesis of the compounds 2

2.^{1}H and ^{13}C, 2D NMR spectra of compounds 8

3. ESI MS studies 37

4. Optical studies 40

5. Cytotoxicity assays 50

6. Confocal microscopy 51

1. Synthesis of the compounds

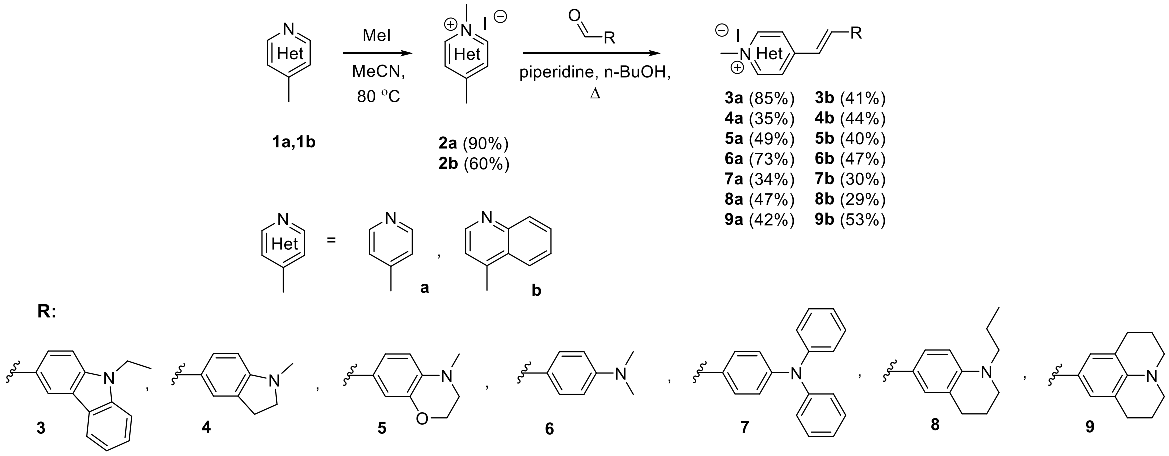

Figure S1. Synthetic scheme.

Apparatus

^{1}H and ^{13}C NMR spectra were recorded on Avance 400 (Bruker) and Inova 400 (Agilent) spectrometers operating at 400.13 MHz (for ^{1}H) and 100.60 MHz (for ^{13}C). The chemical shifts were determined with an accuracy of 0.01 ppm relative to residual solvent signals and translated to the internal standard (TMS), and coupling constants were measured with an accuracy of 0.1 Hz. The assignment of ^{1}H and ^{13}C signals was based on 2D NMR experiments (HMBC, HSQC, ^{1}H COSY, NOESY), which were performed using standard pulse sequences from the Bruker and Agilent library. The numbering of carbon atoms in the styryl fragments used for the description of the ^{1}H NMR spectra is shown on the corresponding structures next to the description of the synthesis.

Melting points were measured on melt-temp melting point electrothermal apparatus and were uncorrected. The reaction course and purity of the final products was followed by TLC on silica gel (DC-Alufolien Kieselgel 60 F 254, Sigma-Aldrich) and aluminum oxide (Aluminium oxide 60 F 254, neutral, Merck). Flash column chromatography was conducted over silica gel (Kieselgel, 40-60 µm, Acros Organics) using a preparative low pressure chromatograph Isolera Prime (Biotage). Preparative TLC was performed on silica gel 60 (Merck) using 20×20 cm plates with a layer thickness of 1 mm. LC-ESI-MS analyses were performed on a Shimadzu LCMS-2020, using methanol (Panreac, HPLC-gradient grade) as the mobile phase.

Elemental analyses were carried out in the Microanalysis Laboratory of the A.N. Nesmeyanov Institute of Organoelement Compounds. Starting compounds and reagents were obtained from commercial sources (Sigma Aldrich, Merck) and were used without any further purification.

Synthetic procedures and characterization data

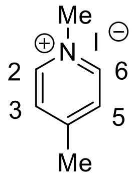

1,4-dimethylpyridin-1-ium iodide (1a)

1a was synthesized according to the literature [21]. Beige solid. Yield 90%. M.p. 146-148 ℃. ^{1}H NMR (400 MHz, DMSO-d_6, δ ppm., *J* Hz): 2.59 (s, 3H, -CH_3), 4.26 (s, 3H, N^+-CH_3), 7.95 (d, 2H, H-3,5, *J* = 6.3), 8.82 (d, 2H, H-2,6, *J* = 6.3) [45].

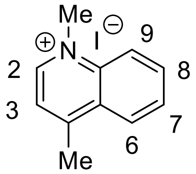

1,4-dimethylquinolin-1-ium iodide (1b)

1b was synthesized according to the literature [21]. Yellow powder. Yield 60%. M.p. 167-168 ℃. ^{1}H NMR (400 MHz, DMSO-d_6, δ ppm., J Hz): 3.00 (s, 3H, -CH_3), 4.57 (s, 3H, N^+-CH_3), 8.13 – 7.98 (m, 2H, H-3,7), 8.27 (t, 1H, H-8, J = 7.9), 8.49 (d, 1H, H-9, J = 8.9), 8.54 (d, 1H, H-6, J = 8.4), 9.35 (d, 1H, H-2, J = 6.0) [46].

General procedure for the synthesis of compounds 3a-9b

To a stirring mixture of **1a** or **1b** (1 eqv.), corresponding aldehyde (1 eqv.) and n-BuOH, the piperidine (0,2 eqv.), was added. The mixture was refluxed for 10 hours. Then, the solvent was removed in vacuum. Addition of methanol precipitated products **3a-9a**. The precipitates were filtered off and washed with hexane and diethyl ether. The products **3b-9b** were purified with flash column chromatography using CH_2Cl_2/methanol as the eluent.

(E)-4-(2-(9-ethyl-9H-carbazol-3-yl)vinyl)-1-methylpyridin-1-ium iodide (3a)

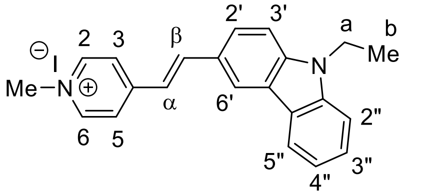

Yellow powder. Yield 85%. M.p. 290-292 ℃. ^{1}H NMR (400 MHz, DMSO-d_6, δ ppm., J Hz): 1.34 (t, 3H, H-b, J = 6.9), 4.23 (s, 3H, N^+-CH_3), 4.50 (q, 2H, H-a, J = 6.6), 7.29 (t, 1H, H-3”, J = 7.4), 7.53 (m, 2H, H-α,4”), 7.68 (d, 1H, H-2”, J = 8.2), 7.75 (d, 1H, H-3’, J = 8.5), 7.90 (d, 1H, H-2’, J = 8.6), 8.20 (m, 4H, H-β,3,5,5”), 8.59 (s, 1H, H-6’), 8.80 (d, 2H, H-2,6, J = 6.2). ^{13}C NMR (101 MHz, DMSO-d_6, δ ppm., J Hz): 13.80 (C-b), 37.25 (C-a), 46.66 (N^+-CH_3), 109.70 (C-2”), 109.84 (C-3’), 119.66 (C-4”), 119.89 (C-α), 120.53 (C-5”), 121.20 (C-6’), 122.12 (C-6”), 122.72 (C-3,5,5’), 126.22 (C-1'), 126.31 (C-2’), 126.44 (C-3”), 140.10 (C-1”), 140.90 (C-4’), 142.27 (C-β), 144.69 (C-2,6), 153.03 (C-4) [47]. ESI-MS **3a** in MeOH, m/z: calcd. 313.17; found 313.00 $[M]^+$. Anal. calcd. for $C_{22}H_{21}I^+N_2^+$: C, 60.01 %; H, 4.81 %; N, 6.36 %; found: C, 60.21%; H, 4.85%; N, 6.32%.

(E)-1-methyl-4-(2-(1-methylindolin-5-yl)vinyl)pyridin-1-ium iodide (4a)

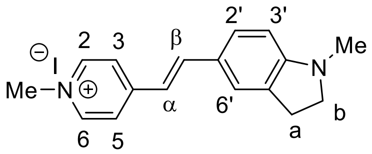

Red solid. Yield 35%. M.p. 209-211 ℃. ^{1}H NMR (400 MHz, DMSO-d_6, δ ppm., J Hz): 2.82 (s, 3H, N-CH_3), 2.98 (t, 2H, H-a, J = 8.2), 3.46 (t, 2H, H-b J = 8.2), 4.16 (s, 3H, N^+-CH_3), 6.53 (d, 1H, H-3’, J = 8.2), 7.13 (d, 1H, H-α, J_{trans} = 16.0), 7.39 (d, 1H, H-2’, J = 8.2 Hz), 7.49 (s, 1H, H-6’), 7.88 (d, 1H, H-β, J_{trans} = 16.0), 8.00 (d, 2H, H-3,5, J = 6.6), 8.66 (d, 2H, H-2,6, J = 6.6). ^{13}C NMR (101 MHz, DMSO-d_6, δ ppm., J Hz): 27.24 (C-a), 34.17 (N-CH_3), 46.30 (N^+-CH_3), 54.50 (C-b), 105.73 (C-3’), 116.58 (C-α), 121.95 (C-3,5), 123.21 (C-6’), 123.91 (C-1’), 130.92 (C-5’), 131.26 (C-2’), 142.27 (C-β), 144.25 (C-2,6), 153.41 (C-4), 155.49 (C-4’). ESI-MS **4a** in MeOH, m/z: calcd. 251.15; found 251.00 $[M]^+$. Anal. calcd. for $C_{17}H_{19}I^-N_2^+$: C, 53.98 %; H, 5.06 %; N, 7.41 %; found C, 52.58 %; H, 5.00 %; N, 7.03 %.

(E)-1-methyl-4-(2-(4-methyl-3,4-dihydro-2H-benzo[b][1,4]oxazin-7-yl)vinyl)pyridin-1-ium iodide (5a)

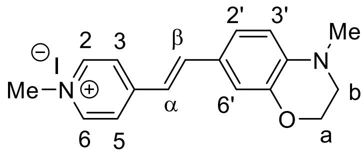

Red solid. Yield 49%. M.p. 253-254 ℃. ^{1}H NMR (400 MHz, DMSO-d_6, δ ppm., *J* Hz): 2.95 (s, 3H, N-CH_3), 3.38 (m, 2H, H-b), 4.17 (s, 3H, N^+-CH_3), 4.23 (m, 2H, H-a), 6.75 (d, 1H, H-3', *J* = 8.5), 7.16 (m, 3H, H- H-α,2',6'), 7.84 (d, 1H, H-β, J_{trans} = 16.0), 8.03 (d, 2H, H-3,5, *J* = 6.4), 8.70 (d, 2H, H-2,6, *J* = 6.4). ^{13}C NMR (101 MHz, DMSO-d_6, δ ppm., *J* Hz): 37.91 (N-CH_3), 46.50 (N^+-CH_3), 48.11 (C-b), 64.01 (C-a), 111.63 (C-3'), 113.95 (C-2'), 118.13 (C-α), 122.34 (C-3,5), 124.03 (C-1'), 124.33 (C-6'), 139.39 (C-4'), 141.66 (C-β), 143.56 (C-5'), 144.48 (C-2,6), 153.22 (C-4). ESI-MS **5a** in MeOH, m/z: calcd. 267.15; found 267.00 $[M]^+$. Anal. calcd. for $C_{17}H_{19}I^-N_2^+O$: C, 51.79 %; H, 4.86 %; N, 7.11 %; found C, 52.03 %; H, 4.95 %; N, 7.42 %.

***(E)-4-(4-(dimethylamino)styryl)-1-methylpyridin-1-ium iodide (6a,** DASPI)*

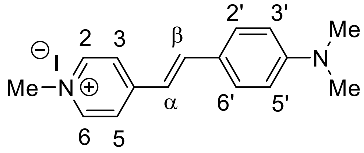

Crimson powder. Yield 73%. M.p. 252-254 ℃. ^{1}H NMR (400 MHz, DMSO-d_6, δ ppm., *J* Hz): 3.01 (s, 6H, N-$(CH_3)_2$), 4.18 (s, 3H, N^+-CH_3), 6.78 (d, 2H, H-3',5', *J* = 8.8), 7.17 (d, 1H, H-α, J_{trans} = 16.1), 7.60 (d, 2H, H-2',6', *J* = 8.8), 7.91 (d, 1H, H-β, *J* = 16.1), 8.05 (d, 2H, H-3,5, *J* = 6.8), 8.69 (d, 2H, H-2,6, *J* = 6.8). ^{13}C NMR (101 MHz, DMSO-d_6, δ ppm., *J* Hz): 39.52 (N-$(CH_3)_2$), 46.37 (N^+-CH_3), 111.93 (C-3',5'), 117.10 (C-α), 122.10 (C-3,5), 122.45 (C-1'), 130.12 (C-2',6'), 141.86 (C-β), 144.31 (C-2,6), 151.84 (C-4'), 153.34 (C-4) [48].

Anal. calcd. for $C_{16}H_{19}I^-N_2^+$: C, 52.47 %; H, 5.23 %; N, 7.65 %; found C, 52.05 %; H, 5.11 %; N, 7.34 %.

(E)-4-(4-(diphenylamino)styryl)-1-methylpyridin-1-ium iodide (7a)

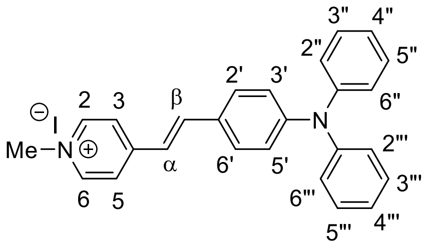

Crimson powder. Yield 34%. M.p. 286-288 ℃. ^{1}H NMR (400 MHz, DMSO-d_6, δ ppm., *J* Hz): 4.21 (s, 3H, N^+-CH_3), 6.95 (d, 2H, H-3',5', *J* = 8.6), 7.12 (d, 4H, H-2",6",2"',6"', *J* = 8.3), 7.17 (t, 2H, H-4",4"', *J* = 7.4), 7.30 (d, 1H, H-α, J_{trans} = 16.2), 7.38 (t, 4H, H-3",5",3"',5"', *J* = 7.7), 7.62 (d, 2H, H-2',6', *J* = 8.6), 7.94 (d, 1H, H-β, J_{trans} = 16.2), 8.14 (d, 2H, H-3,5, *J* = 6.5), 8.78 (d, 2H, H-2,6, *J* = 6.5). ^{13}C NMR (101 MHz, DMSO-d_6, δ ppm., *J* Hz): 46.69 (N^+-CH_3), 120.45 (C-α), 120.69 (C-3',5'), 122.89 (C-3,5), 124.48 (C-4",4"'), 125.38 (C-2",6",2"',6"'), 127.98 (C-1'), 129.66 (C-2',6'), 129.84 (C-3",5",3"',5"'), 140.47 (C-β), 144.77 (C-2,6), 146.19 (C-1",1"'), 149.42 (C-4'), 152.79 (C-4) [49]. ESI-MS **7a** in MeOH, m/z: calcd. 363.19; found 362.95 $[M]^+$. Anal. calcd. For $C_{26}H_{23}I^-N_2^+$: C, 63.68 %; H, 4.73 %; N, 5.71 %; found C, 63.73 %; H, 4.80 %; N, 5.73 %.

(E)-1-methyl-4-(2-(1-propyl-1,2,3,4-tetrahydroquinolin-6-yl)vin4yridinedin-1-ium iodide (8a)

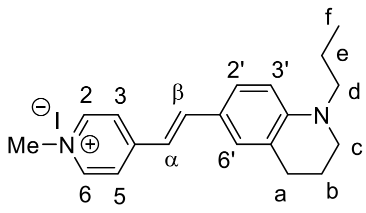

Crimson solid. Yield 47%. M.p. 210-212 ℃. ^{1}H NMR (400 MHz, DMSO-d_6, δ ppm., *J* Hz): 0.91 (t, 3H, H-f, *J* = 7.1), 1.87 (m, 2H, H-e), 2.72 (m, 2H, H-a), 3.29 (m, 2H, H-d), 3.36 (m, 2H, H-c), 4.15 (s, 3H, N^+-CH_3), 6.65 (d, 2H, '-3', *J* = 8.9), 7.07 (d, 1H, H-α, J_{trans}= 16.1), 7.35 (m, 2H, '-2',6'), 7.83 (d, 1H, H-β, J_{trans}= 16.1), 7.99 (d, 2H, H-3,5, *J* = 6.3), 8.63 (d, 2H, H-2,6, *J* = 6.3). ^{13}C NMR (101 MHz, DMSO-d_6, δ ppm., *J* Hz): 11.23 (C-f), 19.13 (C-e), 21.20 (C-b), 27.51 (C-a), 46.22

(N^+-CH_3), 49.00 (C-c), 52.08 (C-d), 110.20 (C-3’), 116.08 (C-α), 121.65 (C-1’), 121.79 (C-3,5), 121.92 (C-5’), 128.90 (C-2’), 129.39 (C-6’), 142.14 (C-β), 144.15 (C-2,6), 147.63 (C-4’), 153.42 (C-4). ESI-MS **8a** in MeOH, m/z: calcd. 293.20; found 293.015 $[M]^+$. Anal. calcd. for $C_{20}H_{25}I^- N_2^+$: C, 57.15 %; H, 6.00 %; N, 6.66 %; found C, 57.10 %; H, 6.04 %; N, 6.89 %.

(E)-1-methyl-4-(2-(2,3,6,7-tetrahydro-1H,5H-pyrido[3,2,1-ij]quinolin-9-yl)vinyl)pyridin-1-ium iodide (9a)

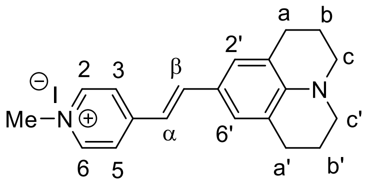

Crimson solid. Yield 42%. M.p. 233-235 ℃. ^{1}H NMR (300 MHz, DMSO-d_6, δ ppm., J Hz): 1.87 (m, 4H, H-b,b’), 2.70 (t, 4H, H-a,a’, J = 5.8), 3.26 (m, 4H, H-c,c’), 4.13 (s, 3H, N^+-CH_3), 7.04 (d, 1H, H-α, J_{trans} = 15.8), 7.15 (s, 2H, H-2’,6’), 7.78 (d, 1H, H-β, J_{trans} = 15.8), 7.95 (d, 2H, H-3,5, J = 6.7), (d, 2H, H-2,6, J = 6.6). ^{13}C NMR (101 MHz, DMSO-d_6, δ ppm., J Hz): 20.98 (C-b,b’), 27.14 (C-a,a’), 46.21(N^+-CH_3), 49.29 (C-c,c’), 115.85 (C-α), 120.74 (3’,5’), 121.38 (C-1’), 121.68 (C-3,5), 127.86 (C-2’,6’), 142.32 (C-β), 144.13 (C-2,6), 145.20 (C-4’), 153.41 (C-4) [50]. ESI-MS **9a** in MeOH, m/z: calcd. 291.19; found 291.00 $[M]^+$. Anal. calcd. for $C_{20}H_{23}I^- N_2^+$: C, 57.42 %; H, 5.54 %; N, 6.70 %; found C, 57.09 %; H, 5.50 %; N, 6.65 %.

(E)-4-(2-(9-ethyl-9H-carbazol-3-yl)vinyl)-1-methylquinolin-1-ium iodide (3b)

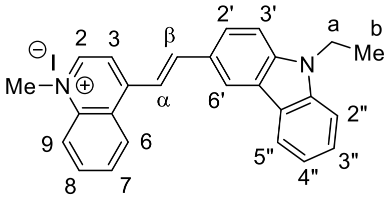

Red powder. Yield 41%. T.d. 289 ℃. ^{1}H NMR (400 MHz, DMSO-d_6, δ ppm., J Hz): 1.36 (t, 3H, H-b, J = 7.2), 4.50 (s, 5H, H-a, N^+-CH_3), 7.31 (t, 1H, H-4”, J = 7.4), 7.54 (t, 1H, H-3”, J = 7.6), 7.69 (d, 1H, H-2”, J = 8.1), 7.76 (d, 1H, H-3’, J = 8.5), 8.06 (t, 1H, H-7, J = 8.5), 8.13 (d, 1H, H-2’, J = 8.5), 8.26 (m, 2H, H-5”,8), 8.40 (m, 3H, H-β,9,α), 8.49 (d, 1H, H-3 J = 6.4), 8.86 (s, 1H, H-6’), 9.12 (1H, H-6, J = 8.5), 9.27 (d, 1H, H-2, J = 6.4). ^{13}C NMR (101 MHz, DMSO-d_6, δ ppm., J Hz): 13.86 (C-b), 37.32 (C-a), 44.44 (N^+-CH_3), 109.85 (C-2”,3’), 115.11 (C-3), 116.18 (C-α), 119.31 (C-9), 119.82 (C-4”), 120.62 (C-5”), 121.93 (C-6’), 122.29 (C-6”), 122.88 (C-5’), 126.12 (C-6), 26.48 (C-3”), 26.57 (C-5), 126.71 (C-1’), 127.57 (C-2’), 128.98 (C-7), 134.89 (C-8), 138.84 (C-10), 140.22 (C-1”), 141.28 (C-4’), 144.92 (C-β), 147.57 (C-2), 153.04 (C-4) [51]. ESI-MS **3b** in MeOH, m/z: calcd. 363.19; found 363.35 $[M]^+$. Anal. calcd. For $C_{26}H_{23}I^- N_2^+$: C, 63.68 %; H, 4.73 %; N, 5.71 %; found C, 63.75 %; H, 4.80 %; N, 5.75 %.

(E)-1-methyl-4-(2-(1-methylindolin-5-yl)vin5uinolinelin-1-ium iodide (4b)

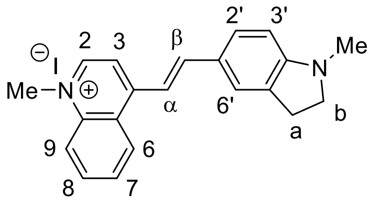

Dark solid. Yield 44%. M.p. 215-216 ℃. ^{1}H NMR (400 MHz, DMSO-d_6, δ ppm., J Hz): 2.88 (s, 3H, N-CH_3), 3.03 (t, 2H, H-a, J = 8.2), 3.54 (t, 2H, H-b, J = 8.2), 4.41 (s, 3H, N^+-CH_3), 6.57 (d, 1H, H-3’, J = 8.2), 7.59 (d, 1H, H-2’, J = 8.3), 7.85 (s, 1H, H-6’), 7.95 (m, 2H, H-α,7), 8.17 (m, 2H, H-β,8), 8.30 (m, 2H, H-3,9), 9.01 (d, 1H, H-6, J = 8.4), 9.06 (d, 1H, H-2, J = 6.6). ^{13}C NMR (101 MHz, DMSO-d_6, δ ppm., J Hz): 27.16 (C-a), 33.96 (N-CH_3), 43.96 (N^+-CH_3), 54.42 (C-b), 105.60 (C-3’), 112.33 (C-α), 113.57 (C-3), 118.98 (C-9), 124.20 (C-6’), 124.51 (C-1’), 125.73 (C-5), 126.30 (C-6), 128.48 (C-7), 131.14 (C-5’), 133.16 (C-2’), 134.61 (C-8), 138.84 (C-10), 145.12 (C-β), 146.55

(C-2), 153.18 (C-4), 156.06 (C-4’). ESI-MS **4b** in MeOH, m/z: calcd. 301.17; found 301.00 $[M]^+$. Anal. calcd. for $C_{21}H_{21}I^{-}N_2^{+}$: C, 58.89 %; H, 4.94 %; N, 6.54 %; found C, 58.73 %; H, 4.80 %; N, 6.47 %.

(E)-1-methyl-4-(2-(4-methyl-3,4-dihydro-2H-benzo[b][1,4]oxazin-7-yl)vinyl)quinolin-1-ium iodide (5b)

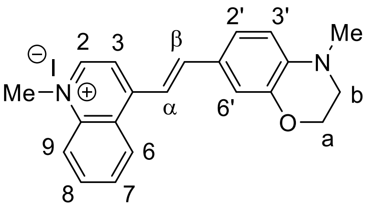

Dark solid. Yield 40%. M.p. 264-265 ℃. ^{1}H NMR (400 MHz, DMSO-d_6, δ ppm., *J* Hz): 3.00 (s, 3H, N-CH_3), 3.44 (m, 2H, H-b), 4.25 (m, 2H, H-a), 4.45 (m, 3H, N^+-CH_3), 6.78 (d, 1H, H-3’, *J* = 8.3), 7.40 (d, 1H, H-2’, *J* = 8.3), 7.52 (s, 1H, H-6’), 7.96 (m, 2H, H-α,7), 8.11 (d, 1H, H-β, J_{trans} = 15.7), 8.20 (t, 1H, H-8, *J* = 7.9), 8.33 (m, 2H, H-3,9), 9.05 (d, 1H, H-6, *J* = 8.4), 9.13 (d, 1H, H-2, *J* = 6.5). ^{13}C NMR (101 MHz, DMSO-d_6, δ ppm., *J* Hz): 37.83 (N-CH_3), 44.05 (N^+-CH_3), 48.11 (C-b), 63.79 (C-a), 111.30 (C-3’), 114.03 (C-3), 114.13 (C-α), 114.61 (C-6’), 119.00 (C-9), 124.45 (C-5), 125.82 (C-1’), 125.88 (C-2’), 126.43 (C-6), 128.57 (C-7), 134.61 (C-8), 138.75 (C-10), 139.90 (C-4’), 143.48 (C-5’), 144.40 (C-β), 146.86 (C-2), 153.00 (C-4). ESI-MS **5b** in MeOH, m/z: calcd. 317.16; found 317.30 $[M]^+$. Anal. calcd. for $C_{21}H_{21}I^{-}N_2^{+}O$: C, 56.77; H, 4.76 %; N, 6.30 %; found C, 56.93; H, 4.86 %; N, 6.31 %.

(E)-4-(4-(dimethylamino)styryl)-1-methylquinolin-1-ium iodide (6b)

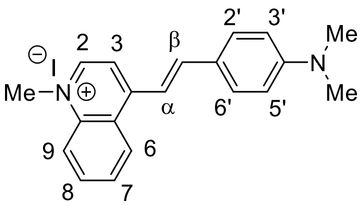

Blue solid. Yield 47%. M.p. 260-262 ℃. ^{1}H NMR (400 MHz, DMSO-d_6, δ ppm., *J* Hz): 3.07 (s, 6H, N-$(CH_3)_2$), 4.44 (s, 3H, N^+-CH_3), 6.82 (d, 2H, H-3’,5’, *J* = 8.8), 7.87 (d, 2H, H-2’,6’, *J* = 8.8), 7.96 (d, 1H, H-7, *J* = 7.7), 8.01 (d, 1H, H-α, *J* = 15.1), 8.18 (m, 2H, H-β,8), 8.33 (m, 2H, H-3,9), 9.03 (d, 1H, H-6, *J* = 8.6), 9.11 (d, 1H, H-2, *J* = 6.7). ^{13}C NMR (101 MHz, DMSO-d_6, δ ppm., *J* Hz): 39.10 (N$(CH_3)_2$), 44.04 (N^+-CH3), 111.91 (C-3’,5’), 113.11 (C-α), 113.94 (C-3), 119.06 (C-9), 123.06 (C-5), 125.76 (C-6), 126.36 (C-1’), 128.56 (C-7), 131.36 (C-2’,6’), 134.62 (C-8), 138.80 (C-10), 144.70 (C-β), 146.78 (C-2), 152.30 (C-4’), 153.16 (C-4) [52]. Anal. calcd. for $C_{20}H_{21}I^{-}N_2^{+}$: C, 57.70 %; H, 5.08 %; N, 6.73 %; found C, 57.65 %; H, 5.00 %; N, 6.64 %.

(E)-4-(4-(diphenylamino)styryl)-1-methylquinolin-1-ium iodide (7b)

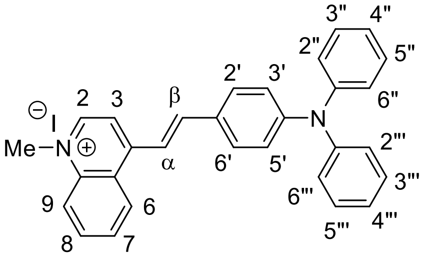

Dark powder. Yield 30%. M.p. 182-184 ℃. ^{1}H NMR (600 MHz, DMSO-d_6, δ ppm., *J* Hz): 4.52 (s, 3H, N^+-CH_3), 6.96 (d, 2H, H-3’,5’, *J* = 8.8), 7.15 (d, 4H, H-2”,6”,2’”,6’”, *J* = 7.6), 7.18 (t, 2H, H-4”,4’”, *J* = 7.6), 7.40 (t, 4H, H-3”,5”,3’”,5’”, *J* = 7.9), 7.90 (d, 2H, H-2’,6’, *J* = 8.8), 8.02 (t, 1H, H-7, *J* = 7.8), 8.17 (s, 2H, H-α,β), 8.24 (t, 1H, H-8, *J* = 8.4), 8.41 (d, 1H, H-9, *J* = 8.8), 8.47 (d, 1H, H-3, *J* = 6.7), 9.03 (d, 1H, H-6, *J* = 7.8), 9.32 (d, 1H, H-2, *J* = 6.7). ^{13}C NMR (151 MHz, DMSO-d_6, δ ppm., *J* Hz): 44.46 (N^+-CH_3), 115.38 (C-3), 116.94 (C-α), 119.36 (C-9), 120.47 (C-3’,5’), 124.71 (C-4”,4’”), 125.55 (C-2”,6”,2’”,6’”), 126.15 (C-6), 126.44 (C-5), 128.48 (C-1’), 129.07 (C-7), 129.94 (C-3”,5”,3’”,5’”), 130.64 (C-2’,6’), 134.89 (C-8), 138.83 (C-10), 142.98 (C-β), 146.15

(C-1”,1”’), 147.72 (C-2), 149.82 (C-4’), 152.79 (C-4) [53]. Anal. calcd. for $C_{30}H_{25}I^{-}N_2^{+}$: C, 66.67 %; H, 4.66 %; N, 5.18 %; found C, 66.44 %; H, 4.31 %; N, 5.23 %.

(E)-1-methyl-4-(2-(1-propyl-1,2,3,4-tetrahydroquinolin-6-yl)vinyl)quinolin-1-ium iodide (8b)

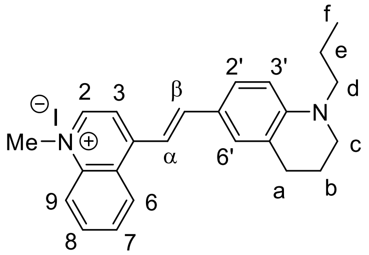

Dark solid. Yield 29%. M.p. 180-182 ℃. ^{1}H NMR (600 MHz, DMSO-d_6, δ ppm., J Hz): 0.92 (t, 3H, H-f, J = 7.4), 1.58 (h, 2H, H-e, J = 7.4), 1.88 (p, 2H, H-b, J = 6.4), 2.76 (t, 2H, H-a, J = 6.1), 3.33 (d, 2H, H-d, J = 7.9), 3.40 (d, 2H, H-c, J = 6.3), 4.39 (s, 3H, N^{+}-CH_3), 6.69 (d, 1H, H-3’, J = 8.8), 7.58 (d, 1H, H-2’, J = 8.4), 7.67 (s, 1H, H-6’), 7.90 (d, 1H, H-α, J_{trans} = 15.5), 7.94 (t, 1H, H-7, J = 7.7), 8.10 (d, 1H, H-β, J_{trans} = 15.5), 8.17 (t, 1H, H-8, J = 8.3), 8.25 (d, 1H, H-3, J = 6.8), 8.29 (d, 1H, H-9, J = 8.8), 8.98 (d, 1H, H-6, J = 8.9), 9.00 (d, 1H, H-2, J = 6.8). ^{13}C NMR (151 MHz, DMSO-d_6, δ ppm., J Hz): 11.71 (C-f), 19.70 (C-e), 21.60 (C-b), 27.95 (C-a), 44.30 (N^{+}-CH_3), 49.62 (C-c), 52.58 (C-d), 110.69 (C-3’), 112.35 (C-α), 113.76 (C-3), 119.38 (C-9), 122.66 (C-5’), 122.82 (C-1’), 126.04 (C-6), 126.67 (C-5), 128.84 (C-7), 130.21 (C-6’), 131.56 (C-2’), 134.98 (C-8), 139.24 (C-10), 145.43 (C-β), 146.84 (C-2), 148.79 (C-4’), 153.49 (C-4). ESI-MS **8b** in MeOH, m/z: calcd. 343.22; found 343.00 $[M]^{+}$. Anal. calcd. for $C_{24}H_{27}I^{-}N_2^{+}$: C, 61.28 %; H, 5.79 %; N, 5.96 %; found C, 60.98 %; H, 5.81 %; N, 5.89 %.

(E)-1-methyl-4-(2-(2,3,6,7-tetrahydro-1H,5H-pyrido[3,2,1-ij]quinolin-9-yl)vinyl)quinolin-1-ium iodide (9b)

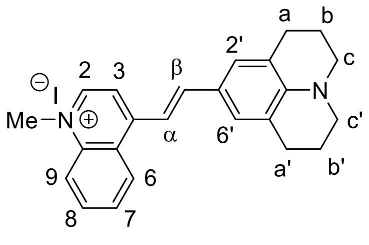

Dark solid. Yield 53%. M.p. 233-234 ℃. ^{1}H NMR (400 MHz, DMSO-d_6, δ ppm., J Hz): 1.89 (p, 4H, H-b,b’, J = 6.3), 2.74 (t, 4H, H-a,a’, J = 6.1), 3.31 (t, 4H, H-c,c’, J = 5.9), 4.38 (s, 3H, N^{+}-CH_3), 7.45 (s, 2H, H-2',6'), 7.87 (d, 1H, H-α J_{trans} = 15.6), 7.92 (t, 1H, H-7, J = 7.7), 8.08 (d, 1H, H-β, J_{trans} = 15.6), 8.16 (t, 1H, H-8, J = 7.8), 8.23 (d, 1H, H-3, J = 6.8), 8.28 (d, 1H, H-9, J = 8.9), 8.98 (d, 1H, H-6, J = 8.9), 9.01 (d, 1H, H-2, J = 6.9). ^{13}C NMR (101 MHz, DMSO-d_6, δ ppm., J Hz): 21.33 (C-b,b’), 27.53 (C-a,a’), 44.11 (N^{+}-CH_3), 49.85 (C-c,c’), 111.97 (C-α), 113.46 (C-3), 119.31 (C-9), 121.32 (C-3',5'), 122.50 (C-1’), 125.97 (C-5), 126.65 (C-6), 128.65 (C-7), 129.55 (C-2’,6’), 134.85 (C-7), 139.25 (C-10), 145.67 (C-β), 146.42 (C-4’), 146.68 (C-2), 153.38 (C-4) [54]. ESI-MS **9b** in MeOH, m/z: calcd. 341.20; found 341.00 $[M]^{+}$. Anal. calcd. for $C_{24}H_{25}I^{-}N_2^{+}$: C, 61.54 %; H, 5.38 %; N, 5.98 %; found C, 61.32 %; H, 5.56 %; N, 5.84 %.

2.^{1}H and ^{13}C, 2D NMR spectra of compounds

1,4-dimethylpyridin-1-ium iodide (1a) in DMSO-d_6

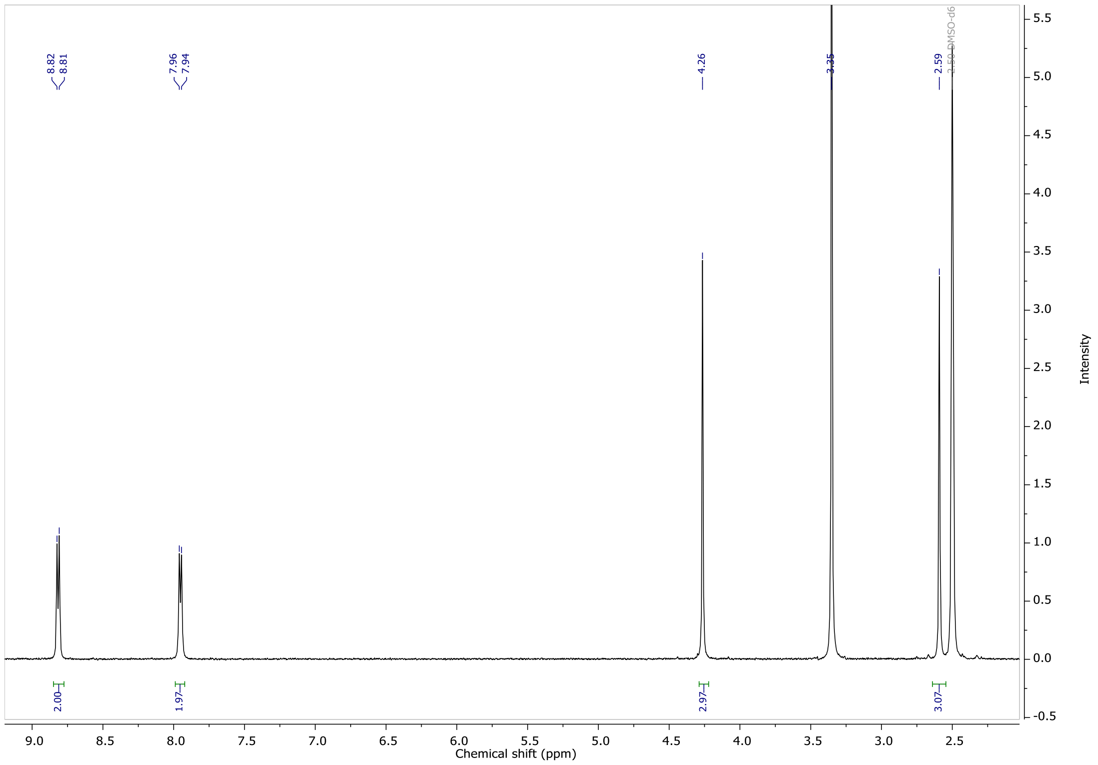

1,4-dimethylquinolin-1-ium iodide (1b) in DMSO-d_6

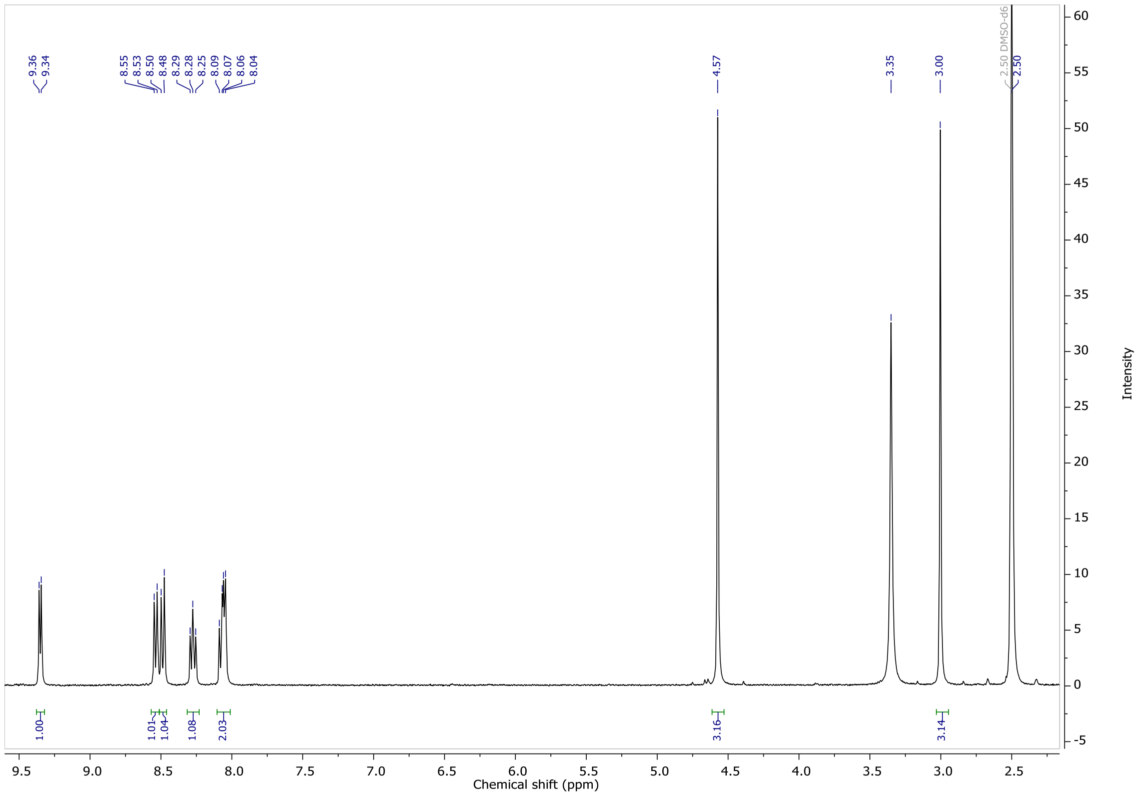

(E)-4-(2-(9-ethyl-9H-carbazol-3-yl)vinyl)-1-methylpyridin-1-ium iodide (3a) in DMSO-d_6

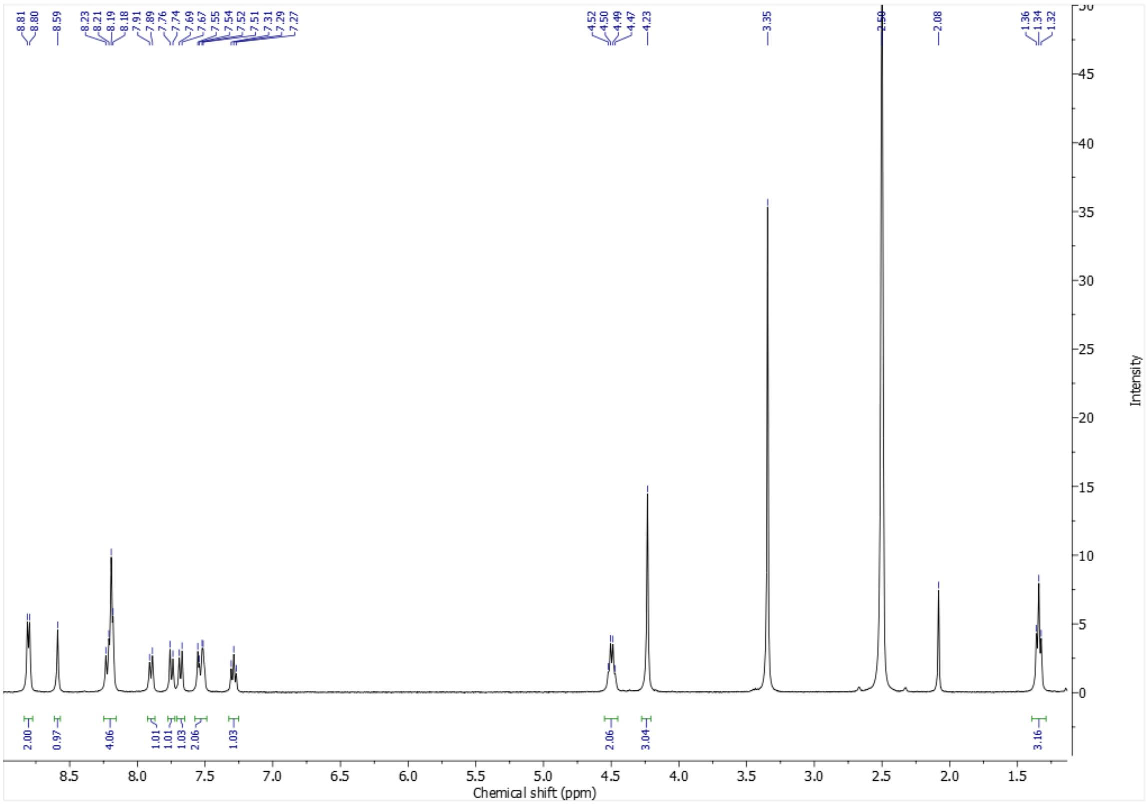

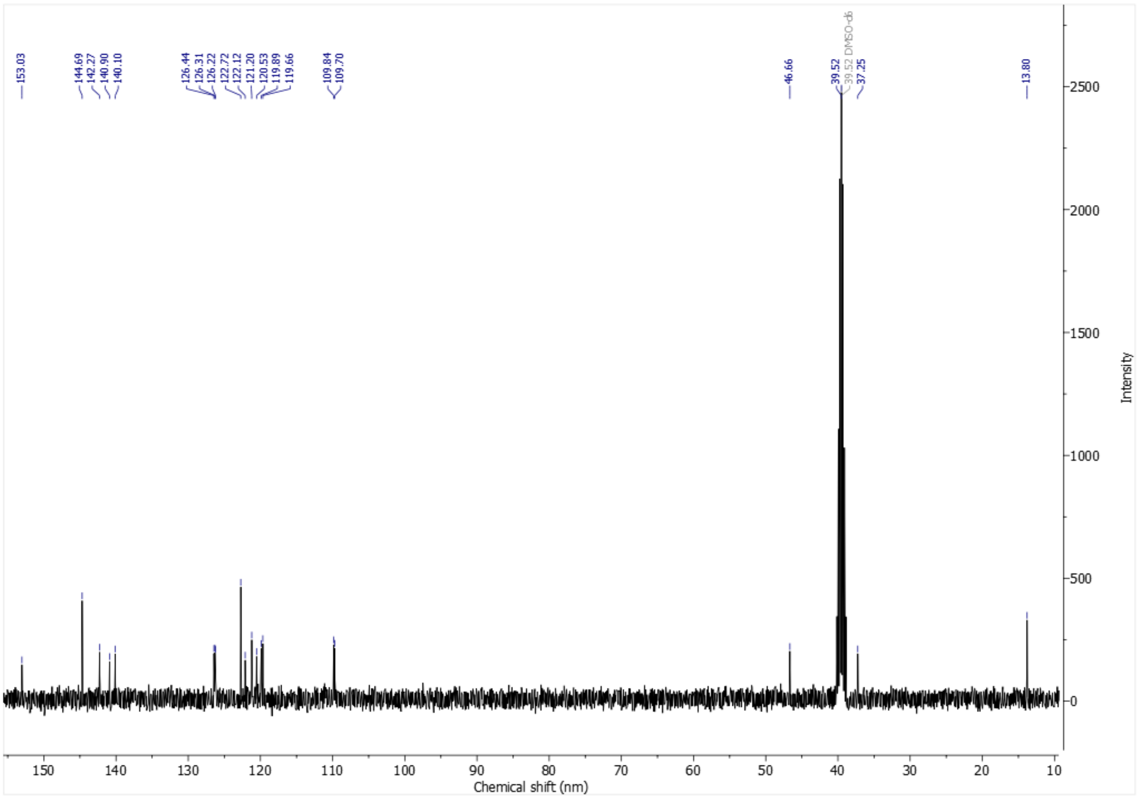

(E)-1-methyl-4-(2-(1-methylindolin-5-yl)vinyl)pyridin-1-ium iodide (4a) DMSO-d_6

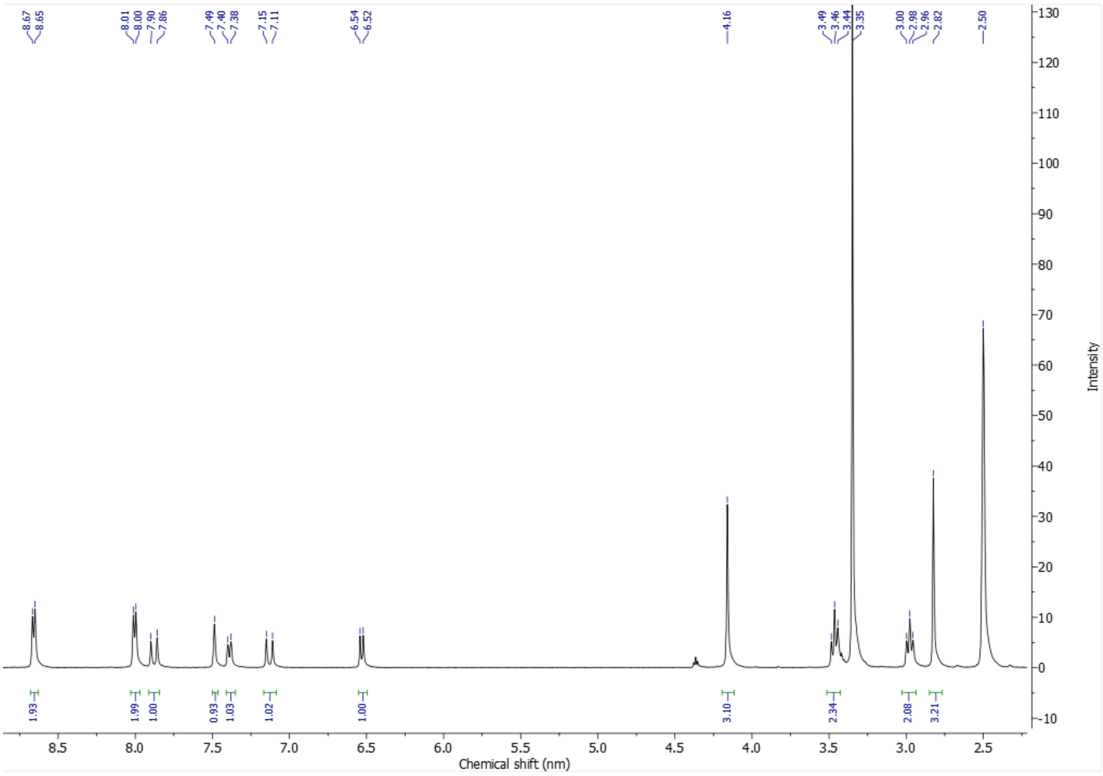

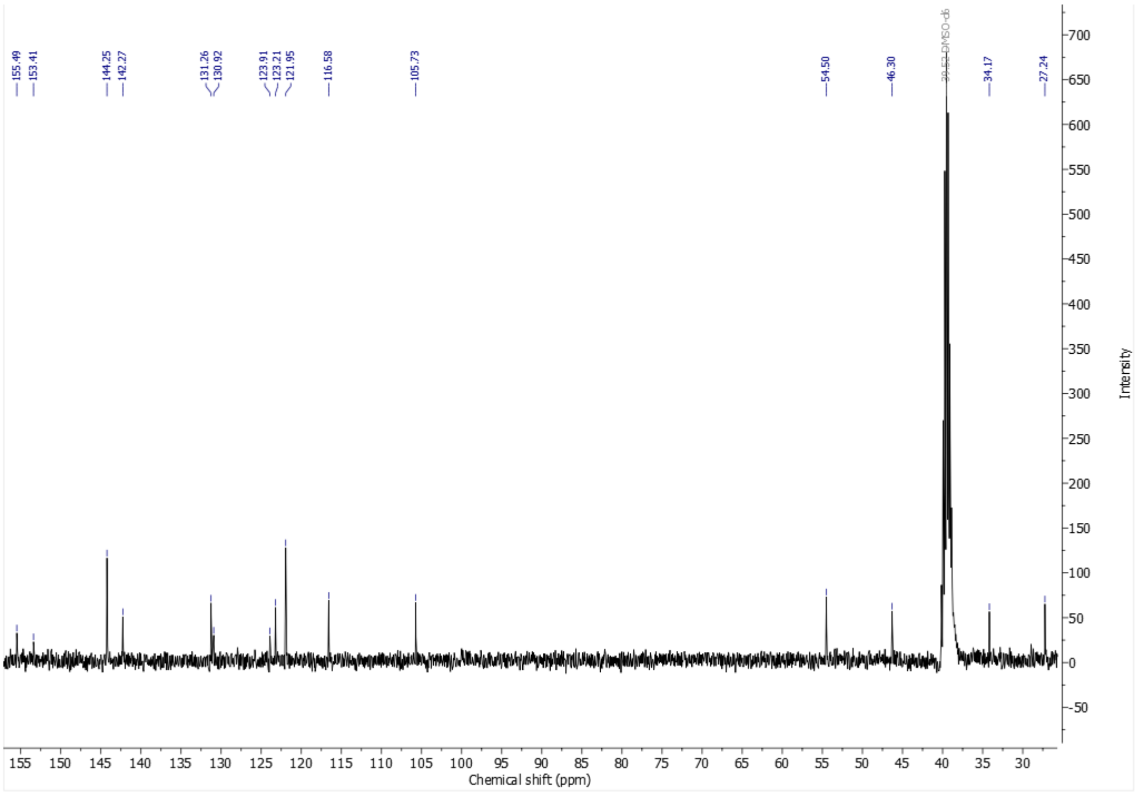

COSY 4a

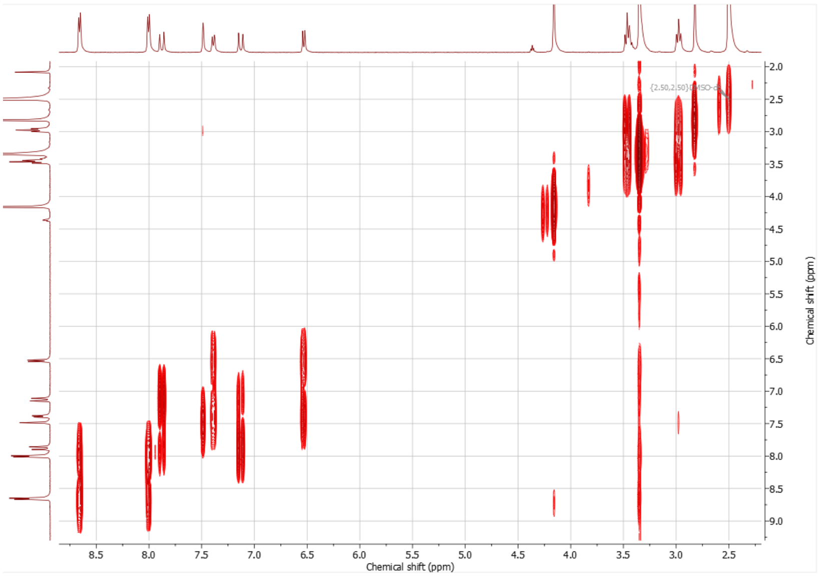

HSQC 4a

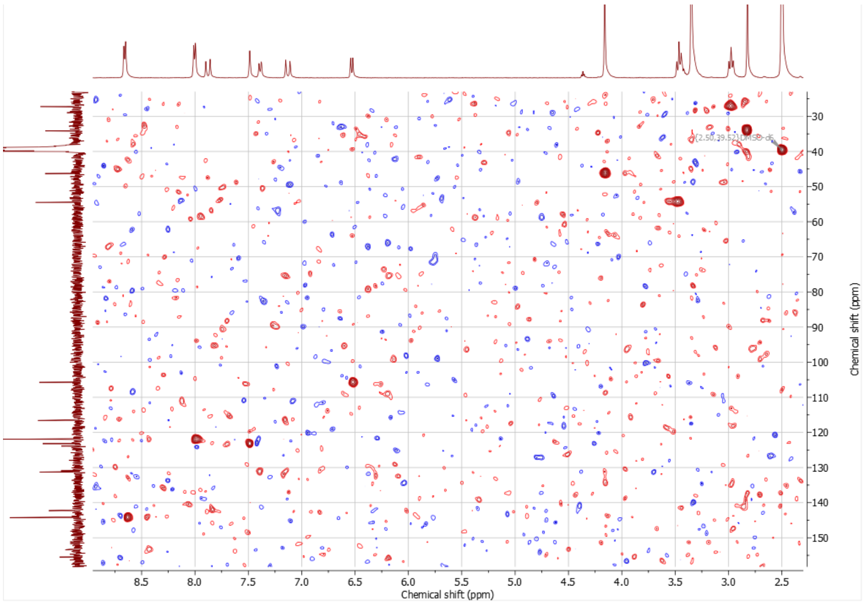

HMBC 4a

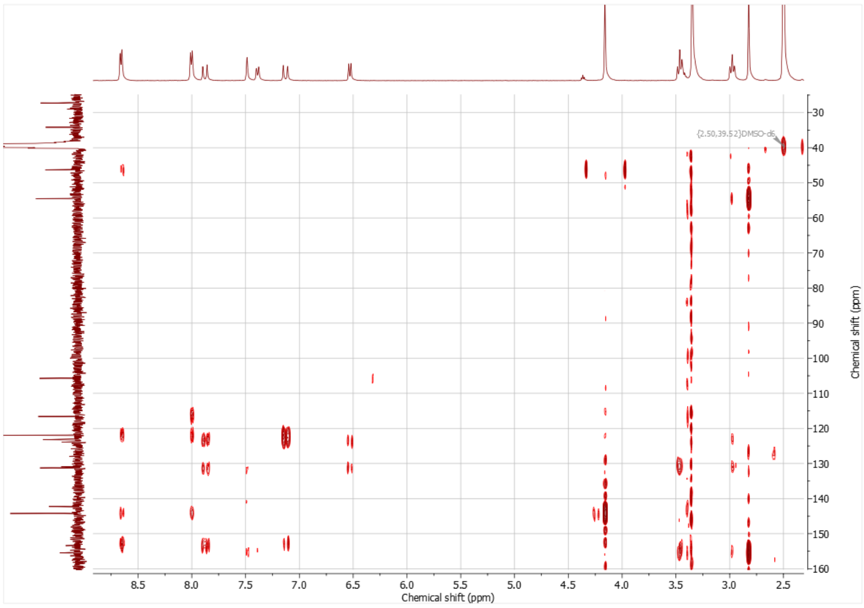

(E)-1-methyl-4-(2-(4-methyl-3,4-dihydro-2H-benzo[b][1,4]oxazin-7-yl)vinyl)pyridin-1-ium iodide (5a) in DMSO-d_6

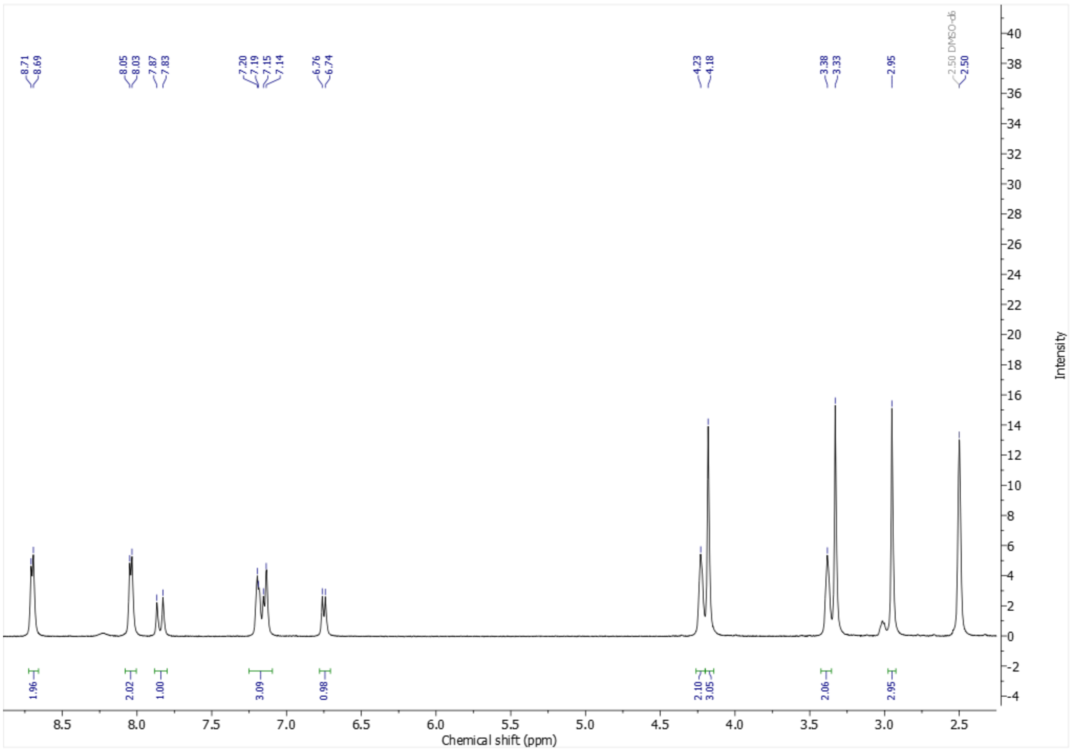

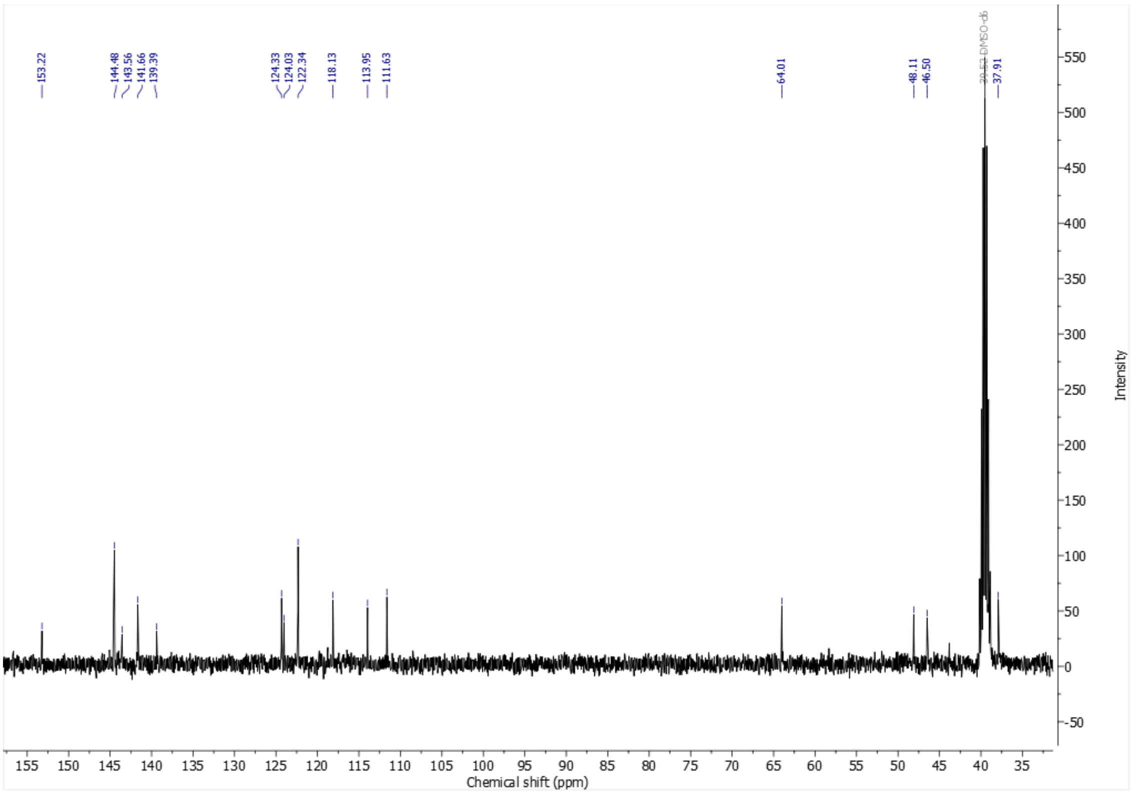
153.22
144.48
143.56
141.66
139.39
124.33
124.03
122.34
118.13
113.95
111.63
64.01
48.11
46.50
39.52 DMSO-d6
37.91
Intensity
Chemical shift (ppm)

COSY 5a

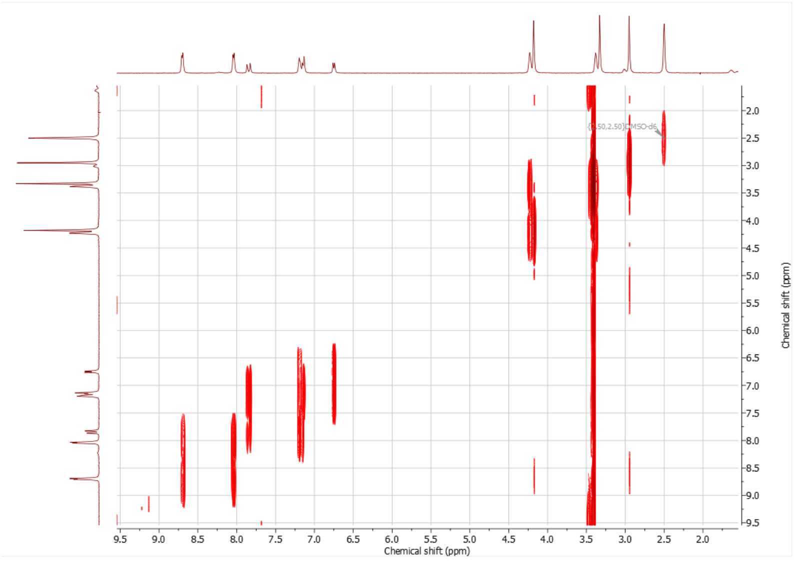

HSQC 5a

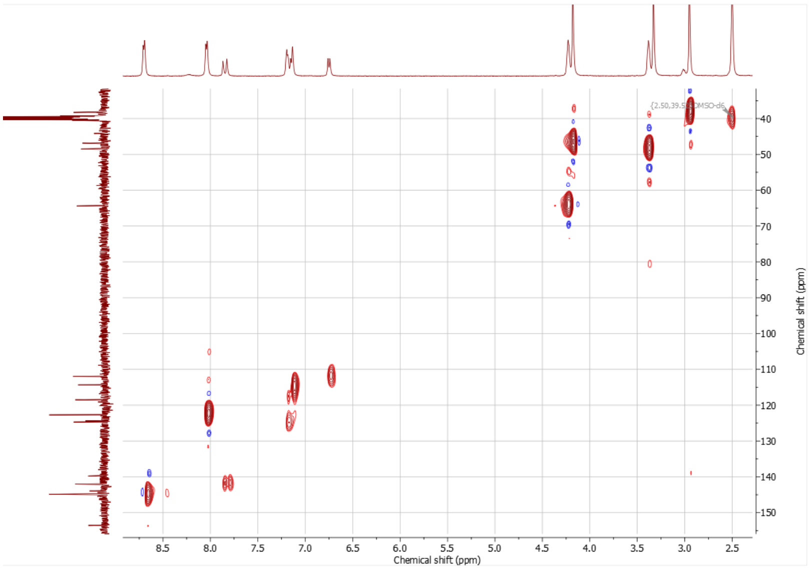

HMBC 5a

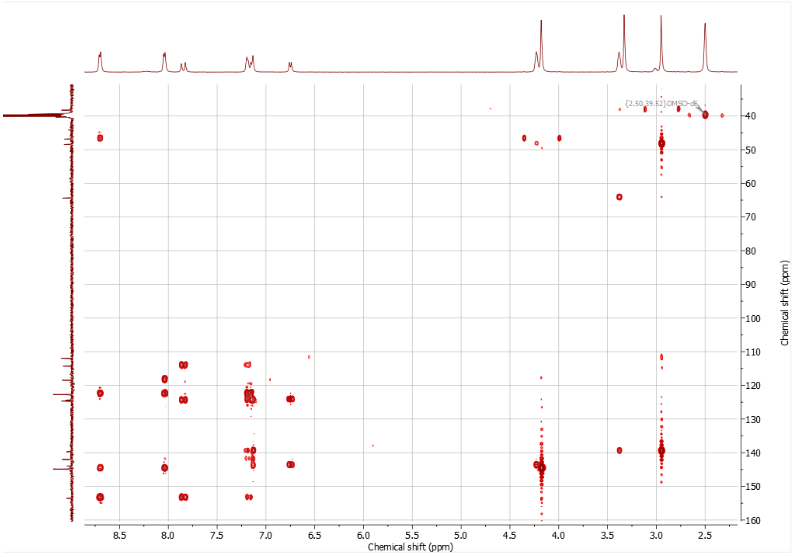

(E)-4-(4-(dimethylamino)styryl)-1-methylpyridin-1-ium iodide (6a, DASPI) in DMSO-d_6

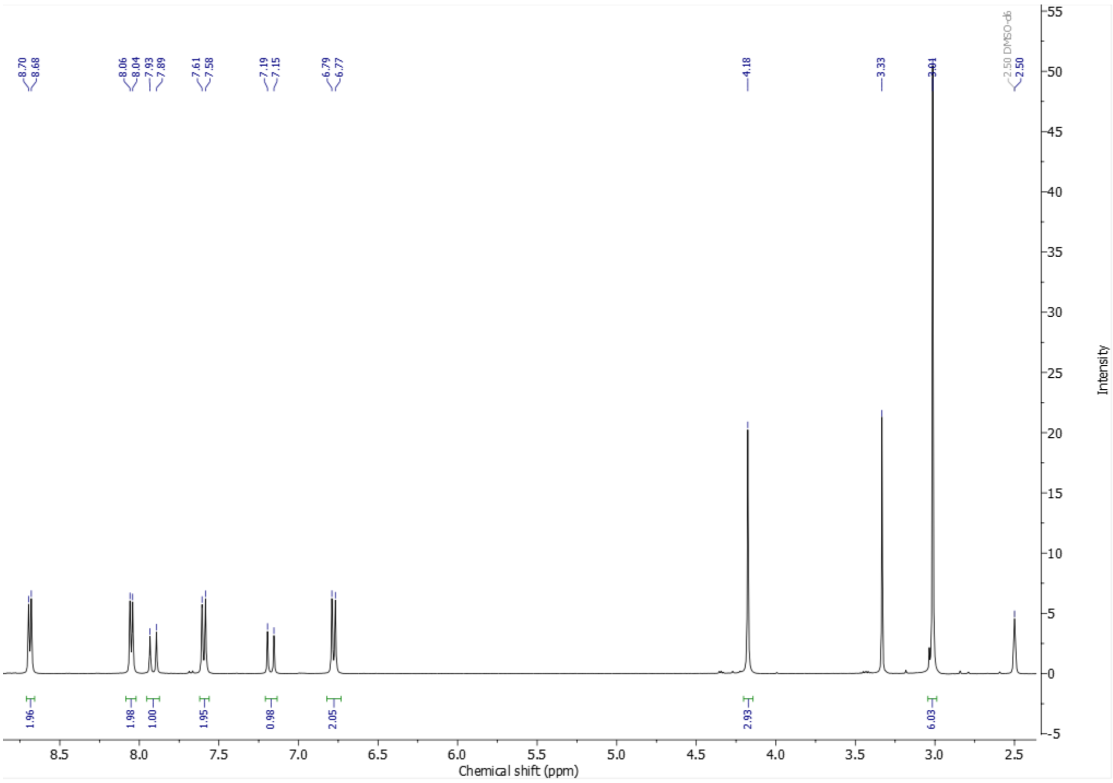

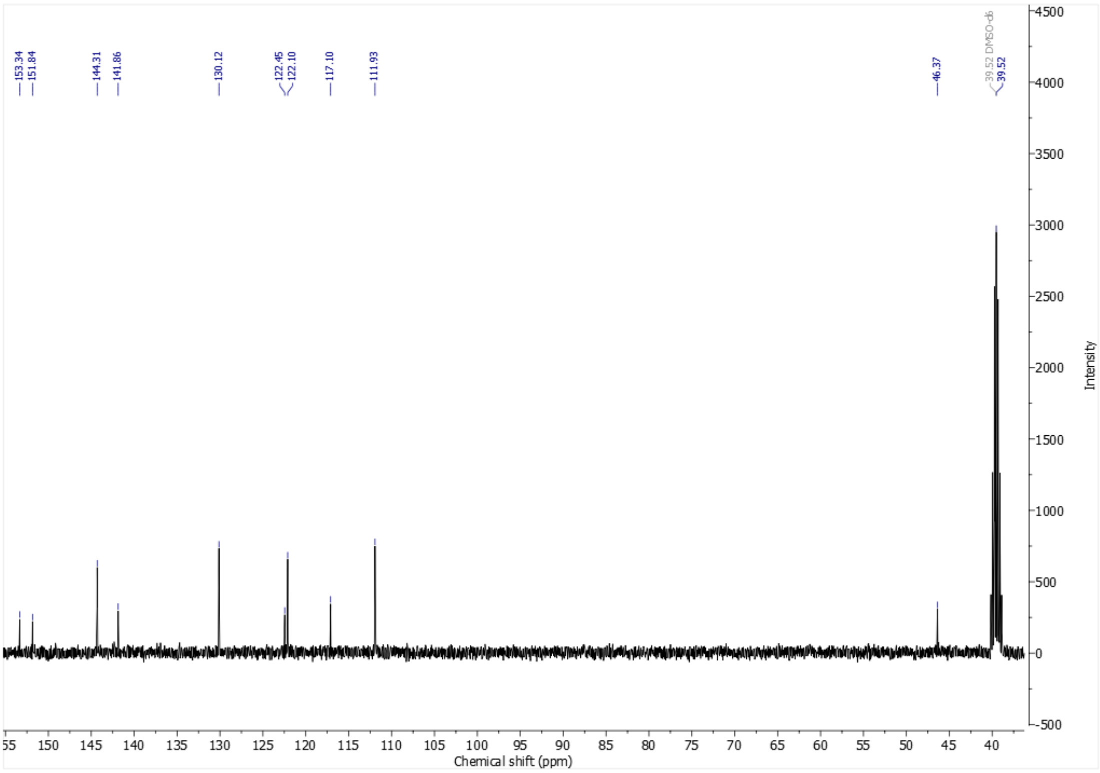

(E)-4-(4-(diphenylamino)styryl)-1-methylpyridin-1-ium iodide (7a) in DMSO-d_6

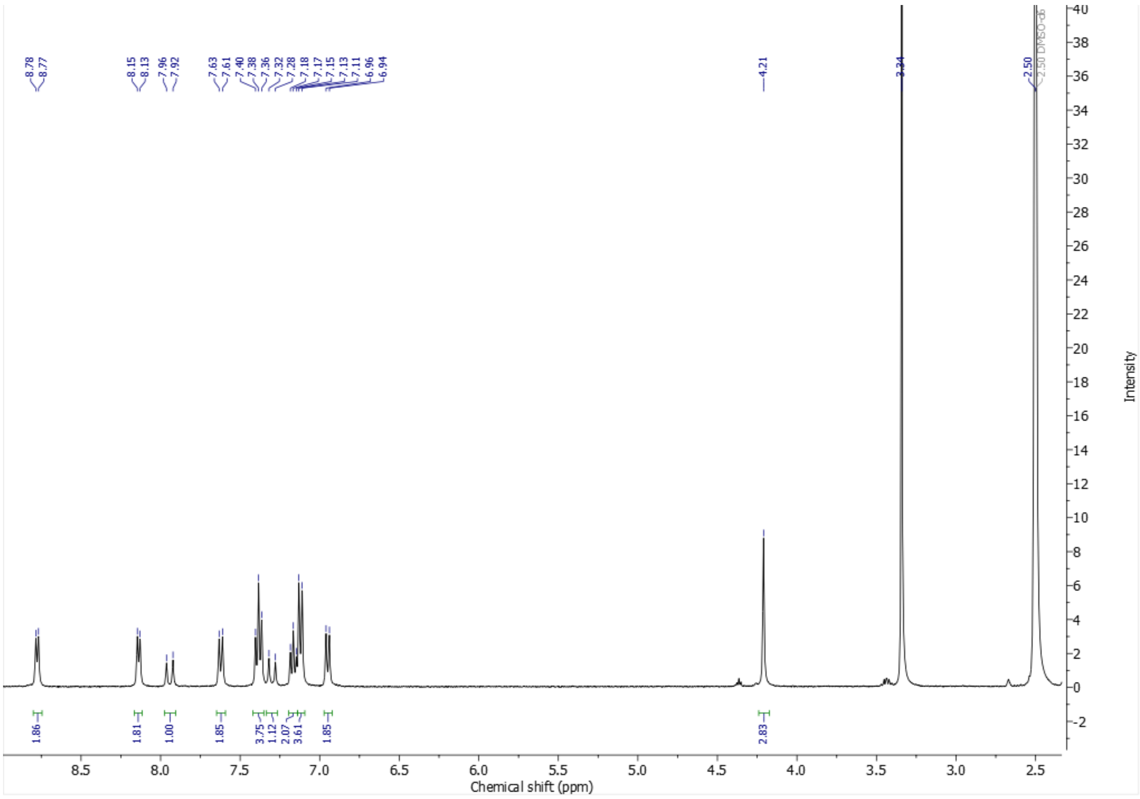

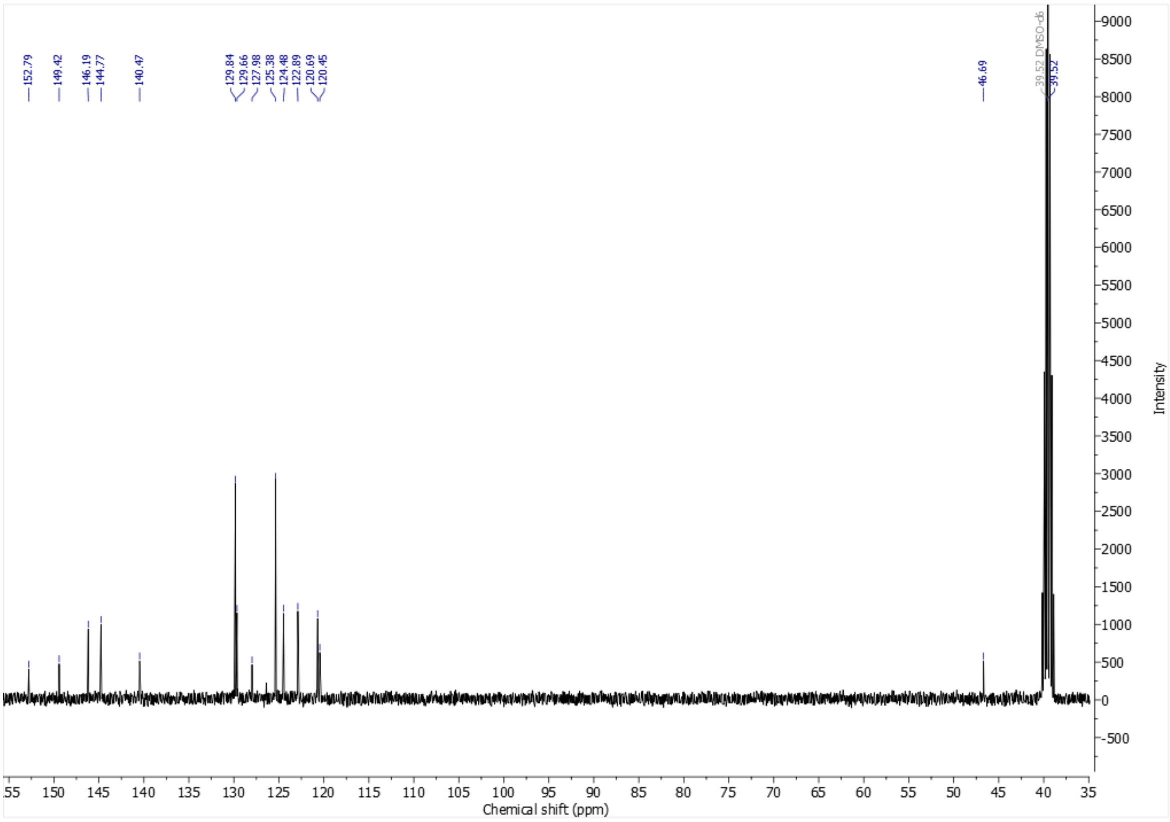

(E)-1-methyl-4-(2-(1-propyl-1,2,3,4-tetrahydroquinolin-6-yl)vinyl)pyridin-1-ium iodide (8a) in DMSO-d$_6$

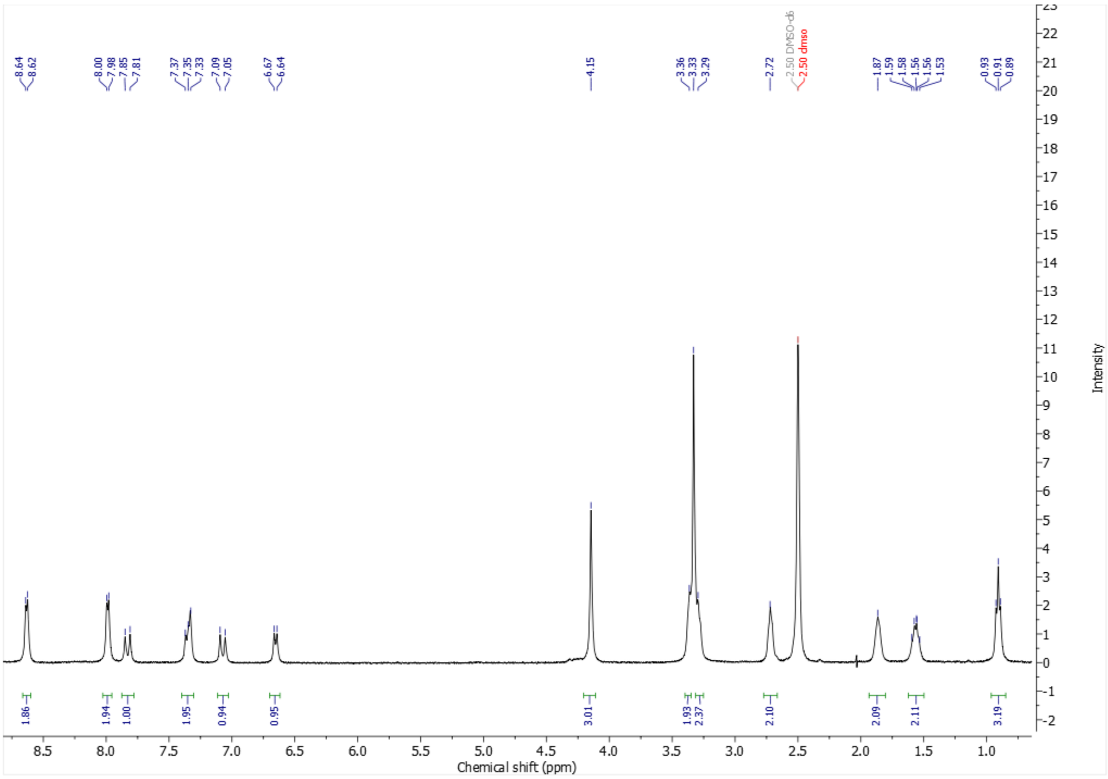

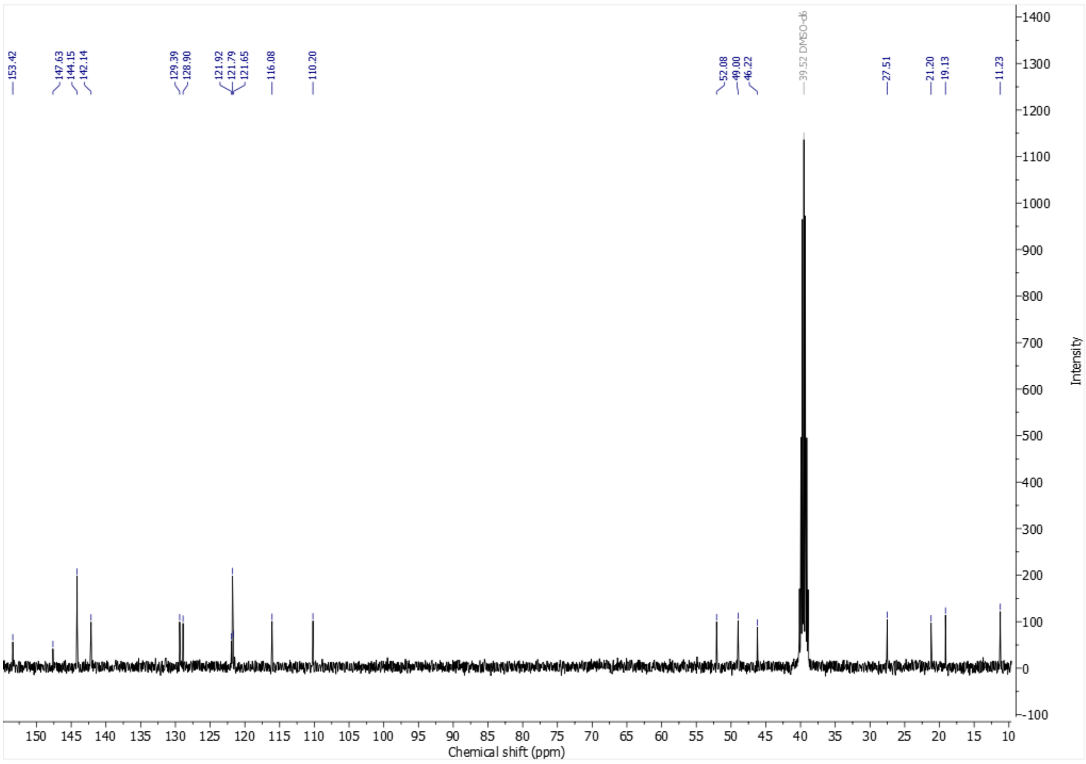

COSY 8a

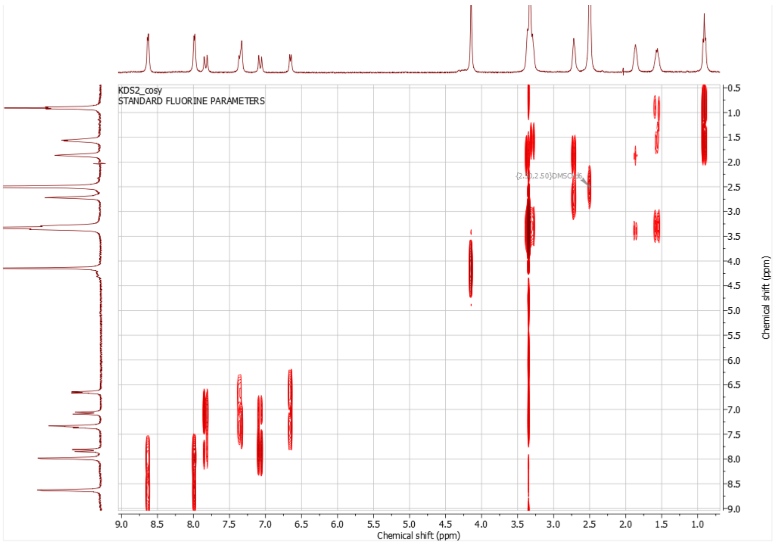

HSQC 8a

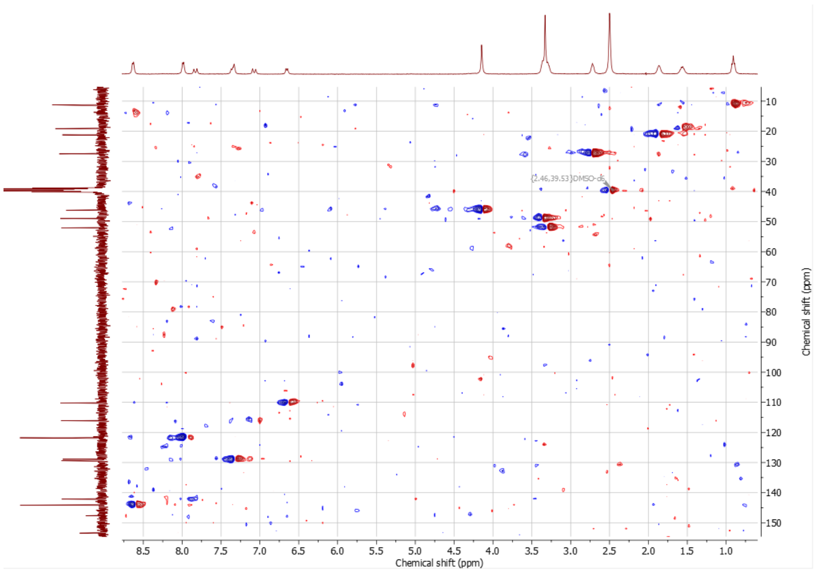

HMBC 8a

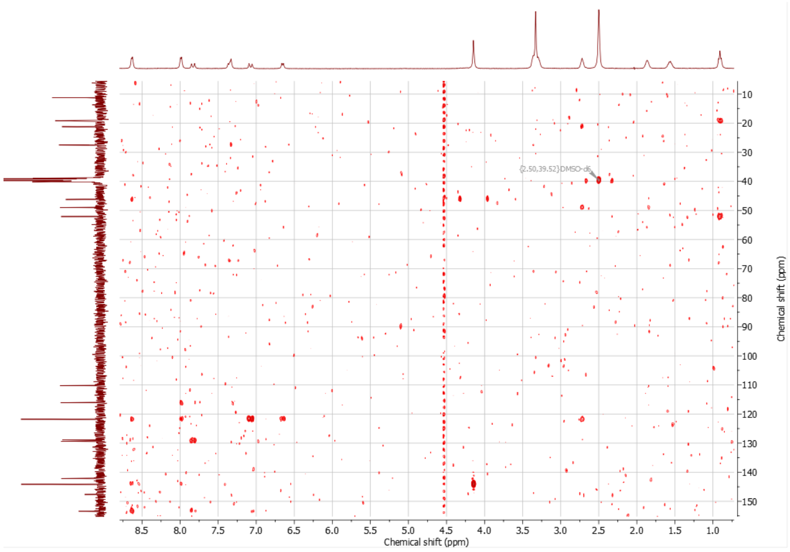

(E)-1-methyl-4-(2-(2,3,6,7-tetrahydro-1H,5H-pyrido[3,2,1-ij]quinolin-9-yl)vinyl)pyridin-1-ium iodide (9a) in DMSO-d₆

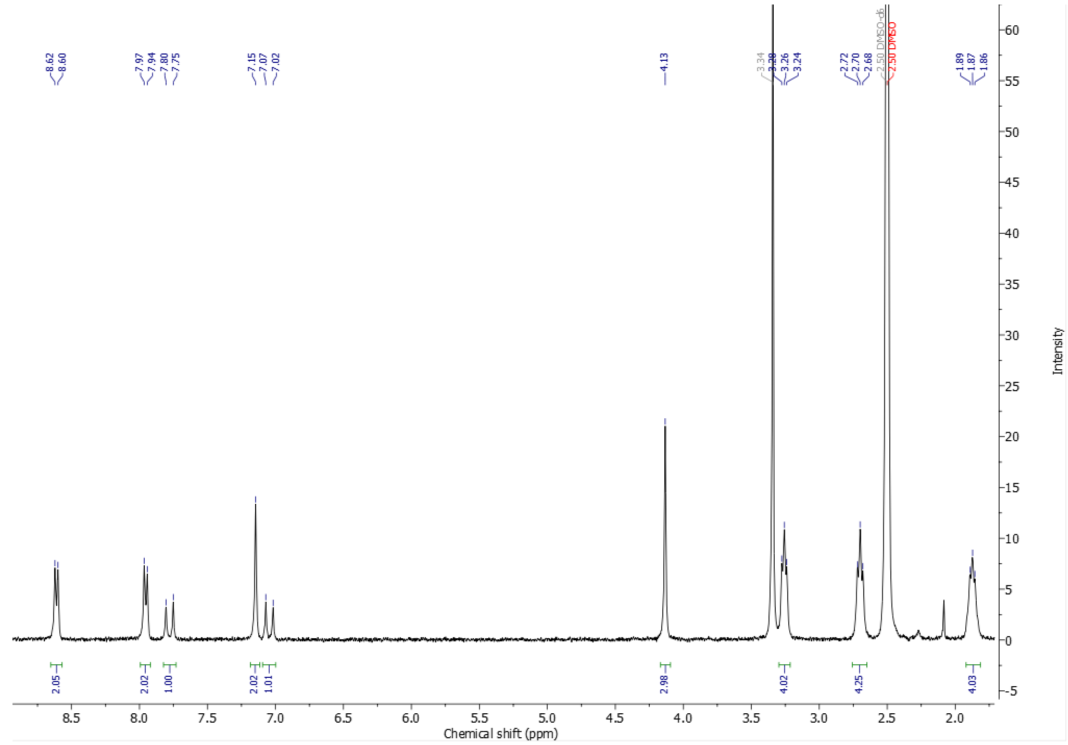

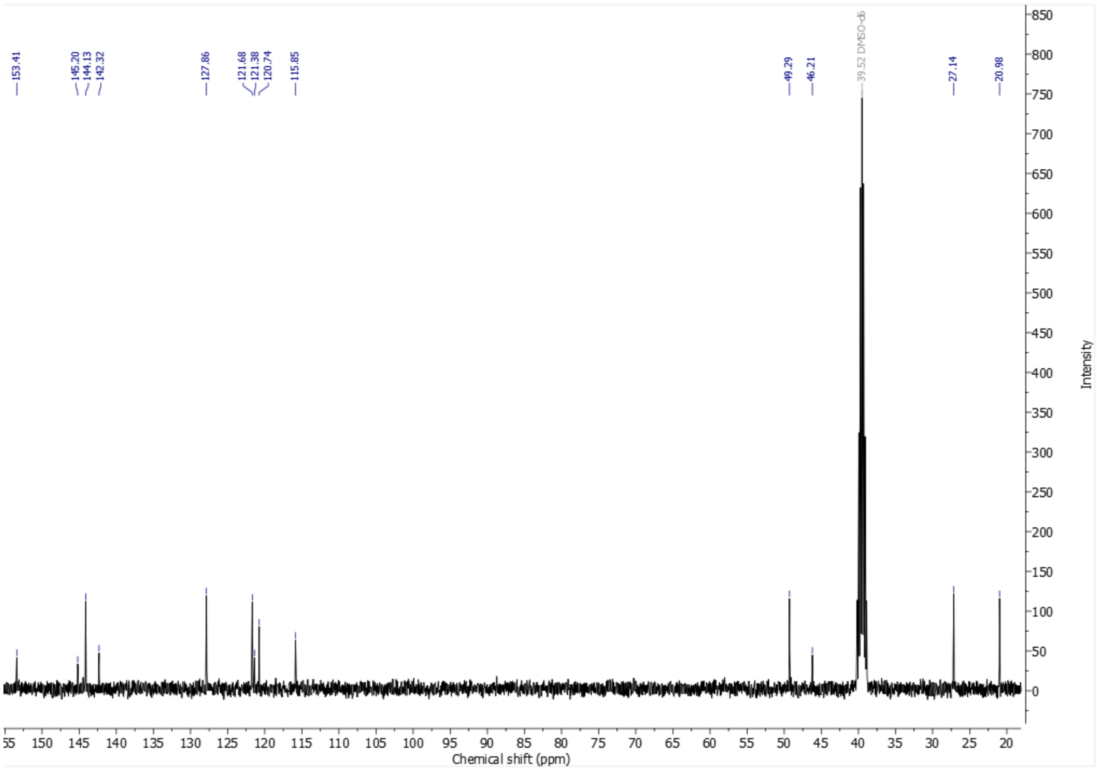

COSY 9a

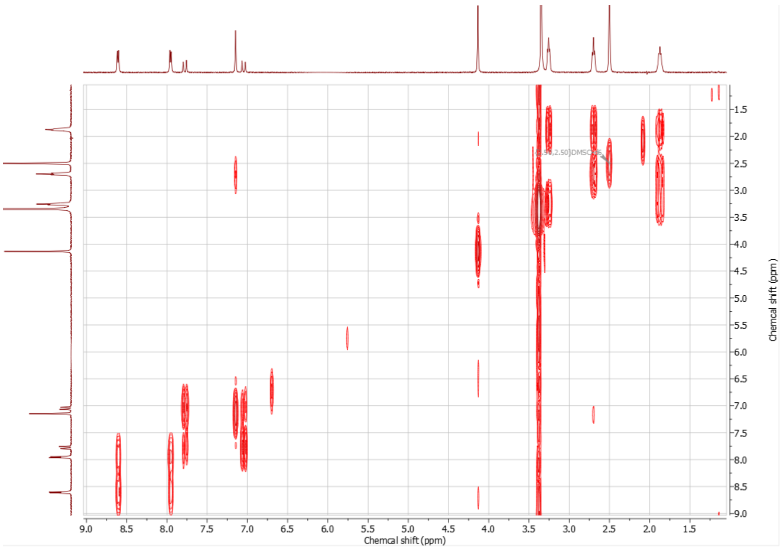

HSQC 9a

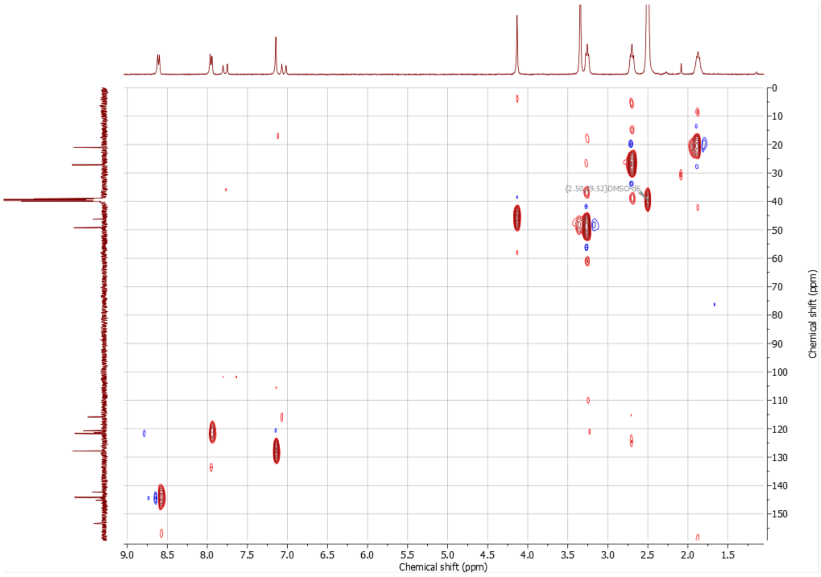

HMBC 9a

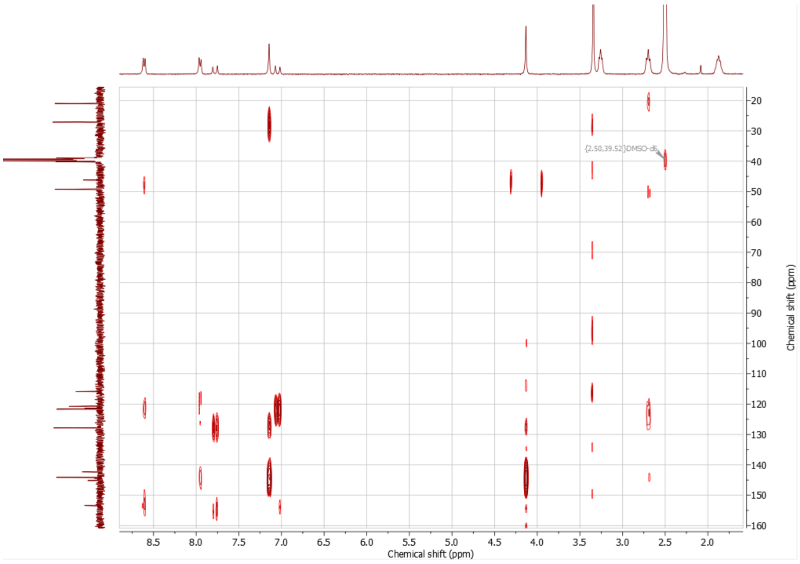

(E)-4-(2-(9-ethyl-9H-carbazol-3-yl)vinyl)-1-methylquinolin-1-ium iodide (3b) in DMSO-d_6

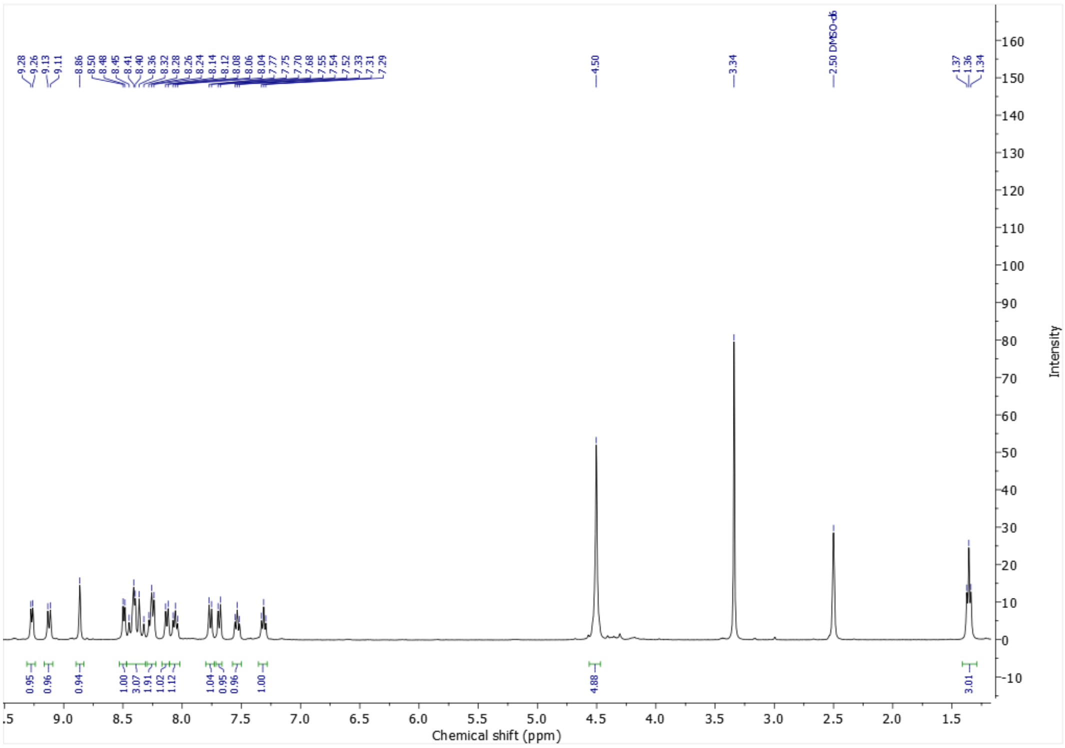

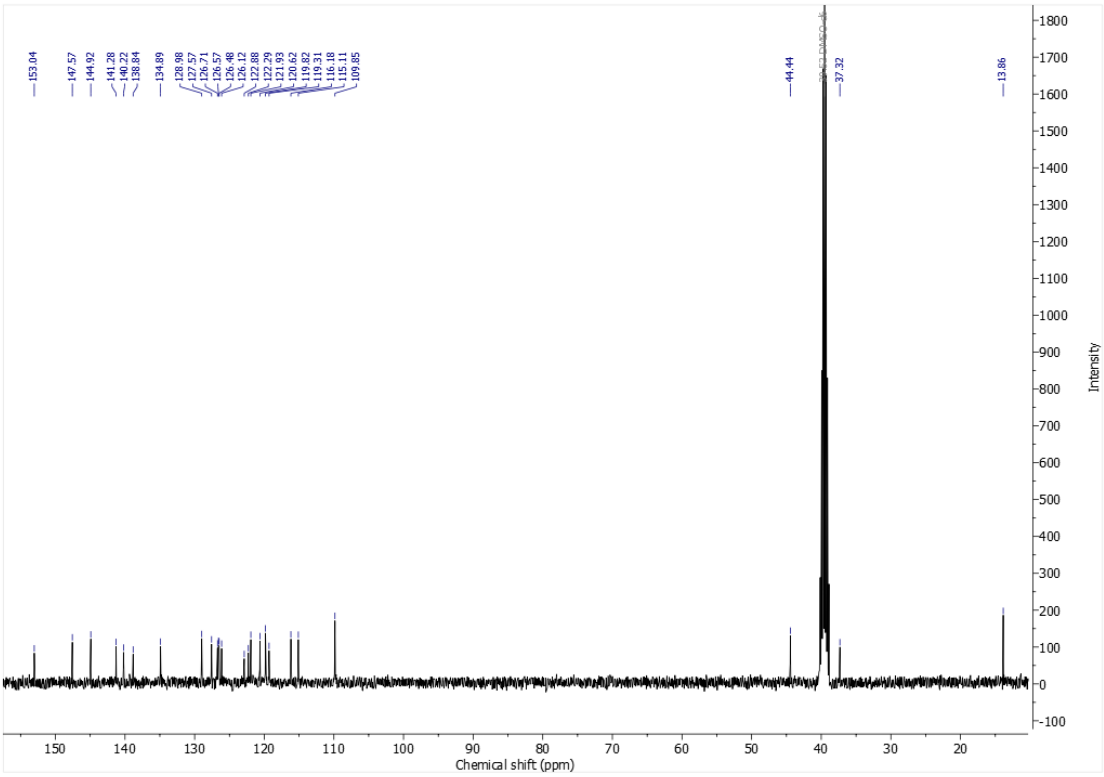
153.04
147.57
144.92
141.28
140.22
138.84
134.89
128.98
127.57
126.71
126.57
126.48
126.12
122.88
122.29
121.93
120.62
119.82
119.31
116.18
115.11
109.85
44.44
37.32
13.86
Chemical shift (ppm)
Intensity

COSY 3b

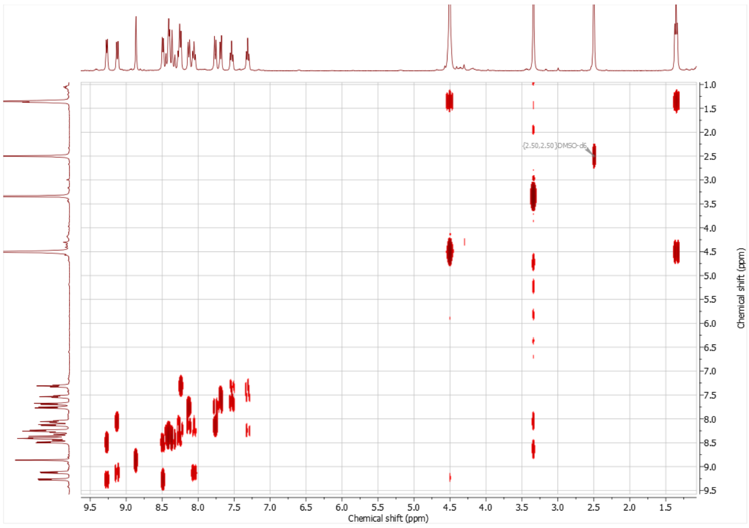

HSQC 3b

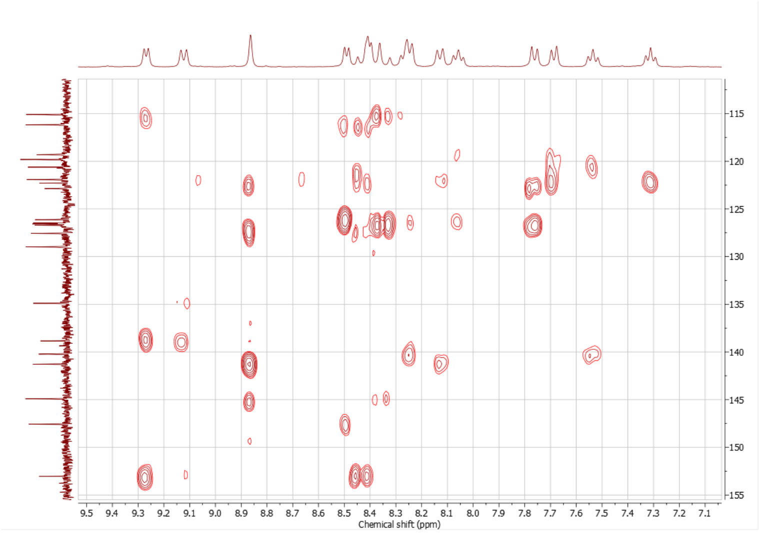

HMBC 3b

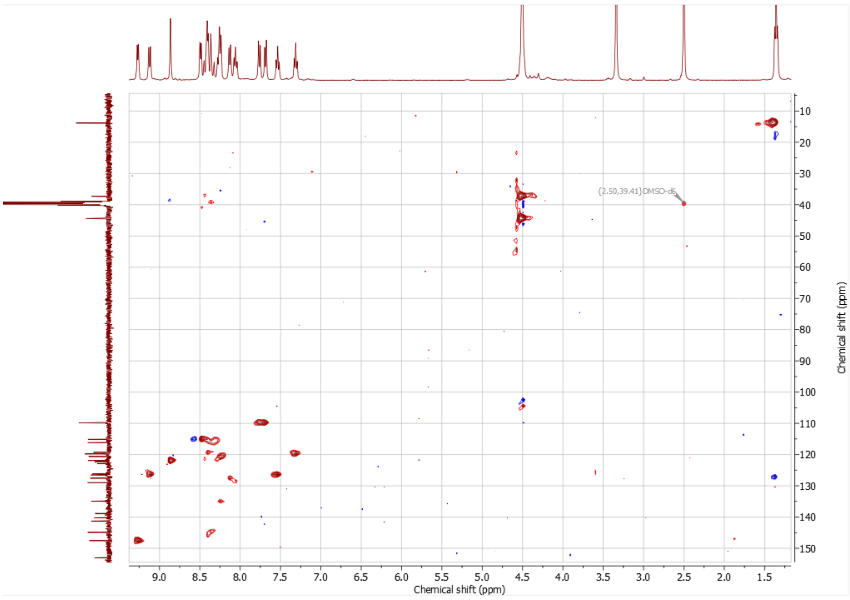

(E)-1-methyl-4-(2-(1-methylindolin-5-yl)vinyl)quinolin-1-ium iodide (4b) in DMSO-d_6

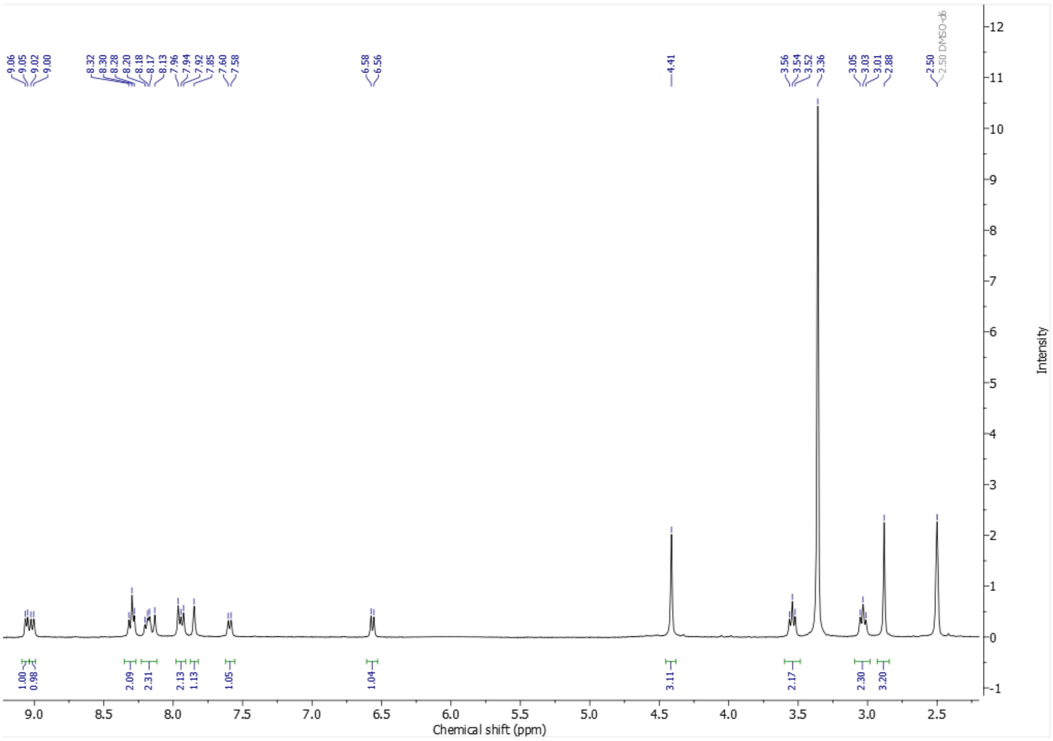

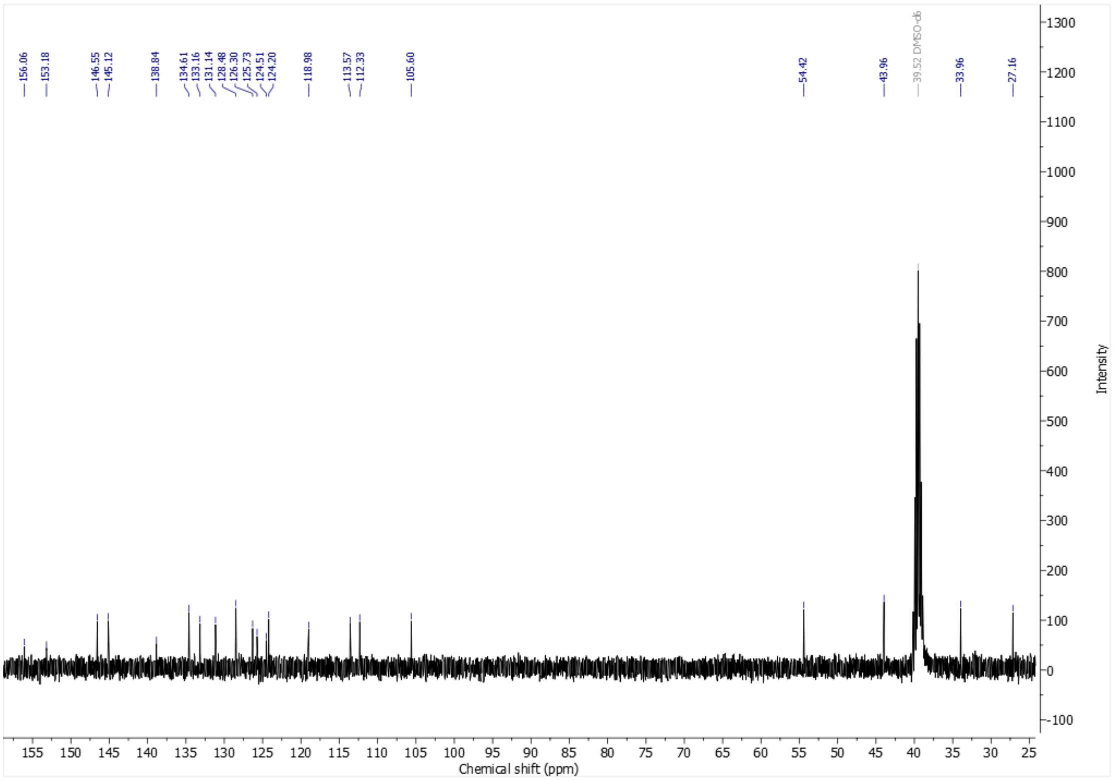

COSY 4b

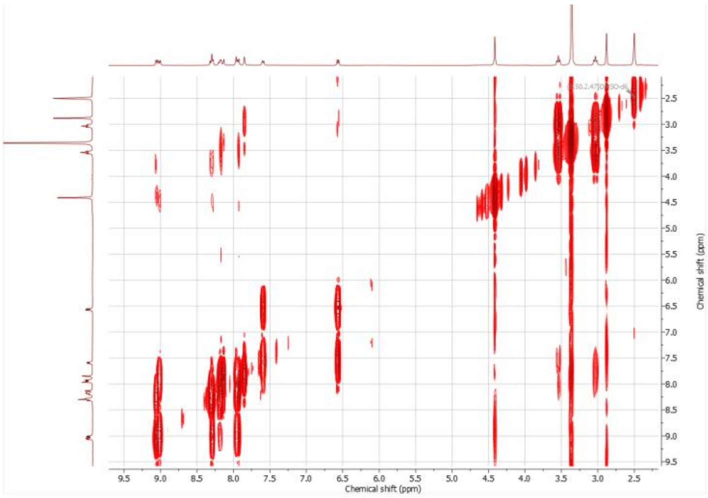

HSQC 4b

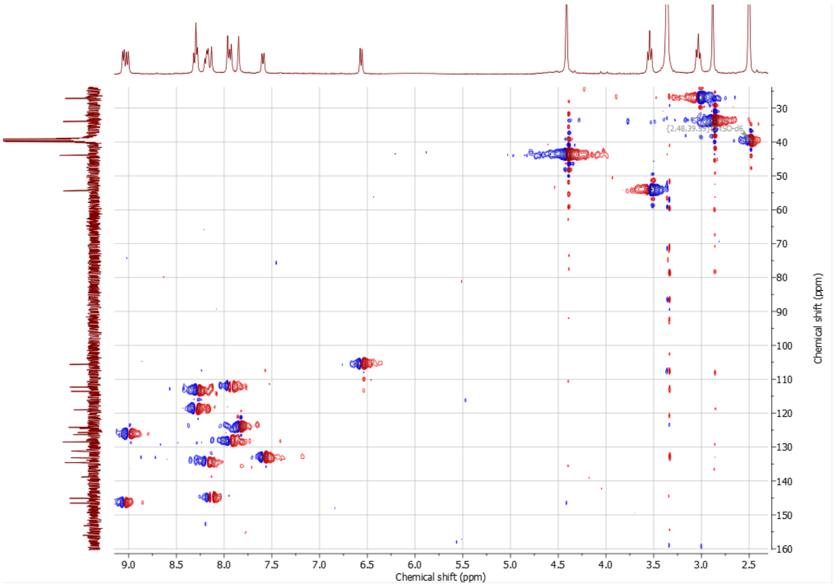

HMBC 4b

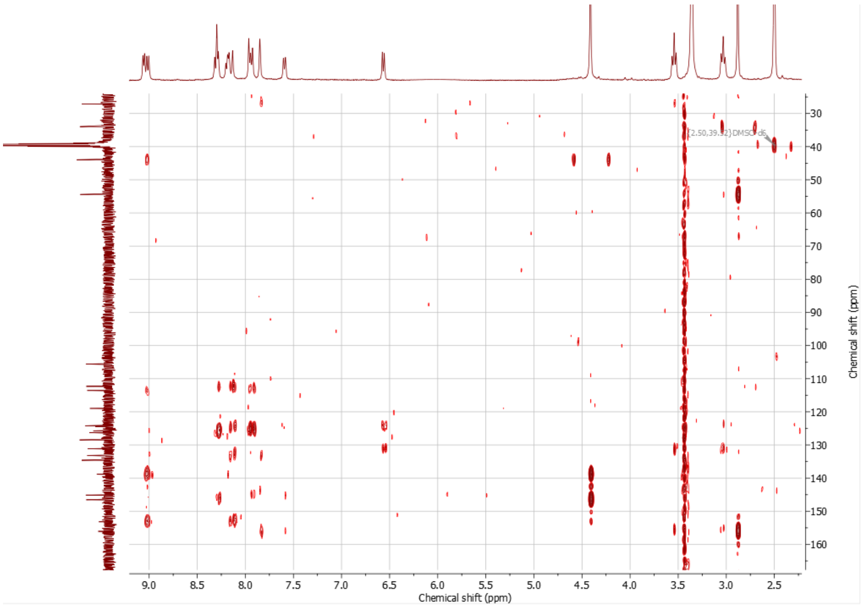

(E)-1-methyl-4-(2-(4-methyl-3,4-dihydro-2H-benzo[b][1,4]oxazin-7-yl)vinyl)quinolin-1-ium iodide (5b) in DMSO-d_6

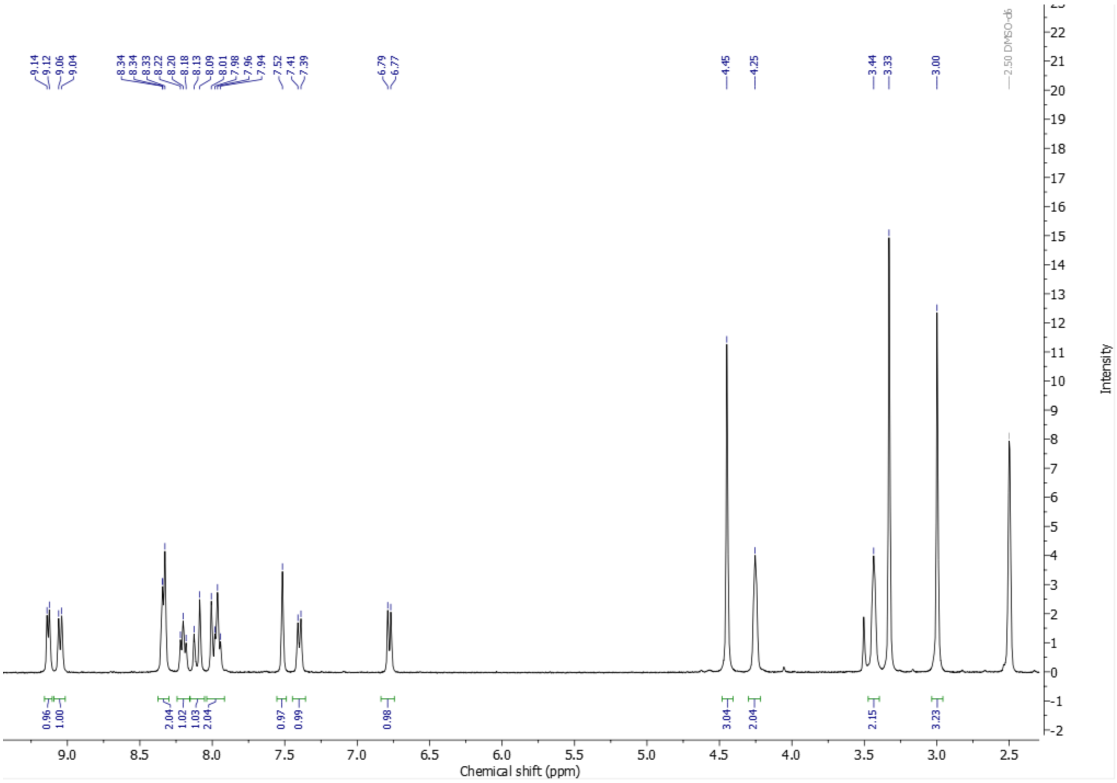

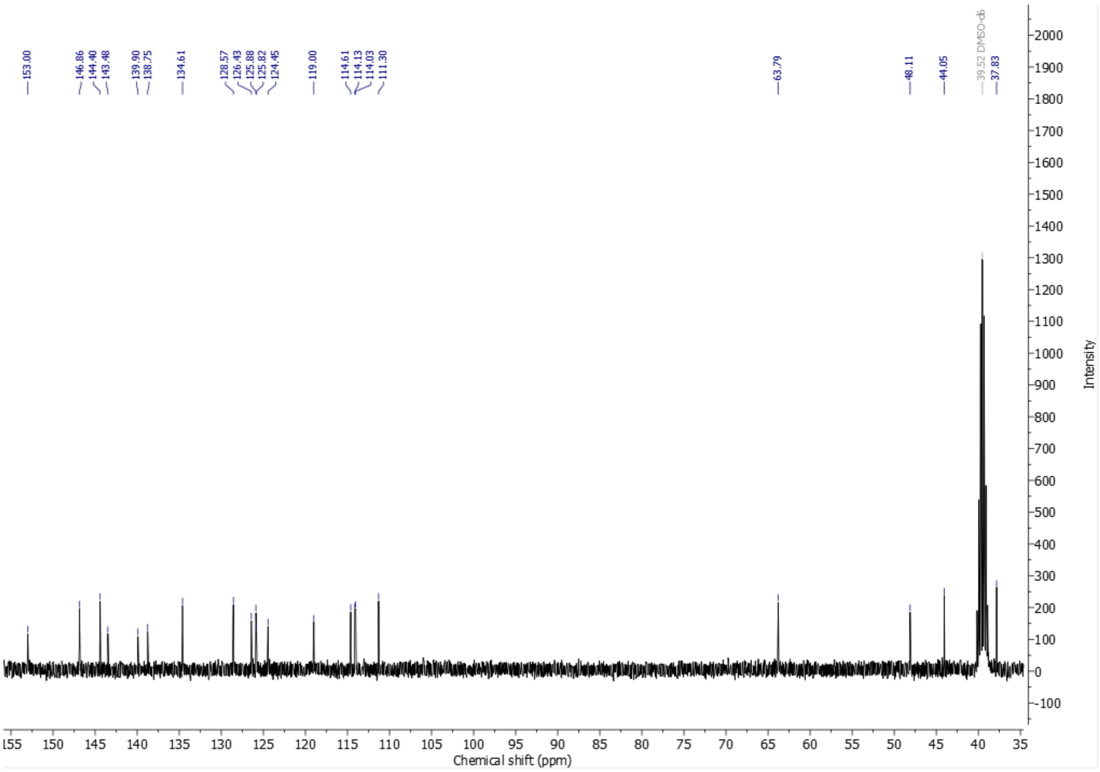
153.00
146.86
144.40
143.48
139.90
138.75
134.61
128.57
126.43
125.88
125.82
124.45
119.00
114.61
114.13
114.03
111.30
63.79
48.11
44.05
39.52 DMSO-d6
37.83
Chemical shift (ppm)
Intensity

COSY 5b

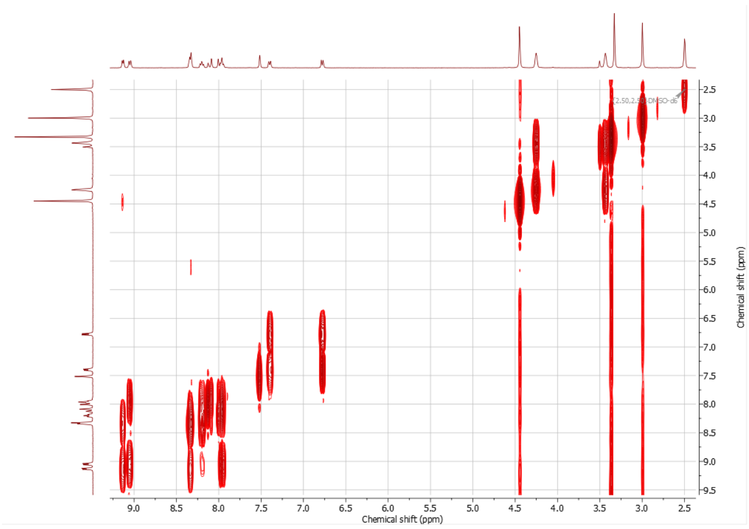

HSQC 5b

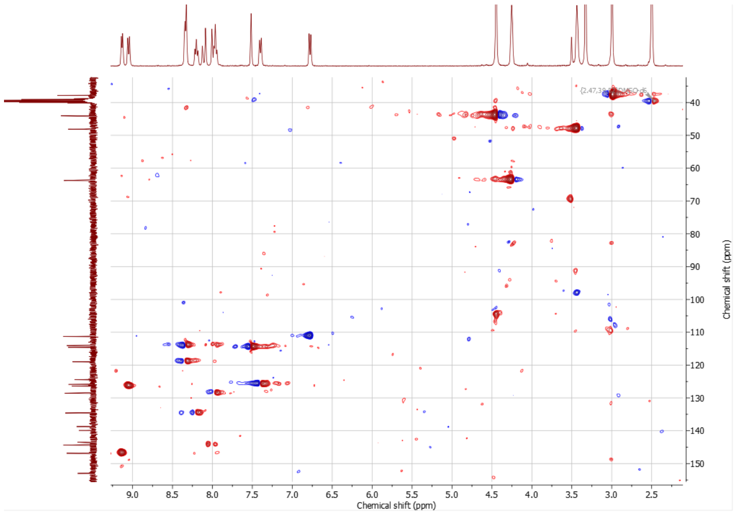

HMBC 5b

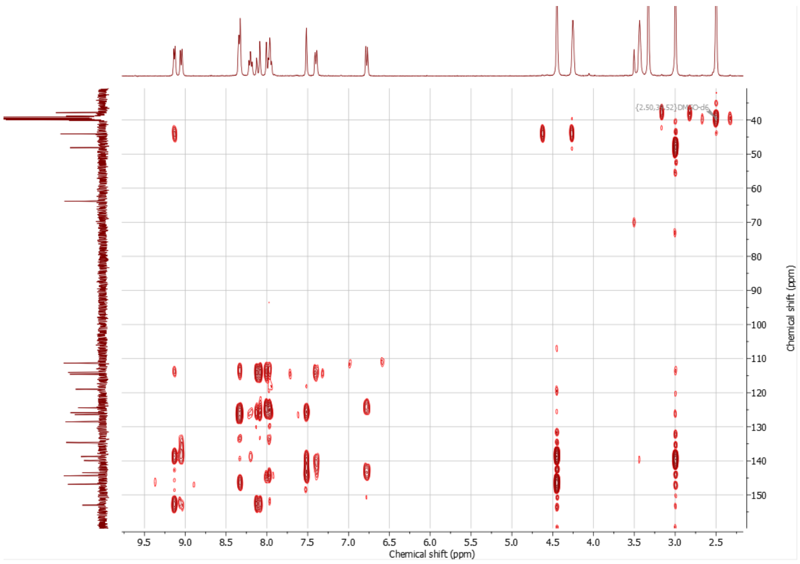

(E)-4-(4-(dimethylamino)styryl)-1-methylquinolin-1-ium iodide (6b) in DMSO-d_6

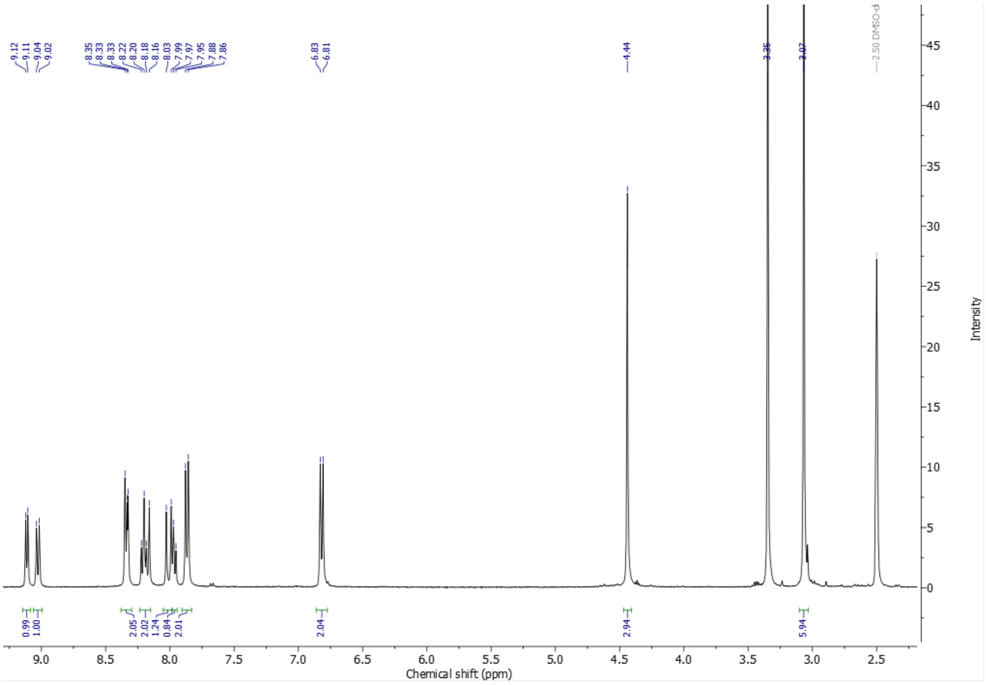

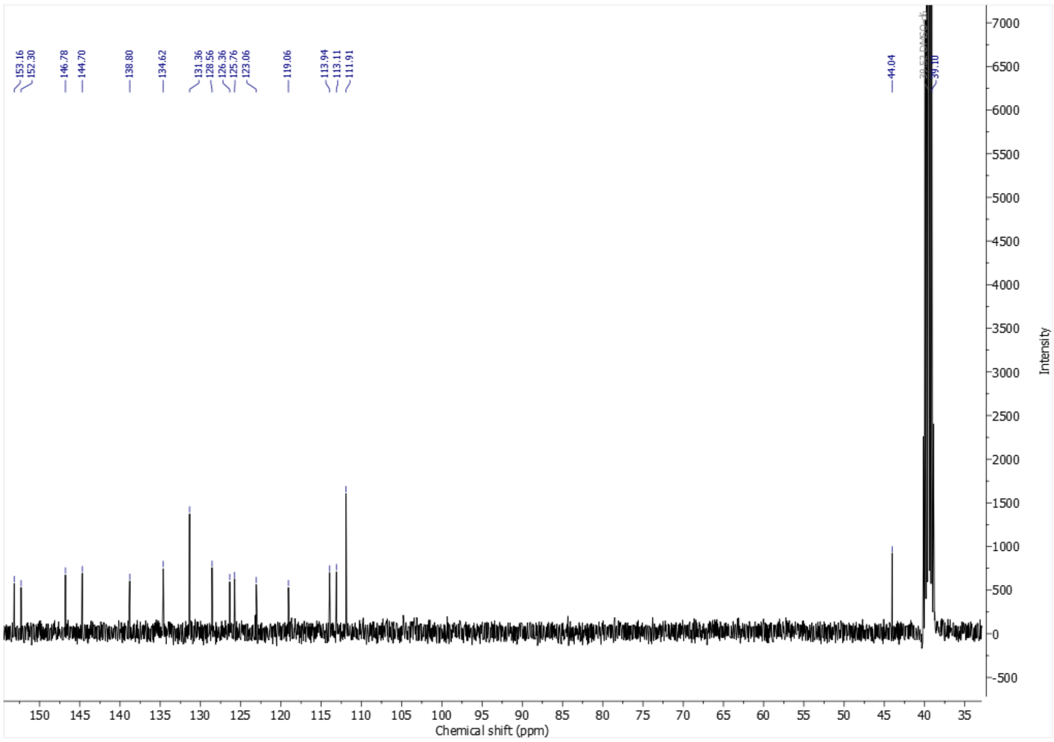

HSQC 6b

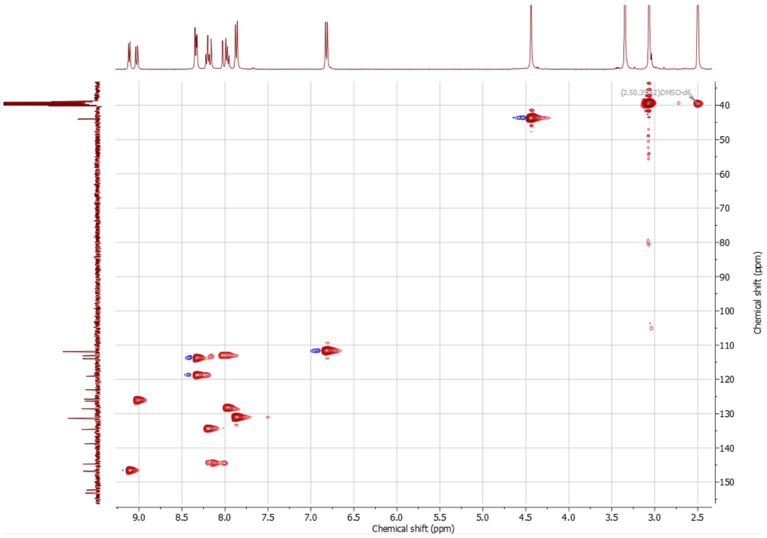

HMBC 6b

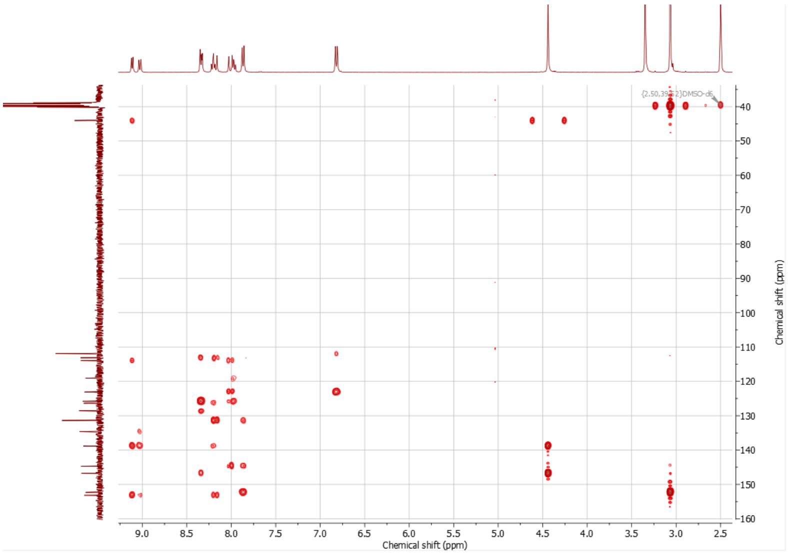

(E)-4-(4-(diphenylamino)styryl)-1-methylquinolin-1-ium iodide (7b) in DMSO-d_6

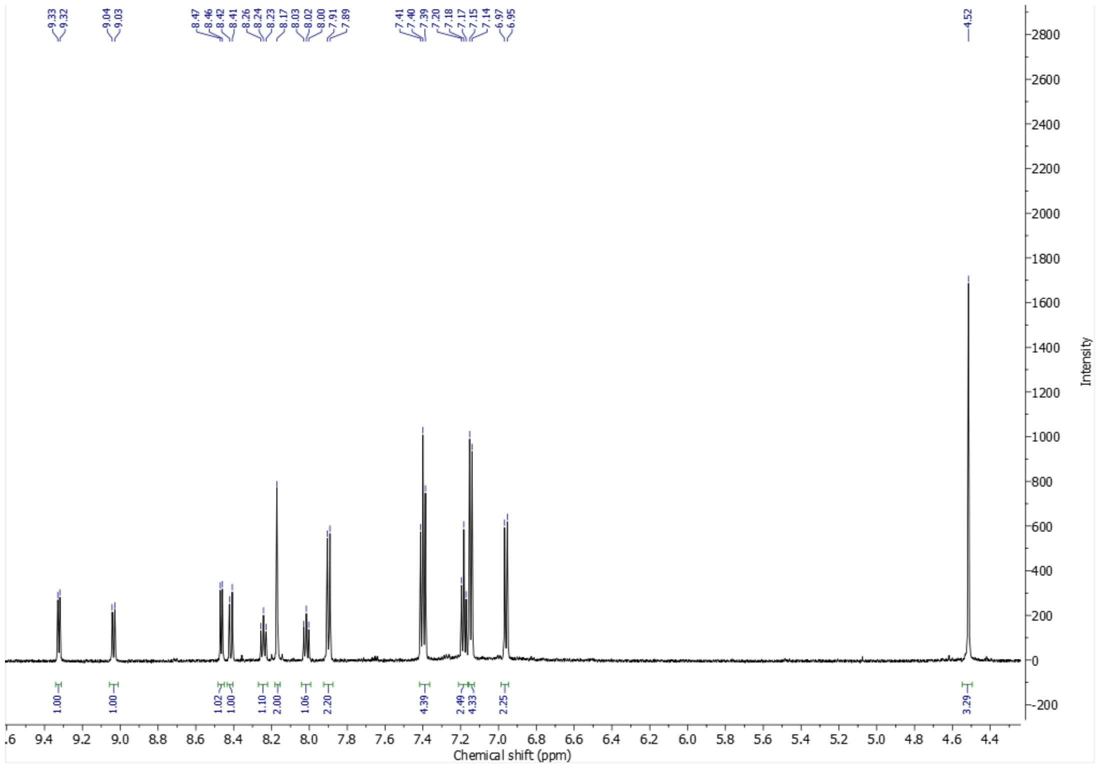

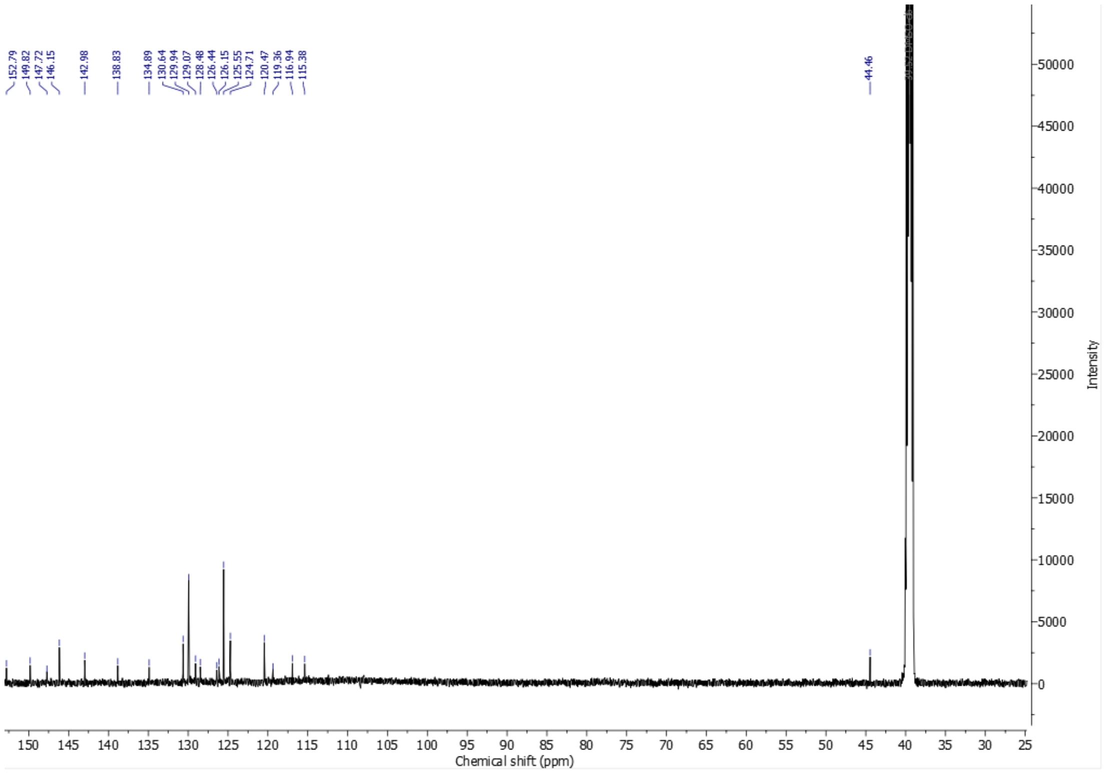

COSY 7b

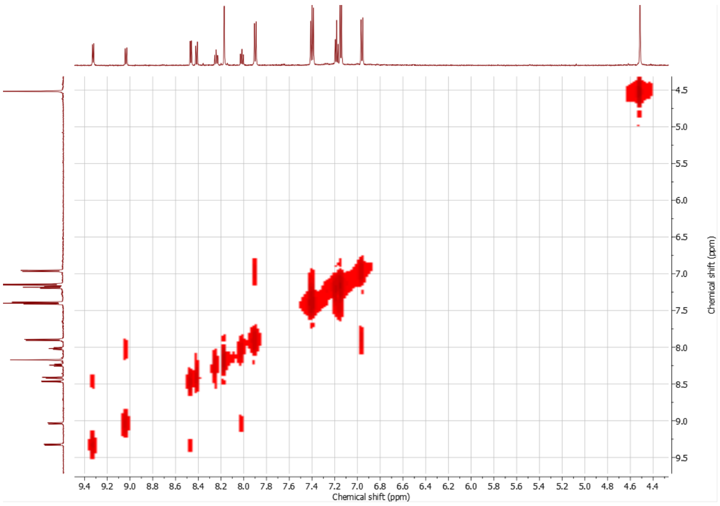

HSQC 7b

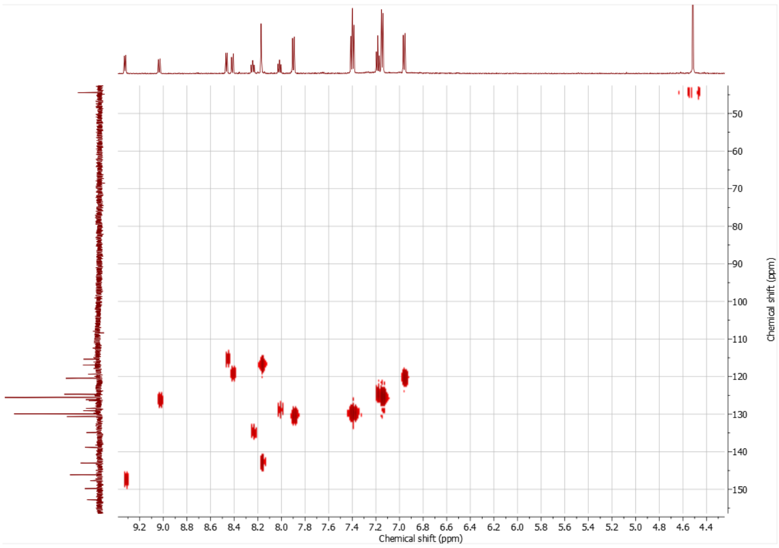

HMBC 7b

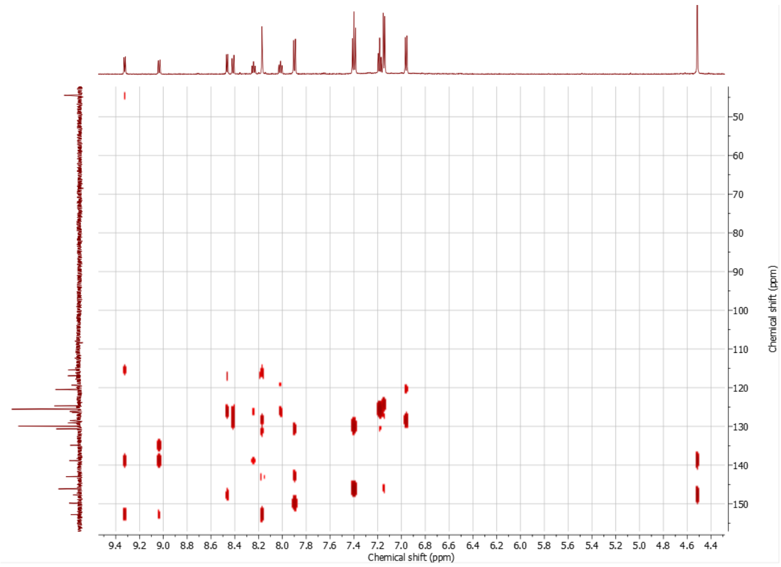

(E)-1-methyl-4-(2-(1-propyl-1,2,3,4-tetrahydroquinolin-6-yl)vinyl)quinolin-1-ium iodide (8b) in DMSO-d$_6$

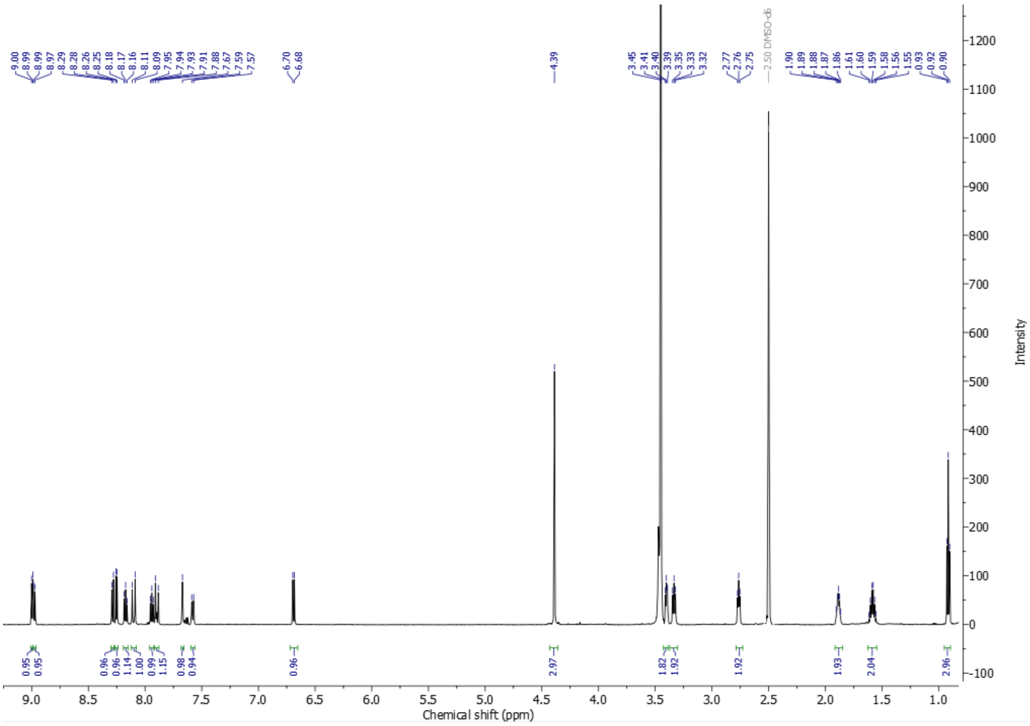

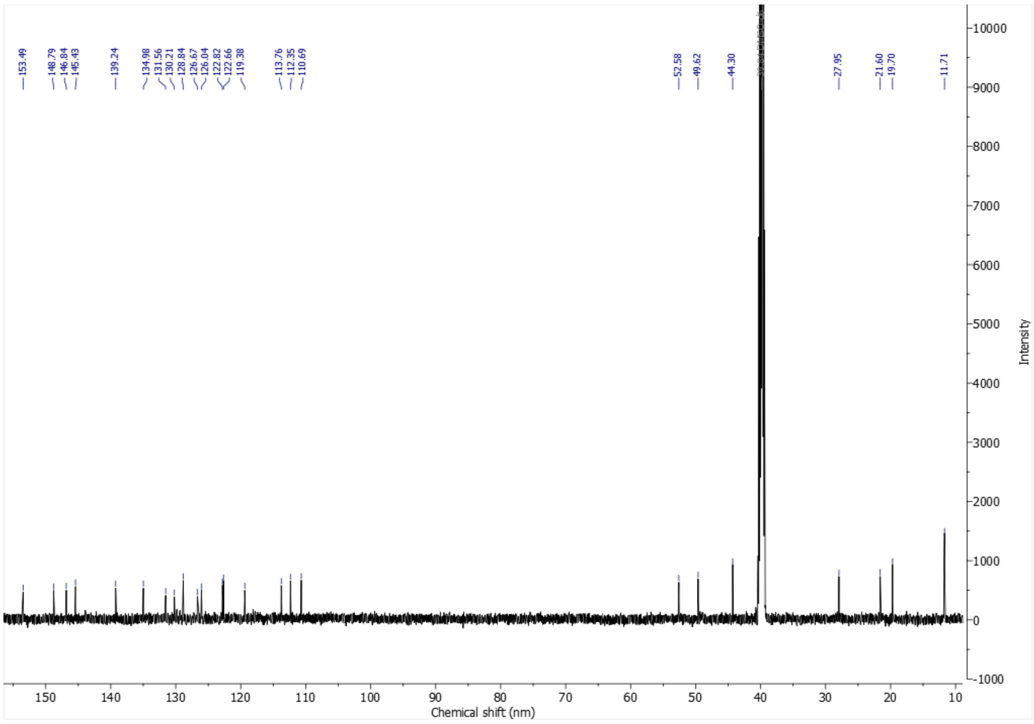

COSY 8b

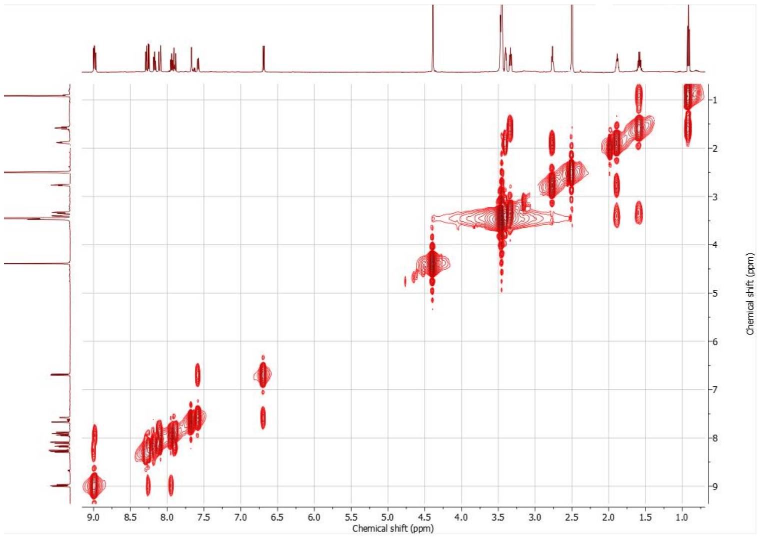

HSQC 8b

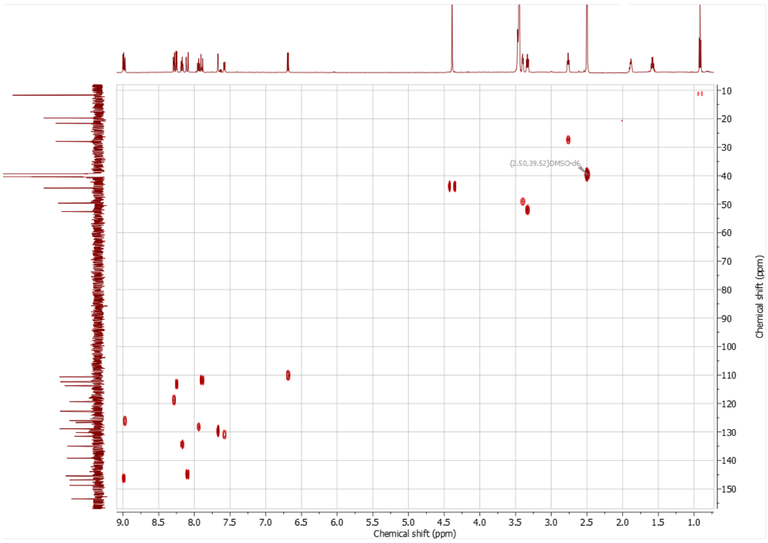

HMBC 8b

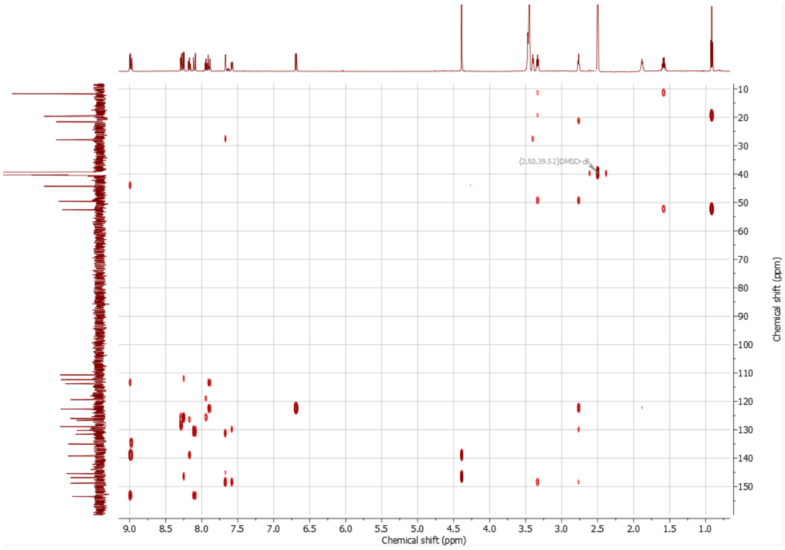

(E)-1-methyl-4-(2-(2,3,6,7-tetrahydro-1H,5H-pyrido[3,2,1-ij]quinolin-9-yl)vinyl)quinolin-1-ium iodide (9b) in DMSO-d_6

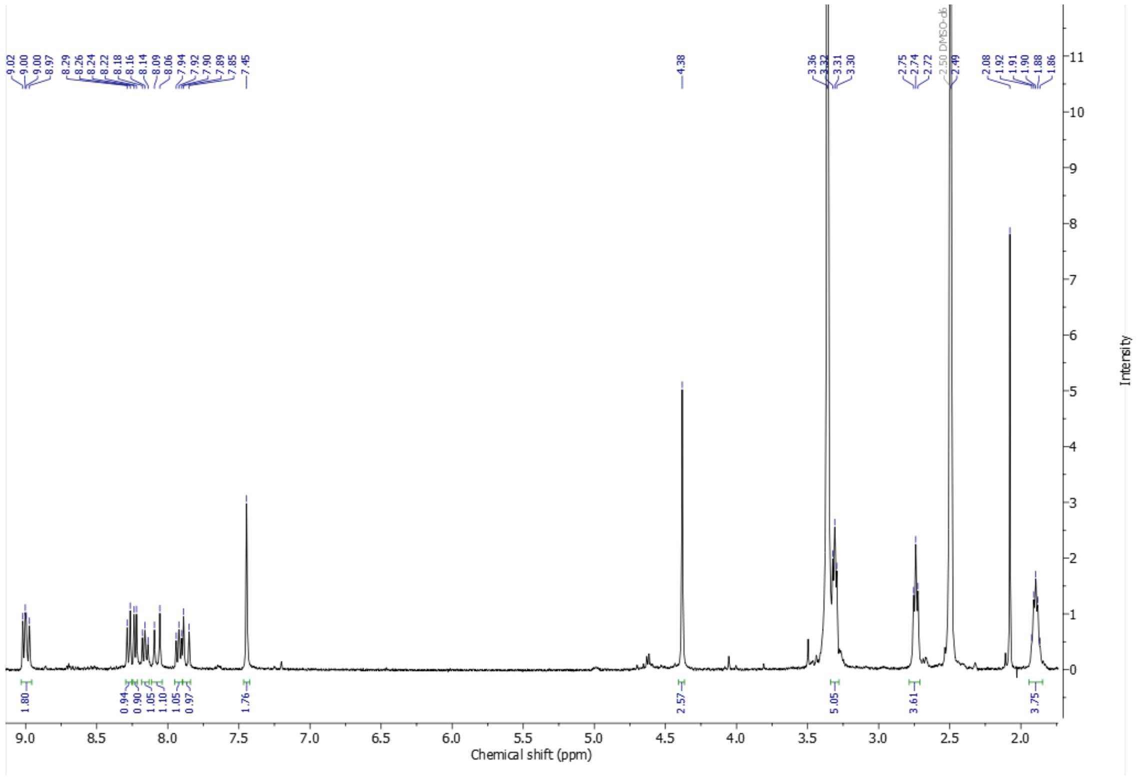

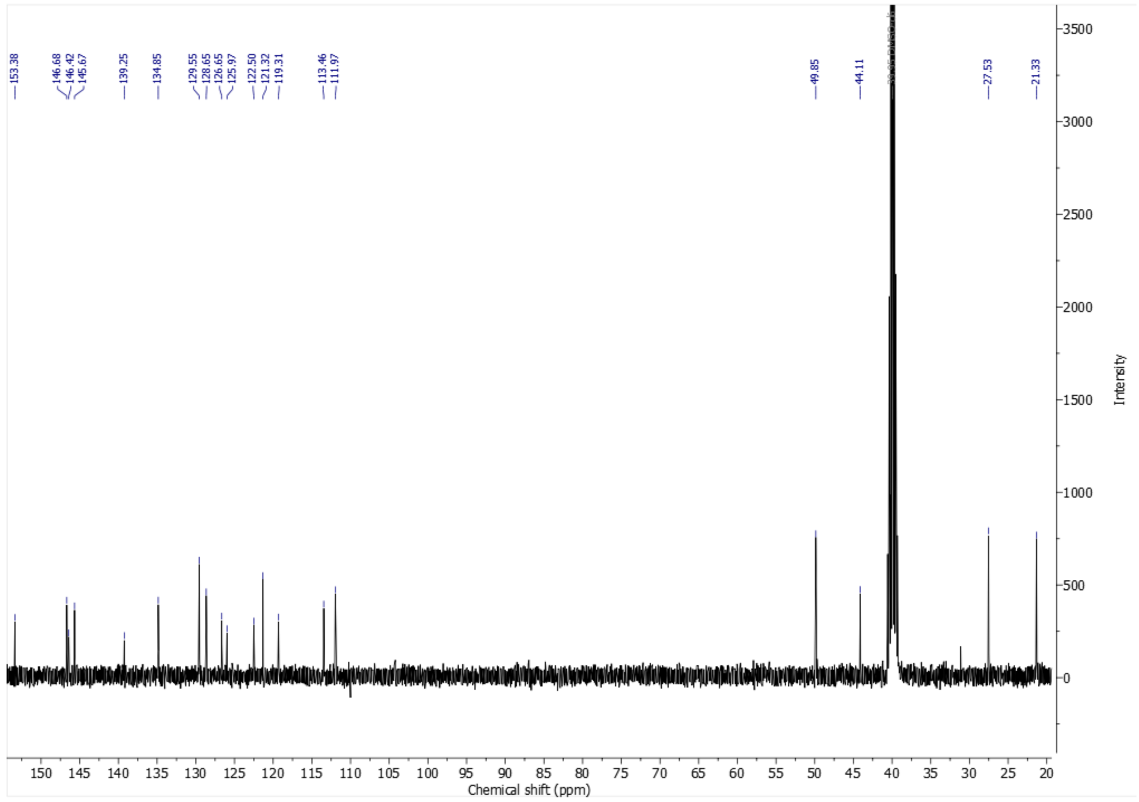

COSY 9b

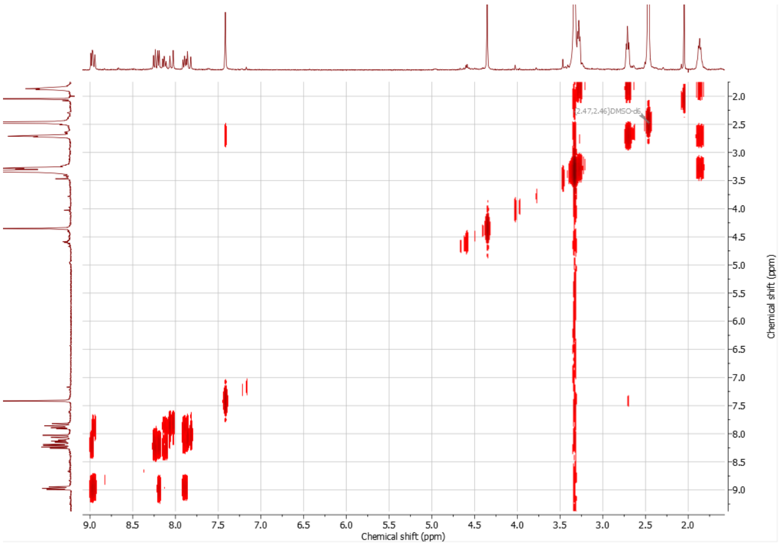

HSQC 9b

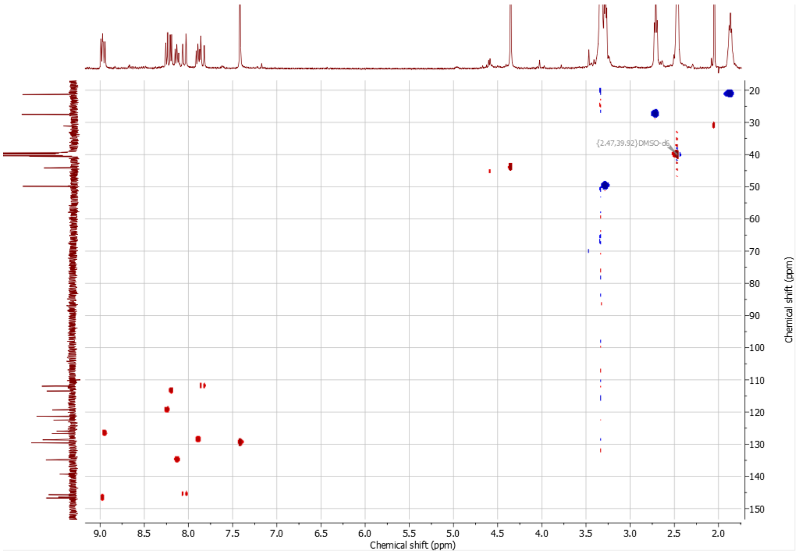

HMBC 9b

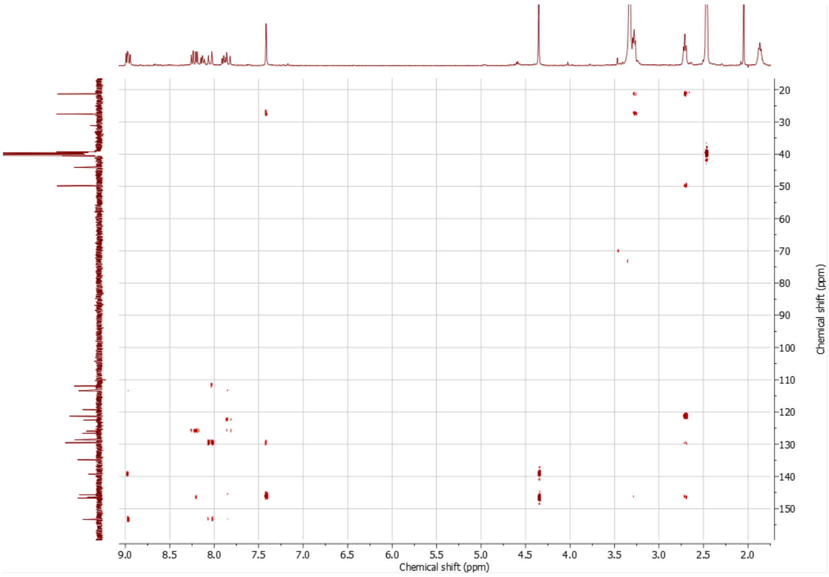

3. ESI MS studies

ESI MS spectra of **3a.**

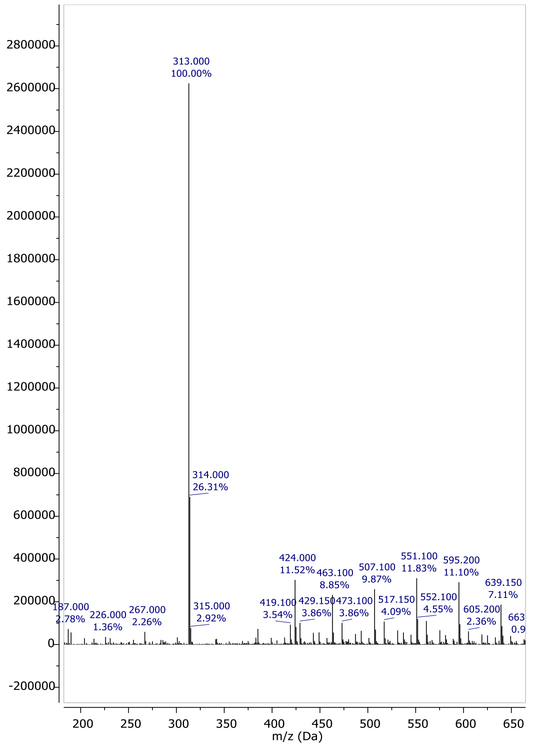

ESI MS spectra of **4a.**

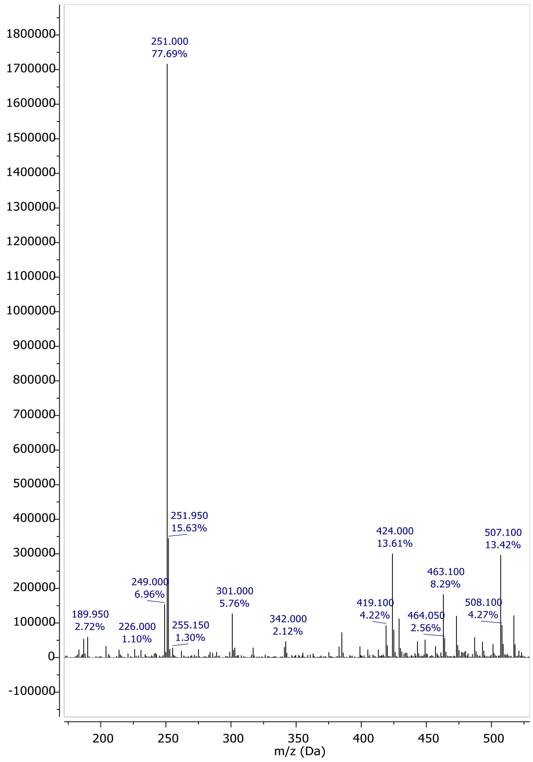

ESI MS spectra of **5a.**

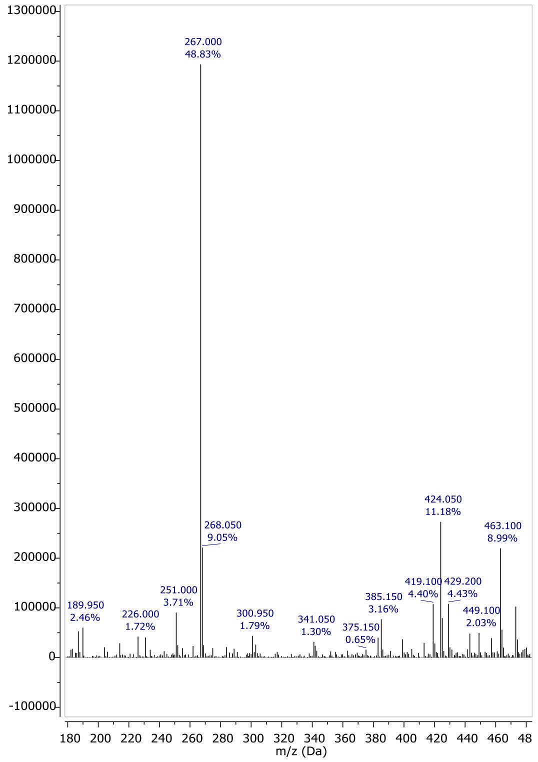

ESI MS spectra of **7a.**

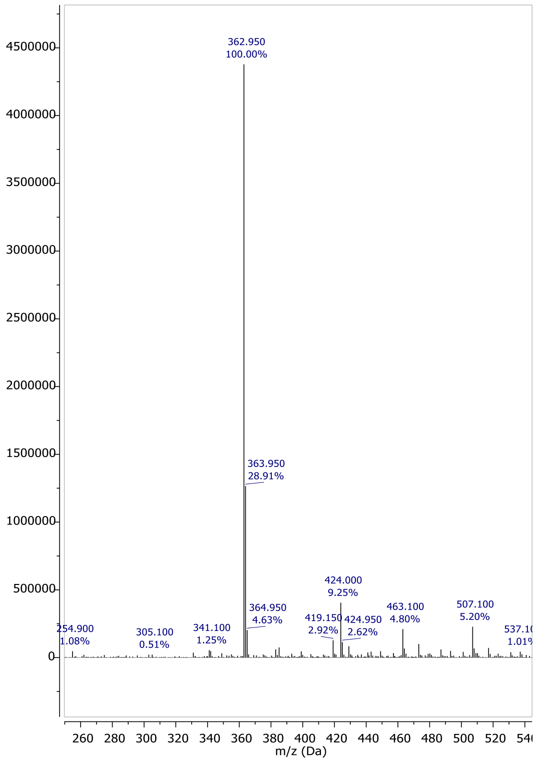

ESI MS spectra of **8a.**

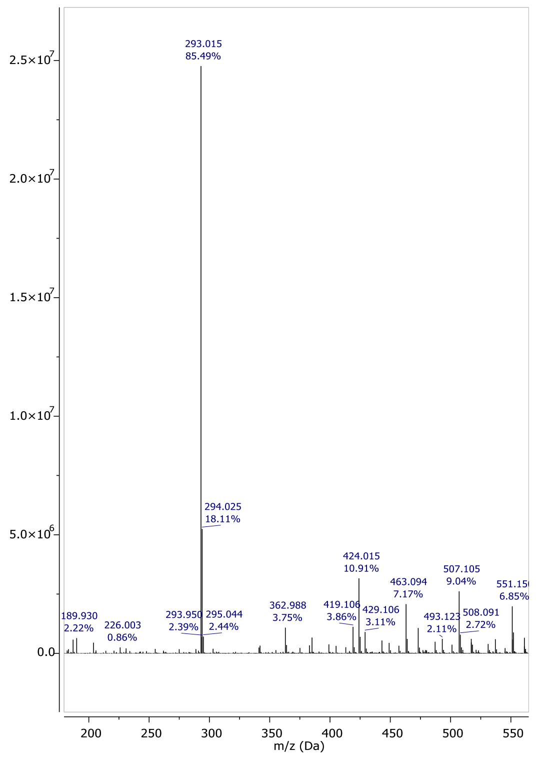

ESI MS spectra of **9a.**

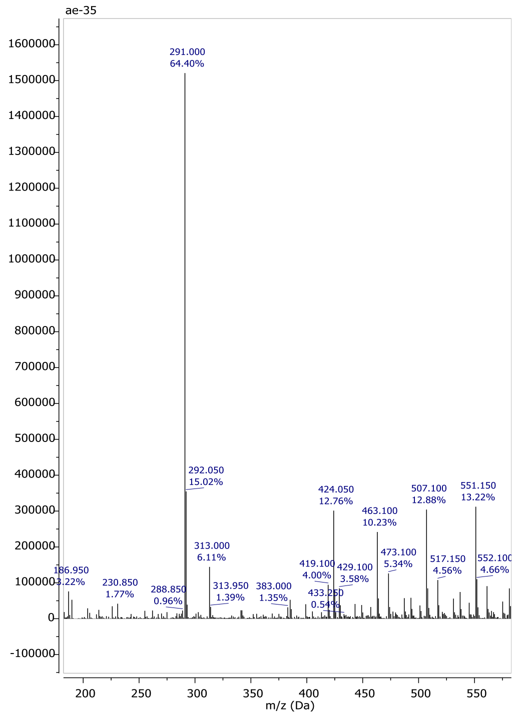

ESI MS spectra of **3b.**

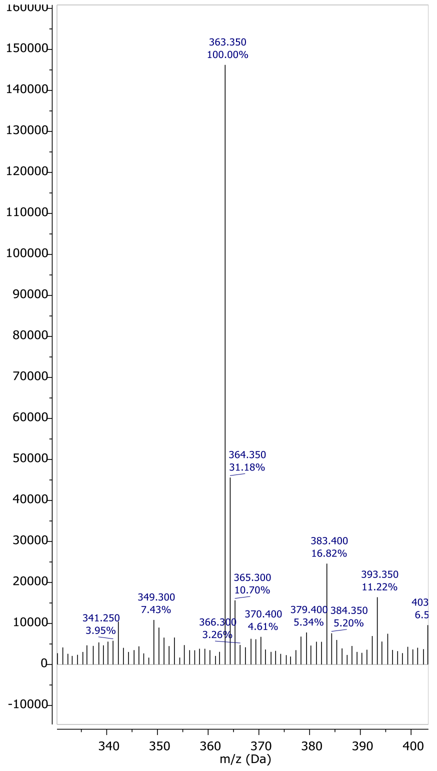

ESI MS spectra of **4b.**

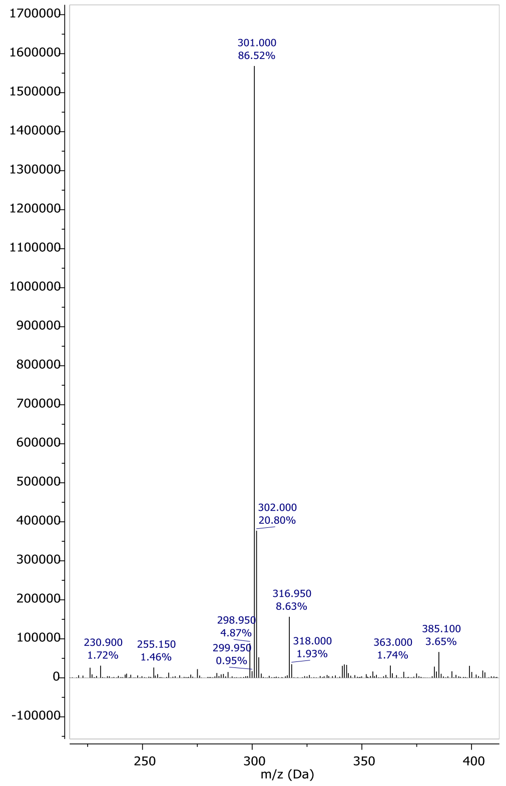

ESI MS spectra of **5b.**

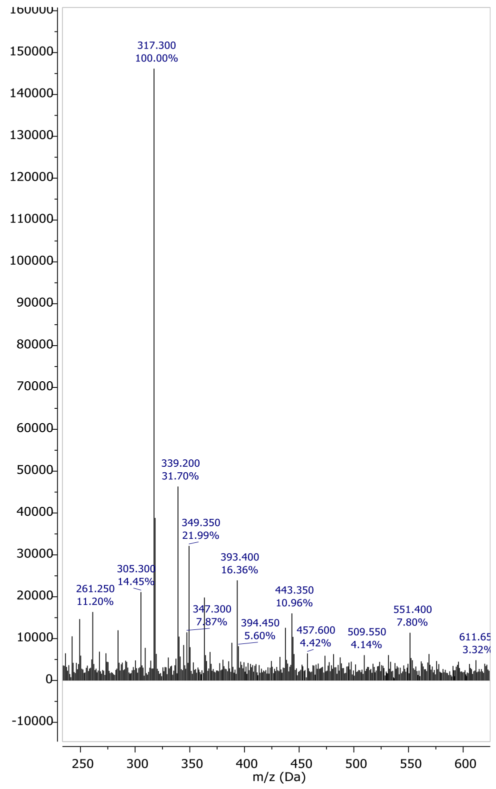

ESI MS spectra of **8b.**

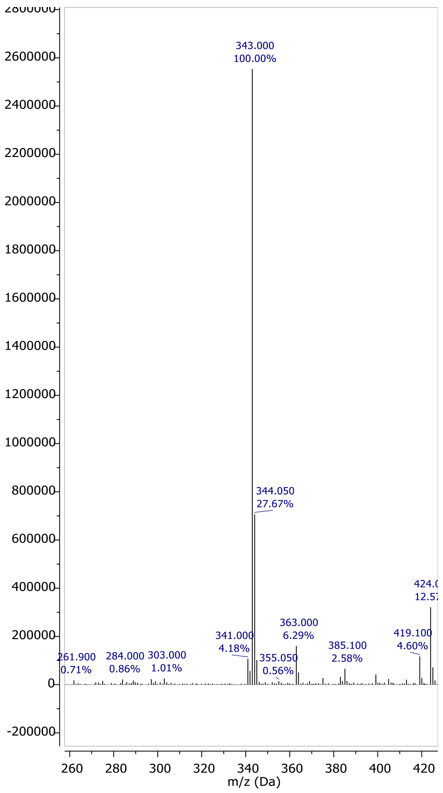

ESI MS spectra of **9b.**

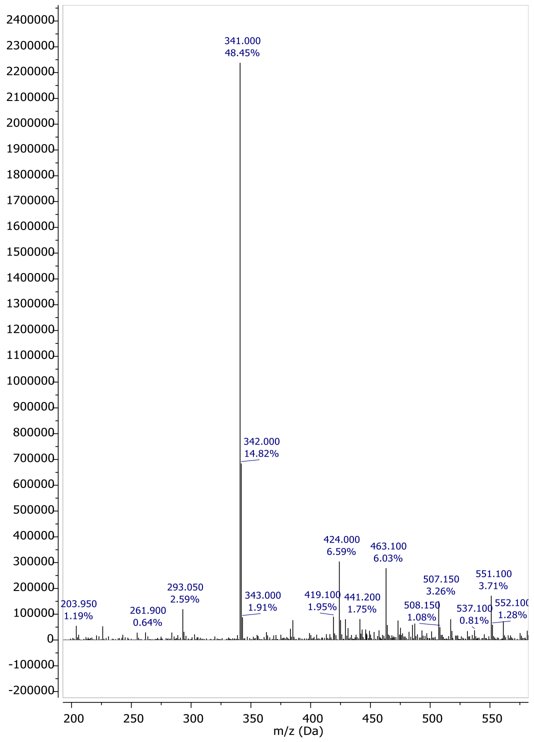

4. Optical studies

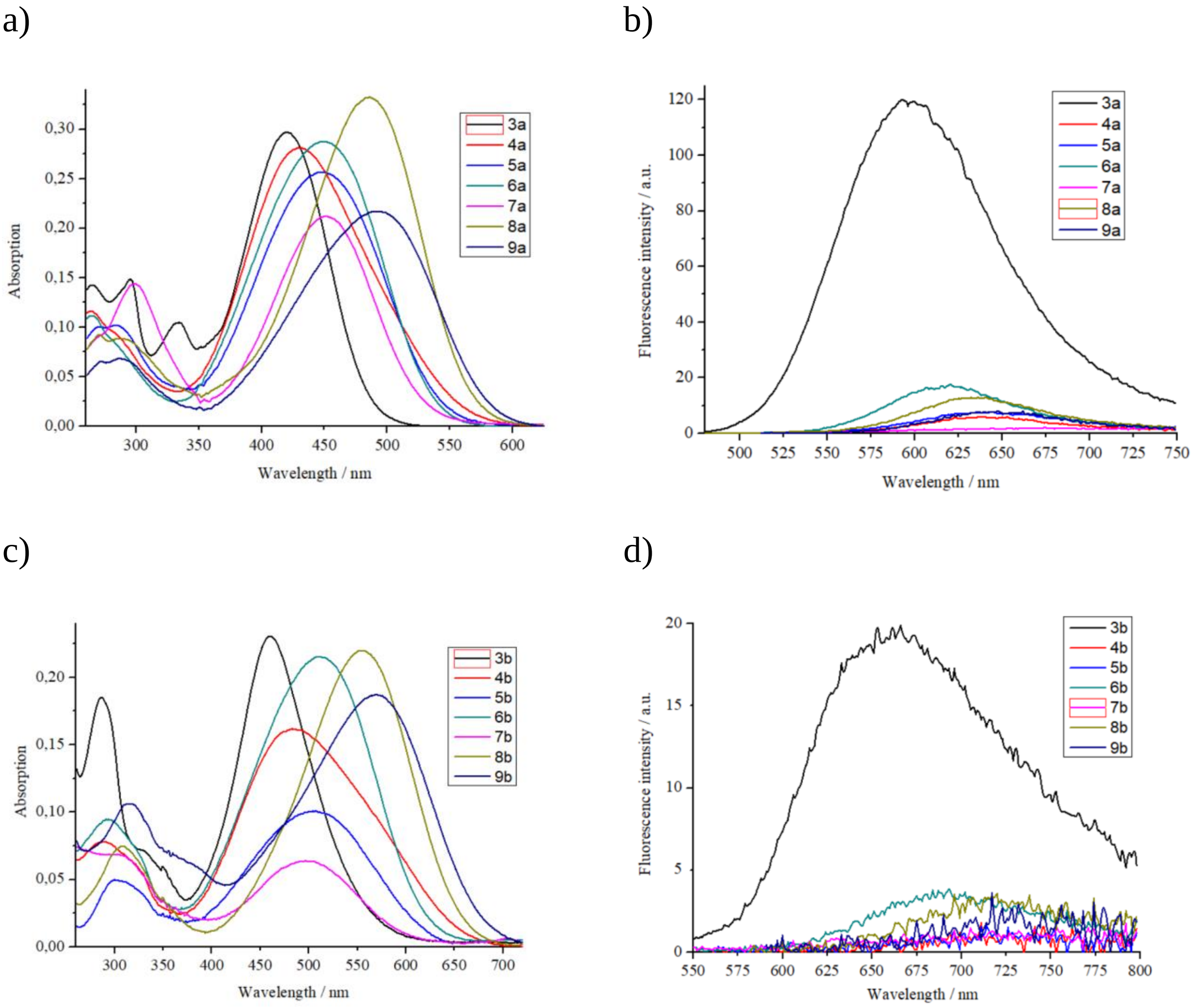

Figure S2. Absorption (a,c) and fluorescence (b,d) spectra of **3a,b-9a,b** in BPE buffer at pH=7, $C_{\mathbf{3a\text{-}9b}}=1\cdot10^{-5}$.

Table S1. Optical characteristics of dyes **3a-9a** in different solvents.

**Solvents (ε)**	**3a**			**4a**			**5a**			**6a (DASPI)**		
	φ (%)	λ_{max} (abs)	λ_{max} (fl)	φ (%)	λ_{max} (abs)	λ_{max} (fl)	φ (%)	λ_{max} (abs)	λ_{max} (fl)	φ (%)	λ_{max} (abs)	λ_{max} (fl)
Water (78,4)	1.68	420	594	0.16	432	642	0.16	451	653	0.22	450	620
Methanol (32,6)	22.6	436	591	0.18	543	683	0.25	481	632	0.63	472	616
Acetonitrile (36)	25	430	591	0.09	483	650	0.15	473	661	0.36	470	623
EtOAc (6,02)	6.95	428	588	0.18	528	663	0.72	462	613	1.02	460	605
DCM (9,08)	22.3	480	591	1.89	589	684	0.98	531	632	8.78	527	616
**Solvents (ε)**	**7a**			**8a**			**9a**					
	φ (%)	λ_{max} (abs)	λ_{max} (fl)	φ (%)	λ_{max} (abs)	λ_{max} (fl)	φ (%)	λ_{max} (abs)	λ_{max} (fl)			

Water (78,4)	0.06	451	650	0.23	486	632	0.14	491	645
Methanol (32,6)	0.29	466	700	0.48	506	635	0.3	519	640
Acetonitrile (36)	0.16	460	740	0.28	500	636	0.22	515	622
EtOAc (6,02)	1.13	458	649	1.41	491	610	0.97	500	624
DCM (9,08)	2.21	518	714	1.8	555	633	0.63	574	644

Table S2. Optical characteristics of dyes **3b-9b** in different solvents.

**Solvents (ε)**	**3b**			**4b**			**5b**			**6b**		
	φ (%)	λ_{max} (abs)	λ_{max} (fl)	φ (%)	λ_{max} (abs)	λ_{max} (fl)	φ (%)	λ_{max} (abs)	λ_{max} (fl)	φ (%)	λ_{max} (abs)	λ_{max} (fl)
Water (78,4)	0.12	462	658	0.03	484	726	0.05	505	718	0.05	510	694
Methanol (32,6)	6.44	484	654	0.09	566	713	0.13	547	709	0.18	543	683
Acetonitrile (36)	6.39	483	667	-	563	-	-	539	-	0.09	536	698
EtOAc (6,02)	3.74	479	649	0.25	545	698	0.21	533	702	0.18	528	663
DCM (9,08)	39.5	534	646	0.28	620	717	0.44	595	715	1.89	589	684

**Solvents (ε)**	**7b**			**8b**			**9b**		
	φ (%)	λ_{max} (abs)	λ_{max} (fl)	φ (%)	λ_{max} (abs)	λ_{max} (fl)	φ (%)	λ_{max} (abs)	λ_{max} (fl)
Water (78,4)	0.04	497	728	0.07	554	711	0.05	569	730
Methanol (32,6)	-	521	-	0.19	579	705	0.22	597	703
Acetonitrile (36)	-	515	-	0.11	575	731	0.18	594	622
EtOAc (6,02)	-	509	-	0.49	561	678	0.74	571	699
DCM (9,08)	-	576	-	0.55	626	709	1.52	645	605

Table S3. Optical characteristics of dyes **3a,b-9a,b** in glycerol.

**Compound**	**λ^{abs} nm**	**λ^{fl} nm**	**φ^{fl} (%)**
**3a**	439	591	42.1
**4a**	488	647	15.2
**5a**	483	636	5.82
**6a (DASPI)**	481	617	13.4

**7a**	471	653	4.61
**8a**	513	626	18.4
**9a**	524	642	5.54
**3b**	488	647	15.2
**4b**	576	701	0.89
**5b**	552	718	1.12
**6b**	488	647	15.2
**7b**	526	709	1.7
**8b**	588	708	1.9
**9b**	607	710	1.28

a) b)

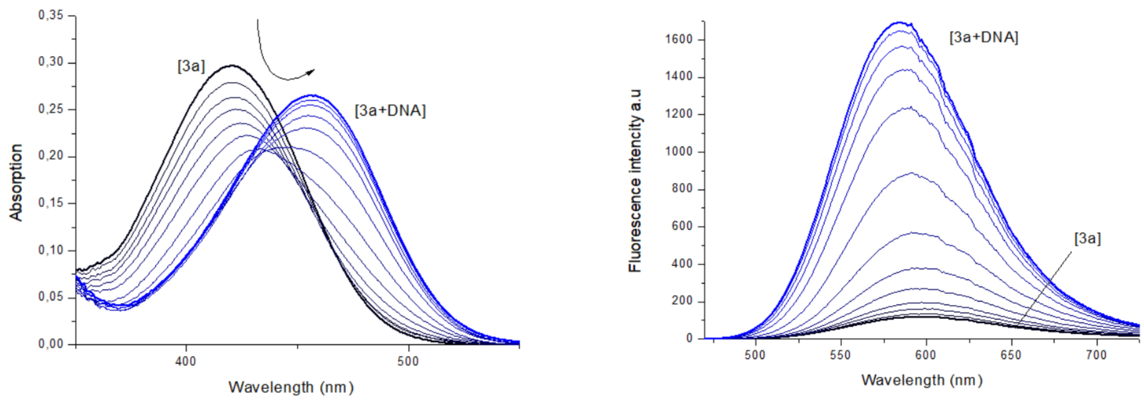

Figure S3. Spectrophotometric (a) and fluorometric (b) titration of dye **3a** with ds-DNA solution; pH=7, $C_{\mathbf{3a}}$=1·10^{-5} M, $C_{ds\text{-}DNA}$=0-0.5·10^{-3} M b.p.

a) b)

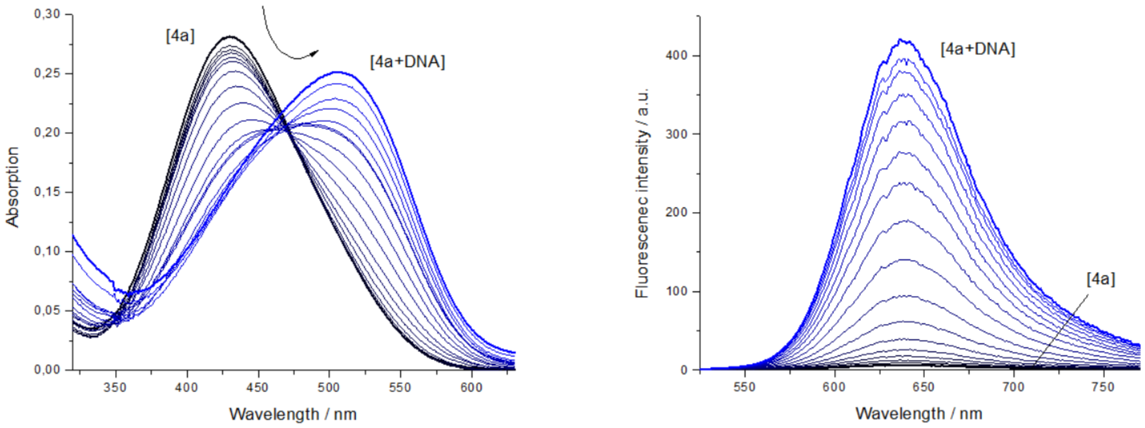

Figure S4. Spectrophotometric (a) and fluorometric (b) titration of dye **4a** with ds-DNA solution; pH=7, $C_{\mathbf{4a}}$=$1{\cdot}10^{-5}$ M, $C_{ds\text{-}DNA}$=0-$0.7{\cdot}10^{-3}$ M b.p.

a) b)

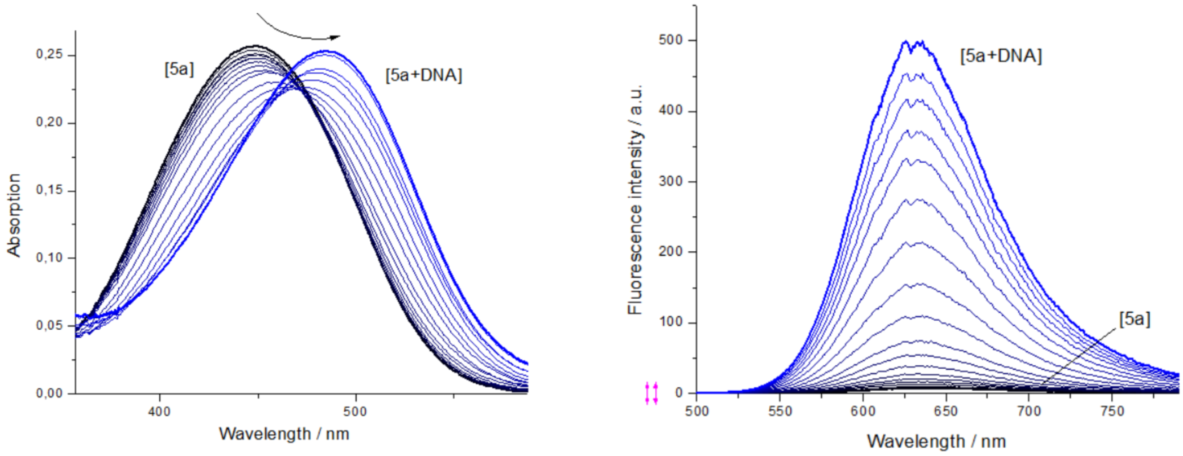

Figure S5. Spectrophotometric (a) and fluorometric (b) titration of dye **5a** with ds-DNA solution; pH=7, $C_{\mathbf{5a}}$=$1{\cdot}10^{-5}$ M, $C_{ds\text{-}DNA}$=0-$0.65{\cdot}10^{-3}$ M b.p.

a) b)

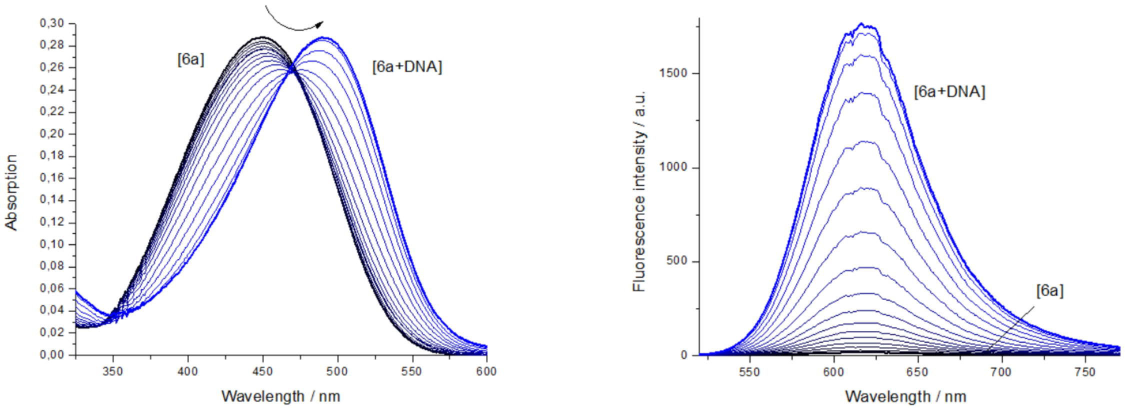

Figure S6. Spectrophotometric (a) and fluorometric (b) titration of dye **6a** (DASPI) with ds-DNA solution; pH=7, $C_{\mathbf{6a}}$=$1{\cdot}10^{-5}$ M, $C_{ds\text{-}DNA}$=0-$0.7{\cdot}10^{-3}$ M b.p.

a) b)

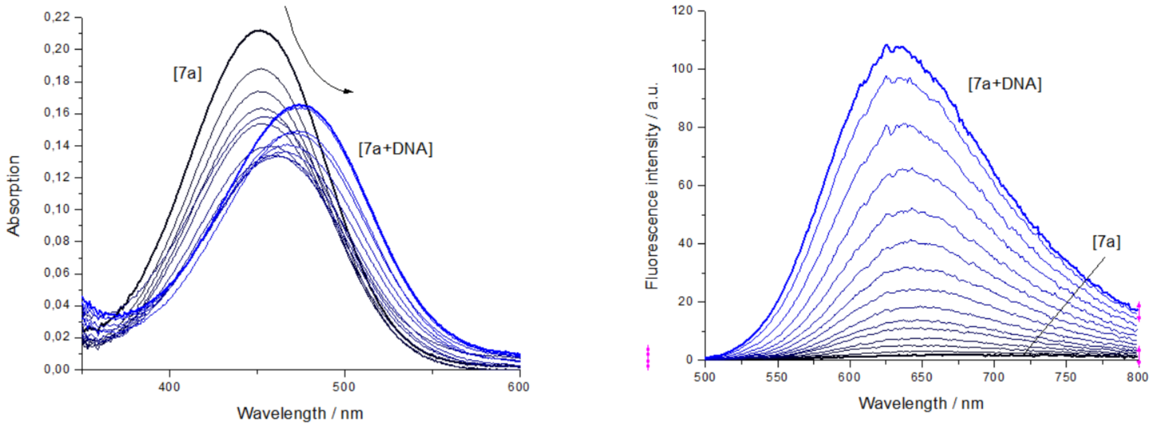

Figure S7. Spectrophotometric (a) and fluorometric (b) titration of dye **7a** with ds-DNA solution; pH=7, $C_{\mathbf{7a}}=1\cdot10^{-5}$ M, $C_{ds\text{-}DNA}=0\text{-}0.65\cdot10^{-3}$ M b.p.

a) b)

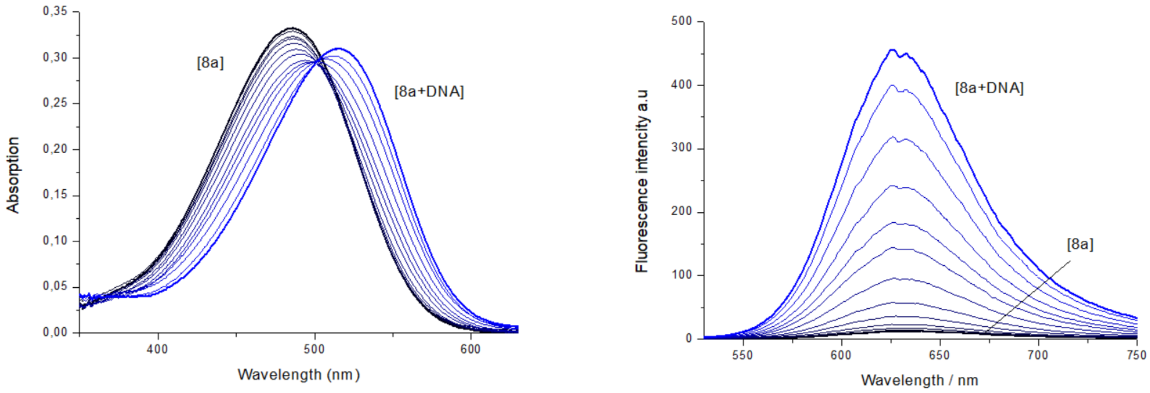

Figure S8. Spectrophotometric (a) and fluorometric (b) titration of dye **8a** with ds-DNA solution; pH=7, $C_{\mathbf{8a}}=1\cdot10^{-5}$ M, $C_{ds\text{-}DNA}=0\text{-}0.7\cdot10^{-3}$ M b.p.

a) b)

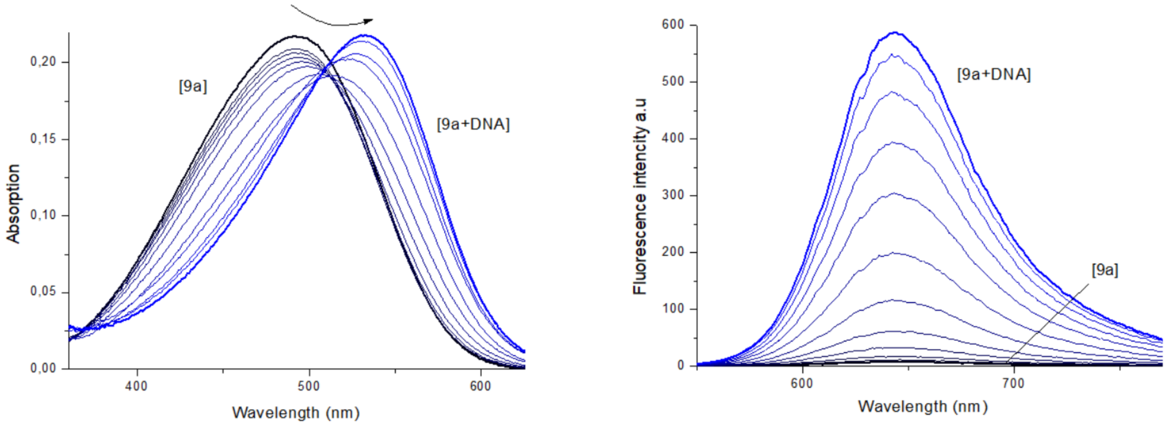

Figure S9. Spectrophotometric (a) and fluorometric (b) titration of dye **9a** with ds-DNA solution; pH=7, $C_{\mathbf{9a}}=1\cdot10^{-5}$ M, $C_{ds\text{-}DNA}=0\text{-}0.73\cdot10^{-3}$ M b.p.

a) b)

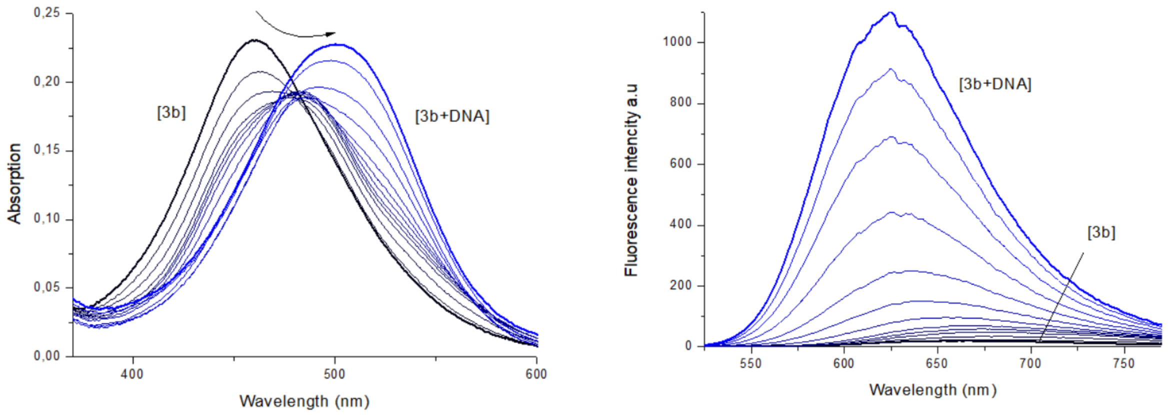

Figure S10. Spectrophotometric (a) and fluorometric (b) titration of dye **3b** with ds-DNA solution; pH=7, $C_{\mathbf{3b}}=1\cdot10^{-5}$ M, $C_{ds\text{-}DNA}=0\text{-}0.66\cdot10^{-3}$ M b.p.

a) b)

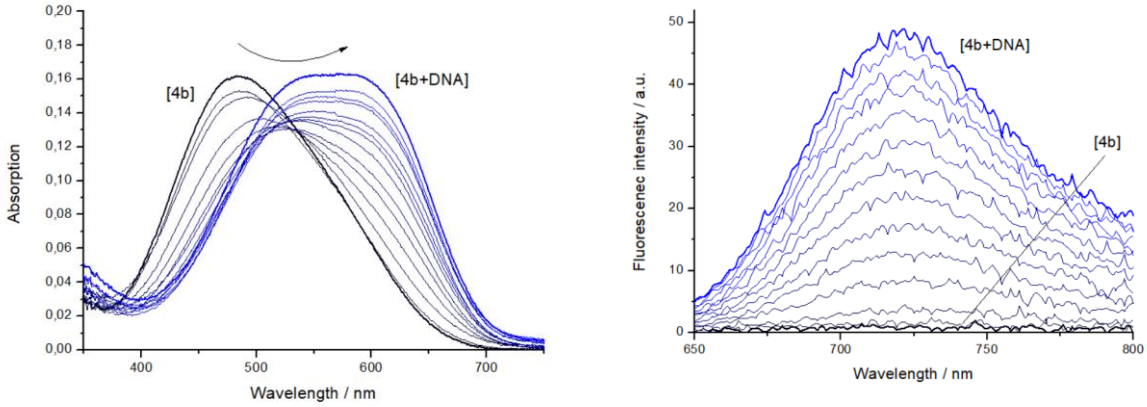

Figure S11. Spectrophotometric (a) and fluorometric (b) titration of dye **4b** with ds-DNA solution; pH=7, $C_{\mathbf{4b}}=1\cdot10^{-5}$ M, $C_{ds\text{-}DNA}=0\text{-}0.64\cdot10^{-3}$ M b.p.

a) b)

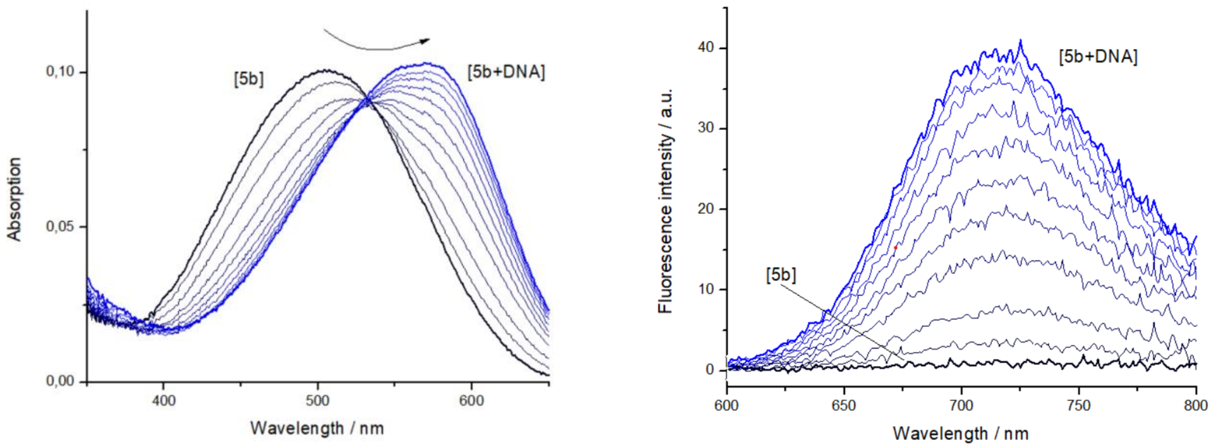

Figure S12. Spectrophotometric (a) and fluorometric (b) titration of dye **5b** with ds-DNA solution; pH=7, $C_{\mathbf{5b}}=1\cdot10^{-5}$ M, $C_{ds\text{-}DNA}=0\text{-}0.12\cdot10^{-3}$ M b.p.

a) b)

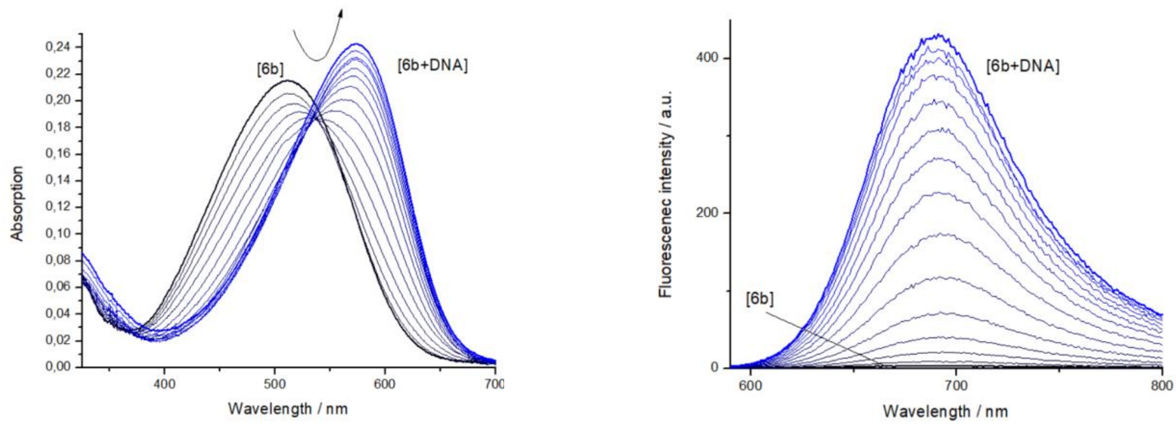

Figure S13. Spectrophotometric (a) and fluorometric (b) titration of dye **6b** with ds-DNA solution; pH=7, $C_{\mathbf{6b}}=1\cdot10^{-5}$ M, $C_{ds\text{-}DNA}=0\text{-}0.7\cdot10^{-3}$ M b.p.

a) b)

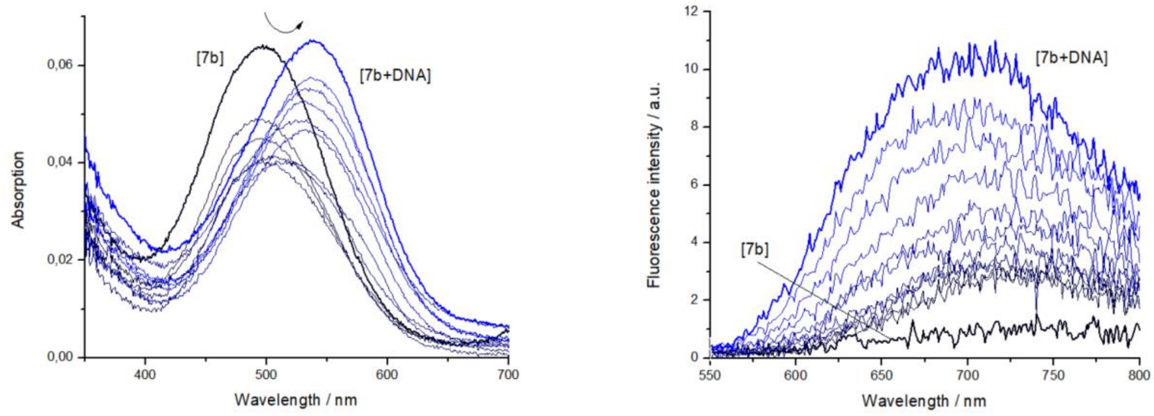

Figure S14. Spectrophotometric (a) and fluorometric (b) titration of dye **7b** with ds-DNA solution; pH=7, $C_{7b}=1\cdot10^{-5}$ M, $C_{ds\text{-}DNA}=0\text{-}0.78\cdot10^{-3}$ M b.p.

a) b)

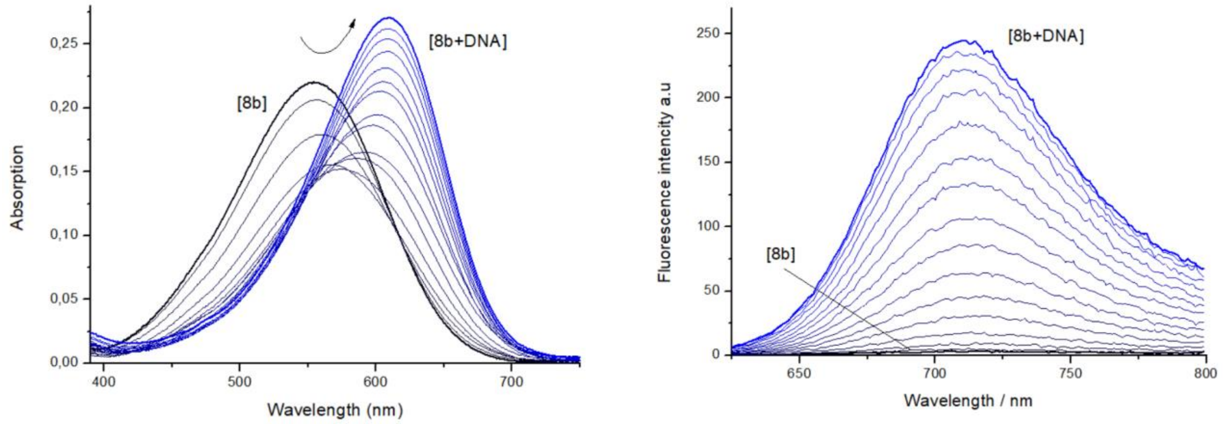

Figure S15. Spectrophotometric (a) and fluorometric (b) titration of dye **8b** with ds-DNA solution; pH=7, $C_{\mathbf{8b}}=1\cdot10^{-5}$ M, $C_{ds\text{-}DNA}=0\text{-}0.74\cdot10^{-3}$ M b.p.

a) b)

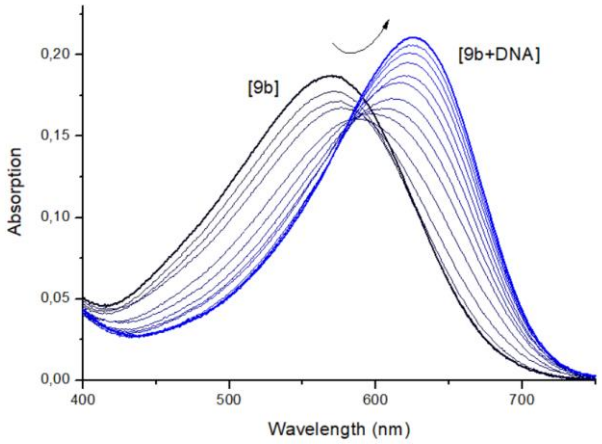

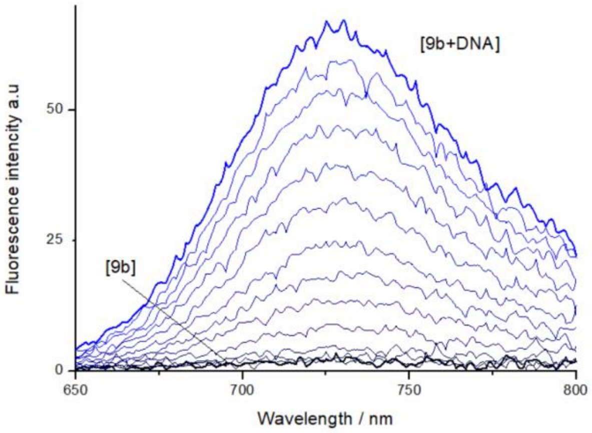

Figure S16. Spectrophotometric (a) and fluorometric (b) titration of dye **9b** with ds-DNA solution; pH=7, $C_{9b}=1\cdot10^{-5}$ M, $C_{ds\text{-}DNA}=0\text{-}0.14\cdot10^{-3}$ M b.p.

a) b)

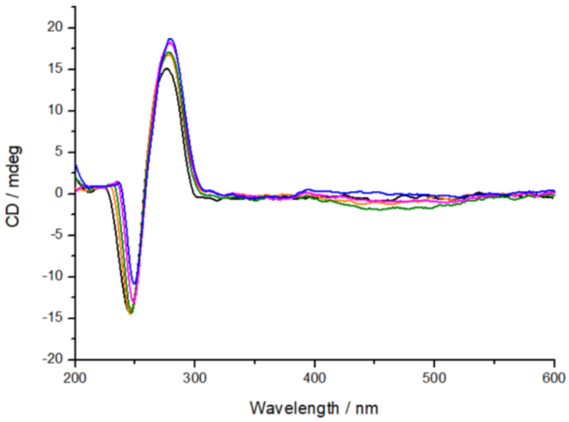

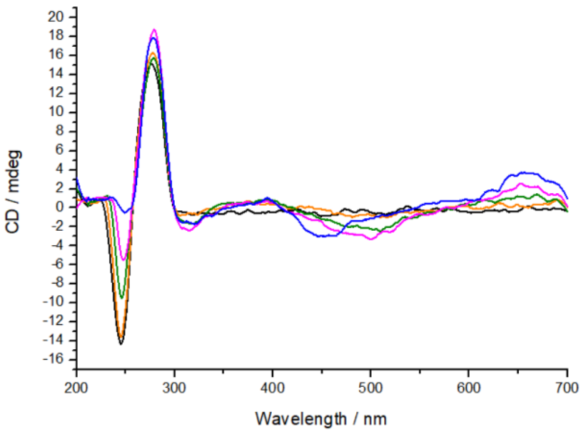

Figure S17. Circular dichroism spectra of ct-DNA (C_{DNA} = 0.1 mM b.p.) in the absence and presence of styryl dyes **4a** (a) and **4b** (b) at different LDR C_{Dye}/C_{DNA}: 0 (black); 0.1 (orange); 0.3 (green); 0.6 (magenta); 1 (blue).

a) b)

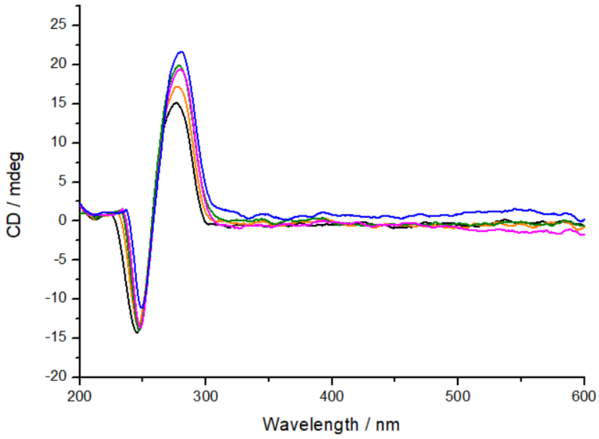

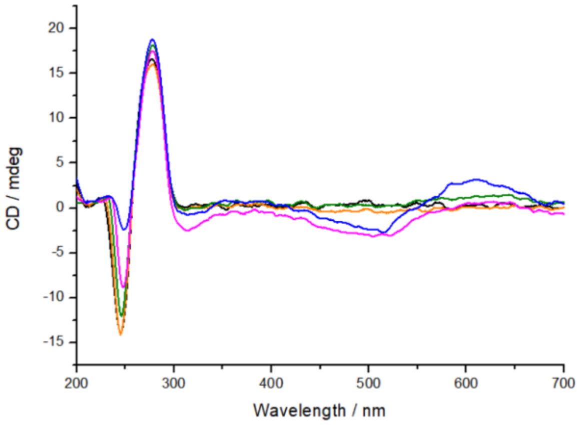

Figure S18. Circular dichroism spectra of ct-DNA (C_{DNA} = 0.1 mM b.p.) in the absence and presence of styryl dyes **5a** (a) and **5b** (b) at different LDR C_{Dye}/C_{DNA}: 0 (black); 0.1 (orange); 0.3 (green); 0.6 (magenta); 1 (blue).

a)

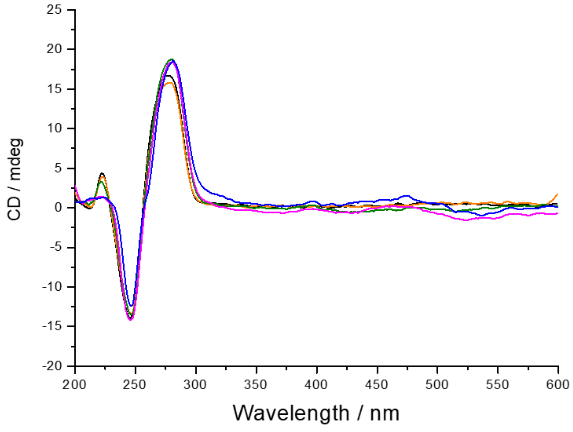

b)

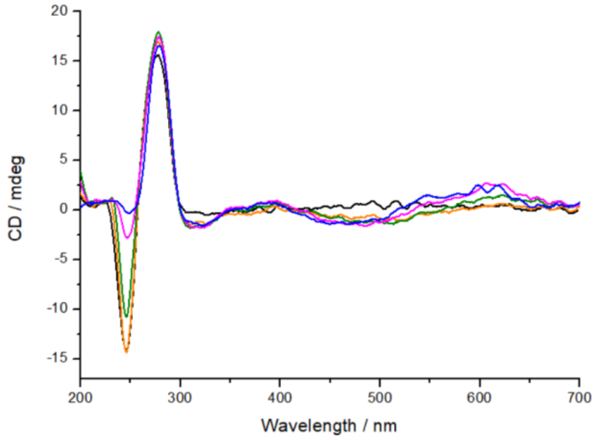

Figure S19. Circular dichroism spectra of ct-DNA (C_{DNA} = 0.1 mM b.p.) in the absence and presence of styryl dyes **6a** (DASPI) (a) and **6b** (b) at different LDR C_{Dye}/C_{DNA}: 0 (black); 0.1 (orange); 0.3 (green); 0.6 (magenta); 1 (blue).

a)

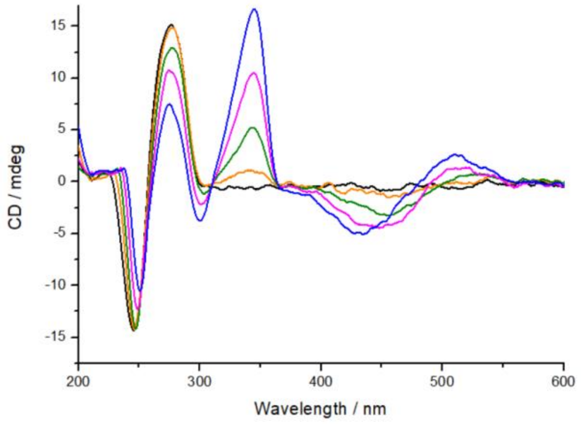

b)

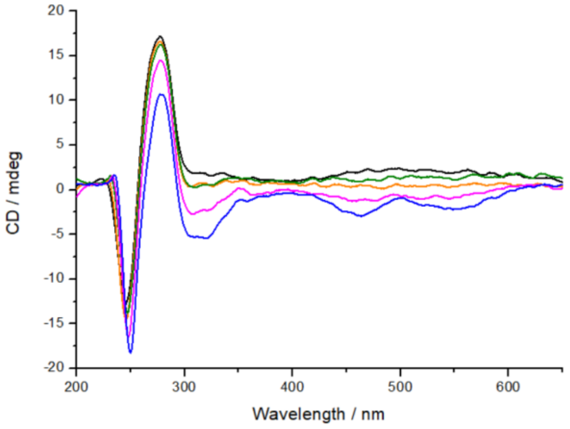

Figure S20. Circular dichroism spectra of ct-DNA (C_{DNA} = 0.1 mM b.p.) in the absence and presence of styryl dyes **7a** (a) and **7b** (b) at different LDR C_{Dye}/C_{DNA}: 0 (black); 0.1 (orange); 0.3 (green); 0.6 (magenta); 1 (blue).

a) b)

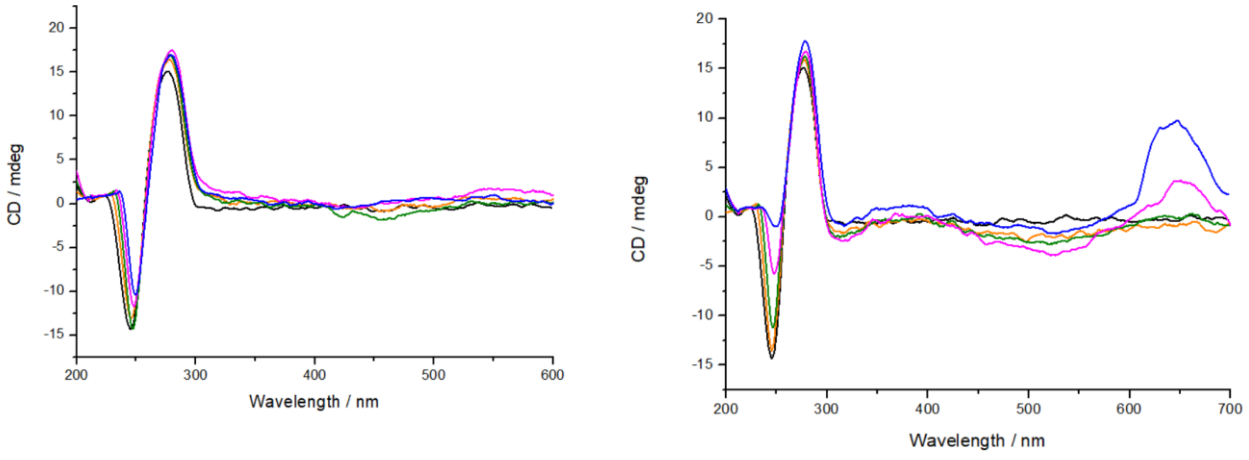

Figure S21. Circular dichroism spectra of ct-DNA (C_{DNA} = 0.1 mM b.p.) in the absence and presence of styryl dyes **8a** (a) and **8b** (b) at different LDR C_{Dye}/C_{DNA}: 0 (black); 0.1 (orange); 0.3 (green); 0.6 (magenta); 1 (blue).

a) b)

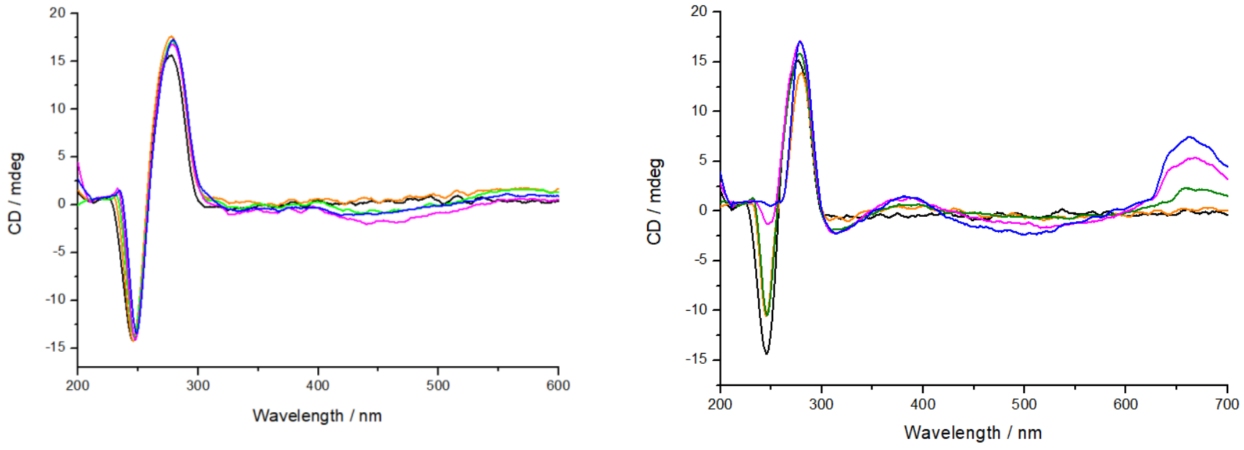

Figure S22. Circular dichroism spectra of ct-DNA (C_{DNA} = 0.1 mM b.p.) in the absence and presence of styryl dyes **9a** (a) and **9b** (b) at different LDR C_{Dye}/C_{DNA}: 0 (black); 0.1 (orange); 0.3 (green); 0.6 (magenta); 1 (blue).

5. Cytotoxicity assays

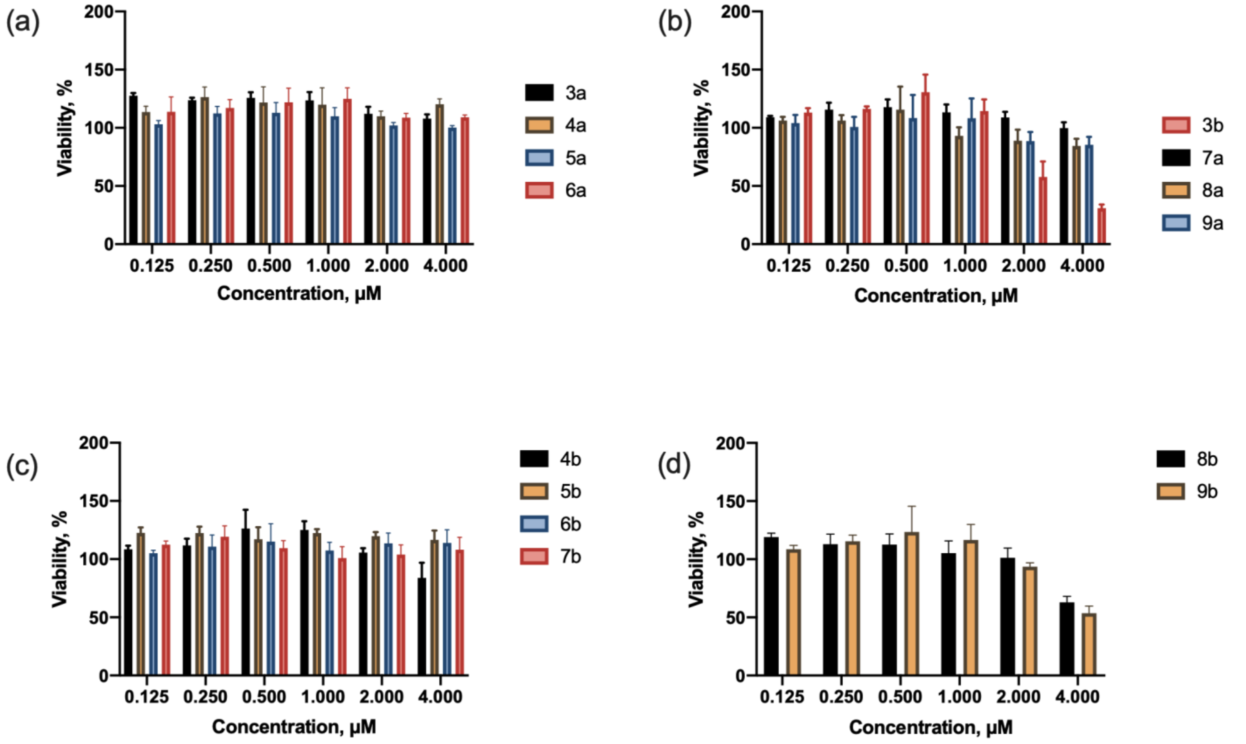

Figure S23. Cell survival of HeLa cells treated with different concentrations of **3a-9a** and **3b-9b** dyes (a-d). Data are presented as mean SD made in three replicates, relative to the control samples.

6. Confocal microscopy

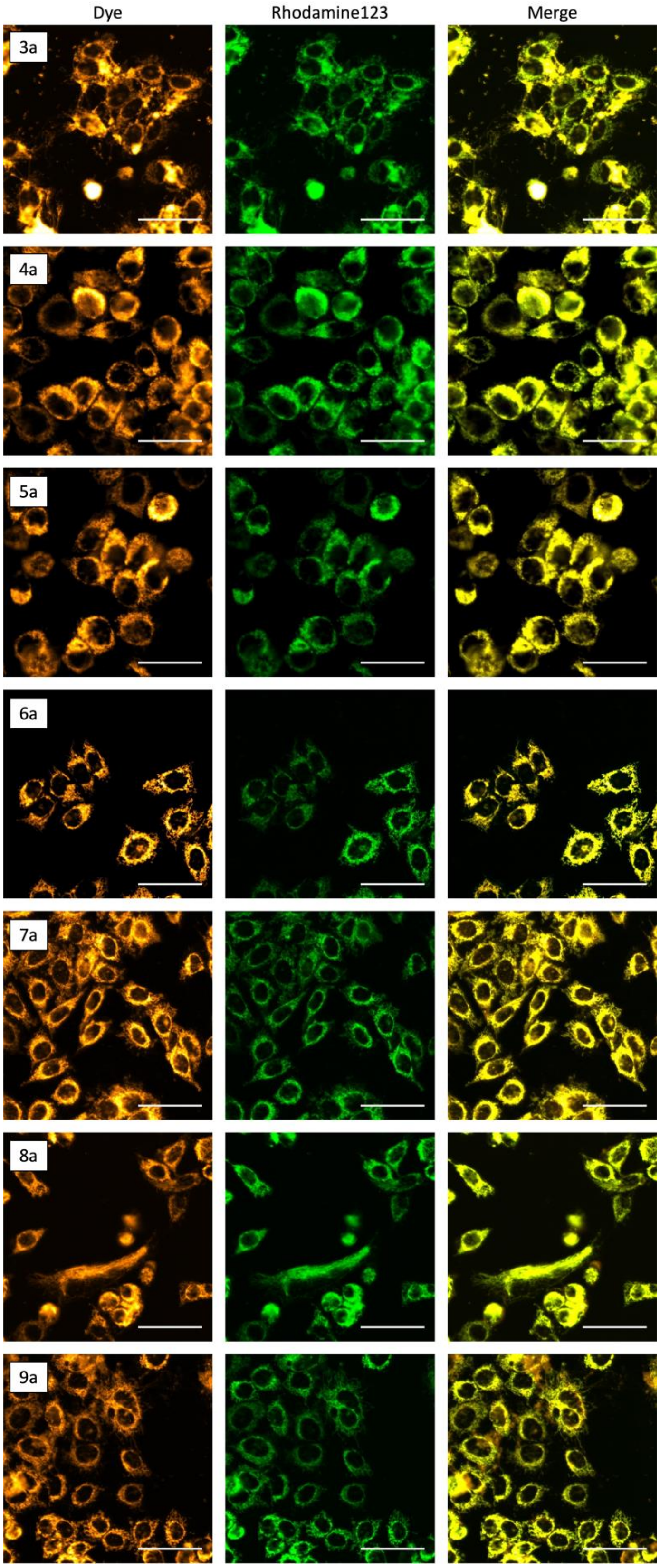

Figure S24 Fluorescence confocal images of HeLa live cells. Orange hot channel is corresponding **3a-9a** dyes (1 μM) (λ_{ex} = 488 nm, detection 575-625 nm); green channel is corresponding Rhodamine 123 (250 μg/mL) (λ_{ex} = 488 nm, detection 500-545 nm). Laser scanning confocal microscopy, scale bar 50 μm.

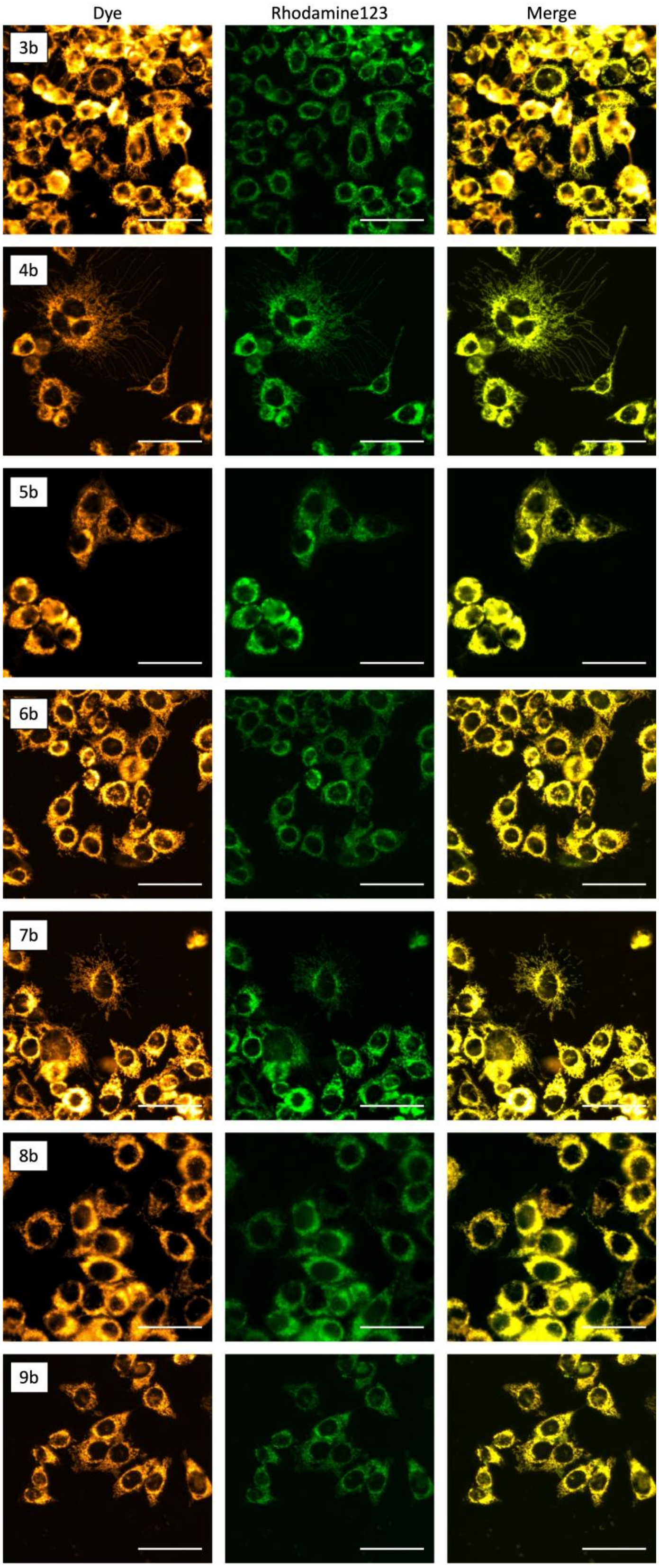

Figure S25 Fluorescence confocal images of HeLa live cells. Orange hot channel is corresponding **3b-9b** dyes (1 μM) (λ_{ex} = 488 nm, detection 575-625 nm); green channel is corresponding Rhodamine 123 (250 μg/mL) (λ_{ex} = 488 nm, detection 500-545 nm). Laser scanning confocal microscopy, scale bar 50 μm.
